# Environmental DNA filtration techniques affect recovered biodiversity

**DOI:** 10.1038/s41598-018-23052-8

**Published:** 2018-03-16

**Authors:** Markus Majaneva, Ola H. Diserud, Shannon H. C. Eagle, Erik Boström, Mehrdad Hajibabaei, Torbjørn Ekrem

**Affiliations:** 1 Department of Natural History, NTNU University Museum, Norwegian University of Science and Technology, NO-7491 Trondheim, Norway; 2 Center for Biodiversity Genomics at Biodiversity Institute of Ontario & Department of Integrative Biology, University of Guelph, Guelph, ON N1G 1Y2 Canada; 3 Norwegian Institute for Nature Research (NINA), NO-7485 Trondheim, Norway

## Abstract

Freshwater metazoan biodiversity assessment using environmental DNA (eDNA) captured on filters offers new opportunities for water quality management. Filtering of water in the field is a logistical advantage compared to transport of water to the nearest lab, and thus, appropriate filter preservation becomes crucial for maximum DNA recovery. Here, the effect of four different filter preservation strategies, two filter types, and pre-filtration were evaluated by measuring metazoan diversity and community composition, using eDNA collected from a river and a lake ecosystem. The filters were preserved cold on ice, in ethanol, in lysis buffer and dry in silica gel. Our results show that filters preserved either dry or in lysis buffer give the most consistent community composition. In addition, mixed cellulose ester filters yield more consistent community composition than polyethersulfone filters, while the effect of pre-filtration remained ambiguous. Our study facilitates development of guidelines for aquatic community-level eDNA biomonitoring, and we advocate filtering in the field, using mixed cellulose ester filters and preserving the filters either dry or in lysis buffer.

## Introduction

Freshwater ecosystem assessments based on morphologically identified macroinvertebrate communities are an essential part of water quality management^1,2^. Although a powerful approach^3,4^, morphological identification of many early-life stages of species is difficult or impossible below the family level; frequently leaving species-rich groups with considerable explanatory potential unutilized^5^. Moreover, sorting and morphological identification is time consuming and strongly dependent on the level of expertise and taxonomic tradition (e.g. availability of taxonomic keys for a specific group, and location). Thus, subsampling is often implemented and the same samples can produce different taxonomic lists depending on the identifier and the subsamples^6,7^. The use of short, standardized DNA sequences to identify species, i.e. DNA barcoding^8^, can overcome many of the aforementioned problems given well-populated reference libraries^9,10^, and high-throughput parallel sequencing of DNA from bulk samples (i.e. DNA metabarcoding) can further increase efficiency and reduce cost of identification, potentially revolutionizing macroinvertebrate-based assessments^11,12^.

In addition to bulk samples, the DNA metabarcoding approach may be applied to genetic material that is obtained directly from the environment, referred to as environmental DNA (eDNA)^13^. This eDNA refers to DNA from microscopic organisms, detached cells, and free DNA released from living cells. The eDNA can be harvested using centrifugation or filtration^14^, and the marker genes (e.g. DNA barcode gene) amplified, sequenced and compared with a reference library in the same way as using DNA extracted from the bulk specimen samples. The eDNA approach has been applied when studying the presence of endangered or invasive species^15–17^, but it also shows the potential to document communities^13,18–21^.

Each step of the eDNA metabarcoding workflow requires critical considerations before implementation^22^ and has numerous variables that can influence the result. For instance, the capture of water eDNA on a given filter is dependent on factors like pH, organic and inorganic particles, pore size and filtered volume^23^, and rigorous methodological comparisons of the eDNA approach are still needed before it can be implemented, for example in large-scale monitoring efforts associated with the EU Water Framework Directive^2,24^. Here, we focus on eDNA capture from freshwater and its preservation.

Capture of eDNA on filters has been found to be more efficient than precipitation by centrifugation^14,23,25^, but various filter membrane types have been used for eDNA capture^22^. Cellulose nitrate (CN) filters have resulted in the highest DNA yield when compared with polyethene sulfone (PES), polyvinylidene fluoride (PVDF) and polycarbonate (PC) filters^26^ and glass microfiber (GMF) filters were shown to outperform PC filters^23^. In another study, both CN and PES filters yielded higher numbers of DNA copies than polycarbonate track-etch (PCTE) and GMF membrane filters^27^. In addition to membrane type, the filter construction itself can influence DNA capture efficiency: Sterivex-GP capsule filters were recently compared with standard filters and found to outperform PCTE and GMF, but not CN filters as long as DNA was extracted from the filter within the capsule^25^.

Another consideration that needs to be taken into account in some environments is the use of larger pore size filters or pre-filtration as they greatly decreases filtration time in the case of turbid water. Two studies have shown a decrease in DNA recovery with an increase in pore size^23,26^, and the pre-filtration process (i.e. size fractioning of particles through filters of different pore sizes) has previously shown to reduce the amount of eDNA of a target species^28^. However, it is unclear if pre-filtration significantly affects the detected community composition.

Environmental DNA degrades readily in the water^29^, and it is important to reduce the time between sampling and filtering to retrieve as much and as long fragments of DNA as possible from the sample, or add a preservative to the water sample^30^. Filtering water at the collection site has advantages compared to transport of water, e.g. fast eDNA capture and no need for dedicated clean facilities for eDNA filtration^22^. Appropriate filter preservation is then crucial for maximum eDNA recovery as well as for sample replicability. Previous studies have focused on single species detection and compared DNA recovery on filters preserved frozen, in ethanol and in lysis buffer^25,27,31^ or studied the effect of ethanol preservation at room temperature^32^. However, none so far have studied filter preservation dry in silica gel or compared all of these strategies statistically at the community level.

**Figure 1:**
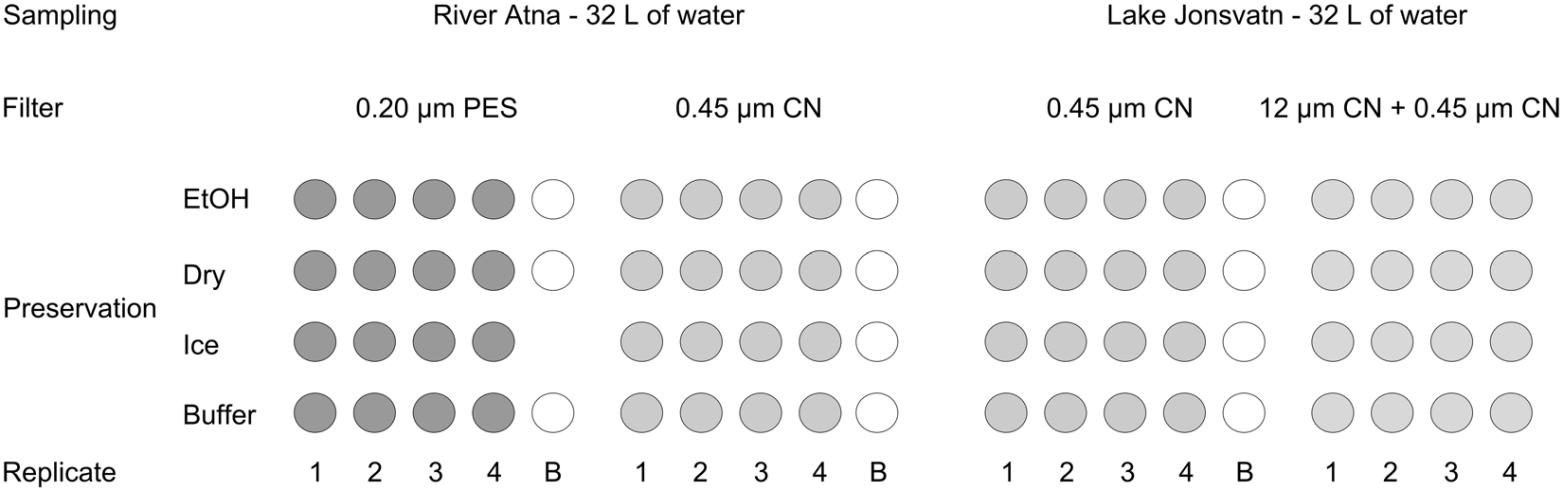
Experimental setup. Water samples were collected from two sites, the River Atna and the Lake Jonsvatn. One litre was filtered and eDNA captured onto 0.20-µm polyethersulfone (PES) or 0.45-µm mixed cellulose ester (CN) filters at the river site. At the lake site, eDNA was captured onto 0.45-µm CN filters either directly or after pre-filtration using 12-µm CN filters. Filters were stored in 99% ethanol (EtOH), silica gel (Dry), Qiagen lysis buffer ATL (Buffer) or kept cold (Ice) until DNA was extracted in the laboratory. 500 mL of molecular grade H_2_O was filtered and the filters stored with the respective methods as negative controls (B).

In this study, we focus on eDNA filtration and filter preservation techniques, using eDNA samples collected from a river and a lake ecosystem. River Atna originates in the Rondane National Park in Central Norway and is a well-documented Nordic freshwater ecosystem^33^. Lake Jonsvatn near Trondheim is a moderately large oligotrophic lake (surface area 15 km^2^, mean depth 37 m) and is the main source for drinking water for the city of Trondheim. Rather than testing the detection of given species, we tested the effect of CN and PES filters, pre-filtration and four different filter preservation strategies on metazoan diversity and community composition. We assumed that we sampled the same community at each site and our null hypothesis was that there is no difference in metazoan diversity or community composition, using different techniques of filtration or preservation. We analysed 85 samples originating from 64 litres of sampled water and 21 negative control samples (Fig. 1) with the intention of finding best-practise protocols for community-level eDNA metabarcoding investigations.

## Results

### DNA concentration and metazoan diversity

We measured DNA concentrations in the samples, using the dsDNA HS Assay Kit in Qubit 2.0. The DNA concentrations were 0.9–6.9 ng µL^−1^ and 1.3–6.5 ng µL^−1^ in the river and lake samples, respectively (Supplementary Fig. S1). DNA concentrations were under the detection limit (0.0005 ng µL^−1^) in the negative samples, except in one negative buffer method sample from the river site which had a detected DNA concentration of 0.02 ng µL^−1^.

We built Nextera XT libraries from the eDNA captured from a total of 64 litres of water collected from a river and a lake ecosystem in Norway. A total of 10.3 million reads were generated on an Illumina MiSeq platform. After merging and quality filtering, 3.9 million good-quality paired-end reads remained for both our amplicons, the 239 bp-long F230 fragment at the 5′ end and the 310 bp-long BE fragment at the 3′ end of the standard COI DNA barcode region^34^. The good-quality reads were clustered into 8736 F230-OTUs and 15609 BE-OTUs at 97% similarity. From those, 2192 F230-OTUs and 1994 BE-OTUs were affiliated with Metazoa in an NCBI BLAST search and from those, 1108 F230-OTUs and 831 BE-OTUs were taxonomically assigned to a metazoan species in BOLD. When combining taxonomic assignments from both the fragments (OTUs assigned to the same taxonomic name were merged into one), our data included 921 taxonomically assigned so called DNA-species^34^.

**Figure 2:**
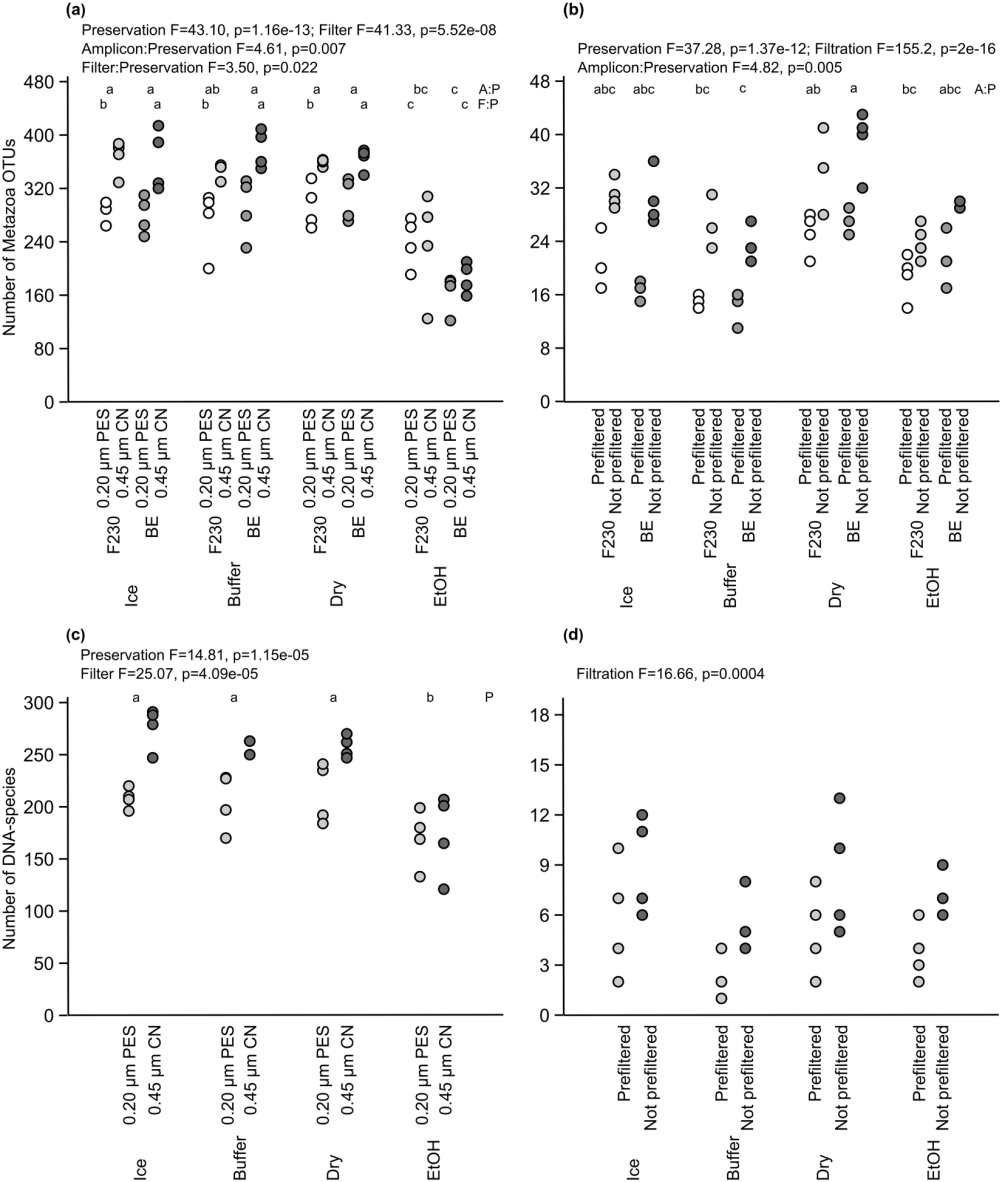
Total number of the Metazoa OTUs (**a**,**b**) and DNA-species (**c**,**d**) in the samples collected from River Atna (**a**,**c**) and Lake Jonsvatn (b, d). The filters were preserved on ice (Ice), in Qiagen ATL lysis buffer (Buffer), on silica gel (Dry) or in 99% ethanol (EtOH). At the river site, 0.20-µm polyethersulfone (PES) or 0.45-µm mixed cellulose ester (CN) filters were used. At the lake site, the samples were either filtered directly onto 0.45-µm CN filters or pre-filtered through 12-µm CN filters before eDNA capture onto 0.45-µm CN filters. Three and two-way ANOVA followed by Tukey’s HSD was used to test differences among the methods, and the F-statistic value (F) and significance (p) are given for significantly different treatments. The small letters denote significantly different groupings of treatments based on the amplicon and preservation and filter and preservation interactions (**a** and **b**) or based on preservation (**c**).

The average number of F230-Metazoa OTUs was 300/sample (range 125–387/sample, 1776 in total) and that of BE-Metazoa OTUs was 291/sample (122–414, 1773) at the river site. The average number of DNA-species was 221/sample (121–291, 859). The number of OTUs and DNA-species per sample was significantly lower in the ethanol-preserved filters than in the dried, cooled and buffer-preserved filters and was lower in PES filters than in CN filters when using the dried, cooled and buffer-preserved filters (Fig. 2a and c; three-way ANOVA followed by Tukey’s pair-wise comparisons, N = 64, *F*(1,3) = 3.50, *p* = 0.022 on filter:preservation interaction using OTUs, two-way ANOVA followed by Tukey’s pair-wise comparisons, N = 32, *F*(1,3) = 14.81, *p* = 1.15e-05 on preservation and N = 32, *F*(1,3) = 25.07, *p* = 4.09e-05 on filter using DNA-species). Simpson index and Pielou’s evenness did not differ among preservation strategies or between filter types at the river site (Supplementary Fig. S2, three-way ANOVAs, *p* > 0.05).

At the lake site, the average number of F230 and BE Metazoa OTUs was 25/sample (F230: 14–41, 155; BE: 11–43, 156), and that of DNA-species was 6/sample (1–13, 90). The buffer preserved filters yielded a significantly lower number of BE-Metazoa OTUs/sample than dried filters (Fig. 2b, three-way ANOVA followed by Tukey’s pair-wise comparisons, N = 64, *F*(1,3) = 4.82, *p* = 0.005 on amplicon:preservation interaction), but there was no difference in the number of F230-OTUs/sample or at the DNA-species level (Fig. 2d, two-way ANOVA, *p* > 0.05). The Simpson index was significantly lower in cooled and buffer preserved filters than dried filters (Supplementary Fig. S2, three-way ANOVA followed by Tukey’s pair-wise comparisons, N = 64, *F*(1,3) = 4.72, *p* = 0.006 on preservation). Pre-filtration lowered the number of OTUs and DNA-species but raised Simpson index and Pielou’s evenness (Fig. 2b and d and Supplementary Fig. S2; three-way ANOVA followed by Tukey’s pair-wise comparisons, N = 64, *F*(1,3) = 155.2, *p* = 2e-16 on filtration using OTUs, two-way ANOVA followed by Tukey’s pair-wise comparisons, N = 32, *F*(1,3) = 16.66, *p* = 0.0004 on filtration using DNA-species, three-way ANOVAs followed by Tukey’s pair-wise comparisons, N = 64, *F*(1,3) = 4.48, *p* = 0.039 and N = 64, *F* (1,3) = 13.13, *p* = 0.0007 on filtration for Simpson index and Pielou’s evenness, respectively).

Based on the number of OTUs and DNA-species as well as the calculated diversity indices, the best performance was achieved with dry, buffer and cool preservation and CN filters at the river site and with dry preservation and direct filtration at the lake site.

### Community composition

**Figure 3:**
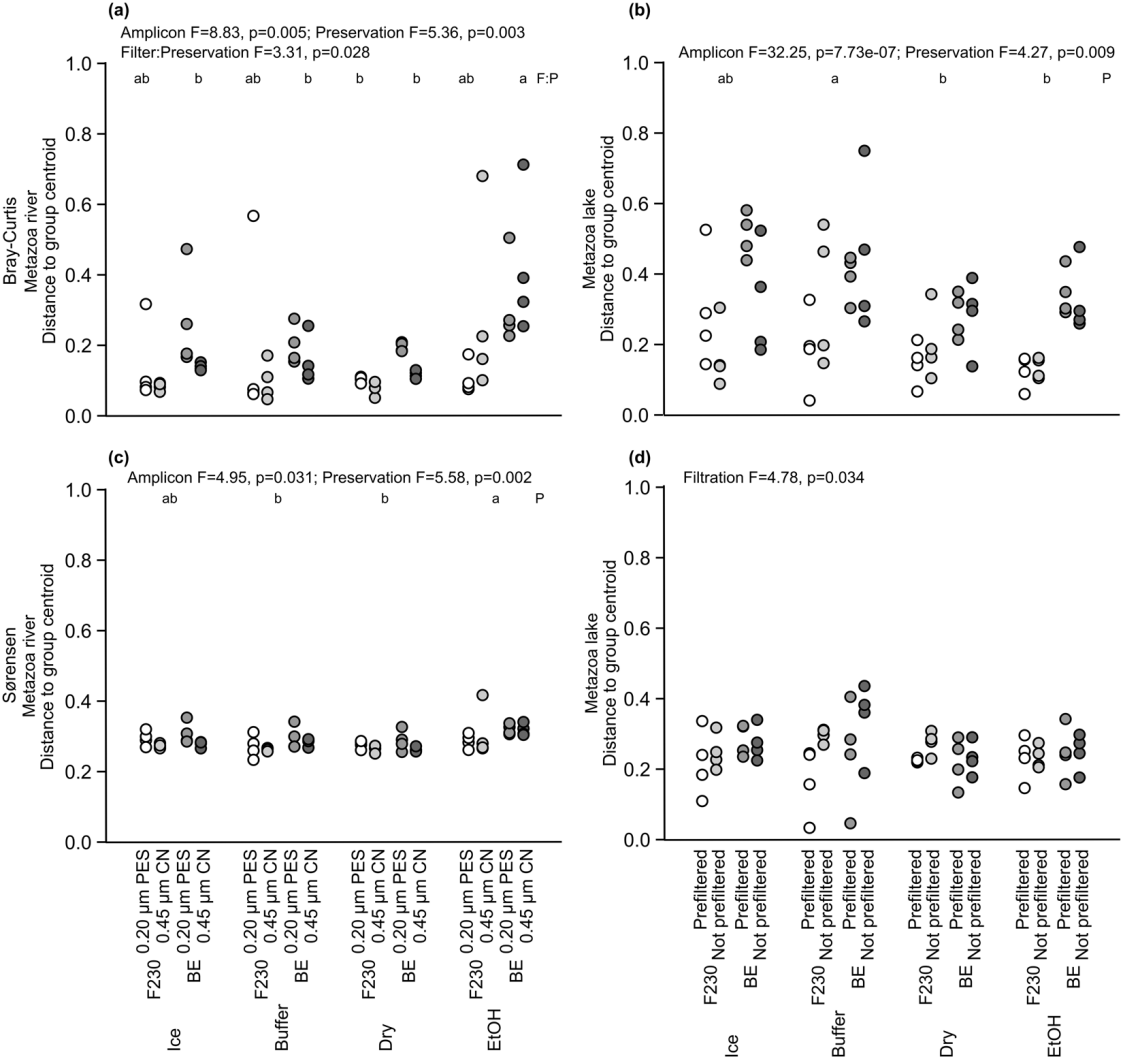
Distance to group centroid in principal coordinate space based on Bray-Curtis similarity (**a**,**b**) and Sørensen dissimilarity (**c**,**d**) calculated for Metazoa OTUs collected from River Atna (**a**,**c**) and from Lake Jonsvatn(**b**,**d**). The filters were preserved on ice (Ice), in Qiagen ATL lysis buffer (Buffer), on silica gel (Dry) or in 99% ethanol (EtOH). At the river site, 0.20-µm polyethersulfone (PES) or 0.45-µm mixed cellulose ester (CN) filters were used. At the lake site, the samples were either filtered directly onto 0.45-µm CN filters or pre-filtered through 12-µm CN filters before eDNA capture onto 0.45-µm CN filters. Three-way ANOVA followed by Tukey’s HSD was used to test differences among the methods, and the F-statistic value (F) and significance (p) are given for significantly different treatments. The small letters denote significantly different groupings of treatments based on the filter and preservation interaction (**a**) or based on preservation (**b** and **c**).

Insecta was the richest group (86% of the DNA-species) at the river site, followed by Collembola (5%), Clitellata (2%) and Arachnida (2%). Community composition from CN and PES filters and from ethanol preserved filters were significantly different (two-way PERMANOVA followed by pair-wise comparisons, N = 32, *F*(1,3) = 1.90, *p* = 0.005 on filter and N = 32, *F*(1,3) = 1.68, *p* = 0.0003 on preservation, Sørensen dissimilarity). This difference in the community composition was due to more variability on PES and ethanol-preserved filters than on CN and dried and buffer-preserved filters (Fig. 3a and c, three-way ANOVAs followed by Tukey’s pair-wise comparisons, N = 64, *F*(1,3) = 3.31, *p* = 0.028 on filter:preservation interaction and Bray-Curtis similarity and N = 64, *F*(1,3) = 5.58, *p* = 0.002 on preservation and Sørensen dissimilarity).

**Figure 4:**
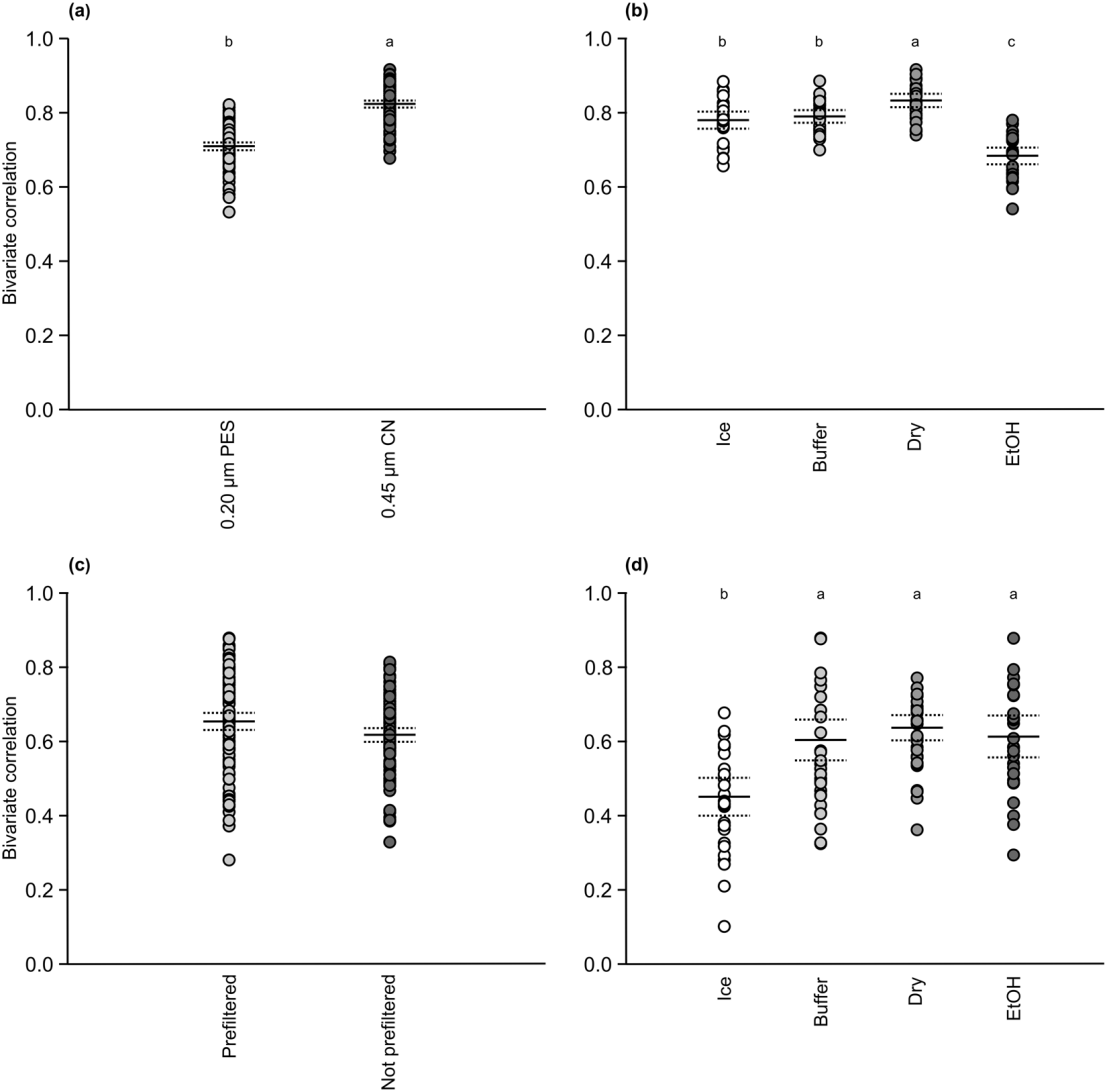
Similarity of metazoan community composition in pairs of samples using the bivariate Poisson-lognormal OTU abundance distribution in River Atna (**a**,**b**) and in Lake Jonsvatn (**c**,**d**). The filters were preserved on ice (Ice), in Qiagen ATL lysis buffer (Buffer), on silica gel (Dry) or in 99% ethanol (EtOH). At the river site, 0.20-µm polyethersulfone (PES) or 0.45-µm mixed cellulose ester (CN) filters were used. At the lake site, the samples were either filtered directly onto 0.45-µm CN filters or pre-filtered through 12-µm CN filters before eDNA capture onto 0.45-µm CN filters. The solid line gives the mean similarity and the dashed lines give the 95% confidence intervals. The small letters denote significantly different groupings of treatments.

When evaluating consistency of sampling in more detail based on the bivariate Poisson-lognormal correlations, i.e. calculating a correlation value based on the community composition for all pairs of samples within the treatments^35–37^, the CN filtering approach represented the community composition more consistently, i.e. had statistically higher mean bivariate correlation (similarity) than the PES; correlations being 0.823 (95% confidence interval 0.813–0.832, N = 240) and 0.709 (0.698–0.719) for the CN and PES filter, respectively (Fig. 4a). Ethanol preservation gave the most heterogeneous samples (Fig. 4b; mean correlation 0.684, 95% confidence interval 0.661–0.706, N = 112). The filters dried on silica gel had statistically highest similarities (0.833, 0.815–0.851), followed by the filters stored on lysis buffer (0.790, 0.773–0.807) and on ice (0.780, 0.757–0.803).

At the lake site, 59% of the DNA-species were Insecta, 13% Clitellata, 8% Maxillopoda, 4% Branchiopoda and 4% Malacostraca. Community composition was significantly different without and with pre-filtration and on dried and ethanol-preserved filters (two-way PERMANOVA followed by pair-wise comparisons, N = 32, *F*(1,3) = 7.76, *p* = 0.0001 on filtration and N = 32, *F*(1,3) = 2.54, *p* = 0.0001 on preservation and BE-OTUs, Sørensen dissimilarity). These differences may be attributed to a greater variation in community composition without pre-filtration and on buffer-preserved filters than with pre-filtration and on dried and ethanol-preserved filters (Fig. 3b and d, three-way ANOVAs followed by Tukey’s pair-wise comparisons, N = 64, *F*(1,3) = 4.27, *p* = 0.009 on preservation and Bray-Curtis similarity and N = 64, *F*(1,3) = 4.78, *p* = 0.034 on filtration and Sørensen dissimilarity).

For the lake Metazoa OTUs, the bivariate similarities were generally lower than for the river samples. For pairs of samples filtered the same way, pre-filtered samples were statistically more similar (0.654, 0.631–0.677N = 240) than samples not pre-filtered (0.618, 0.599–0.636; Fig. 4c). Among the preservation strategies, dried (0.637, 0.603–0.671, N = 112), ethanol-preserved (0.613, 0.557–0.670) and buffer-preserved filters (0.604, 0.549–0.659) were statistically similar, and cooled filters statistically different (0.451, 0.400–0.502; Fig. 4d).

Based on the community composition, the best performance was achieved with dry preservation and CN filters at the river site, and with dry, buffer and ethanol preservation and pre-filtration at the lake site.

## Discussion

This study was based on sampling 64 L of water from well-documented Norwegian river and lake ecosystems, and our experimental set-up produced comprehensive data on how eDNA filtration techniques affect freshwater DNA metabarcoding of Metazoa. Metabarcoding based on eDNA samples holds great potential for larger-than-ever scale monitoring of freshwaters. Despite vivid research and reviews on the different issues relating to eDNA sampling and analysis techniques^22^, best practice protocols are still under development^24^, and few community-level eDNA metabarcoding investigations exist thus far (recently reviewed in Deiner *et al*.^13^). Here, our aim was to contribute to this field. We demonstrate that the choice of filter, pre-filtration and filter preservation strategy have an effect on the metazoan diversity and community composition.

### Experimental set-up

Rare taxa will always be detected stochastically in individual samples unless the sampling effort is increased beyond all practical means^33^, but for detecting given species using eDNA, adding technical PCR replicates increases detection probability considerably and alleviates biases related to PCR and indexing^13,38–40^. Optimal level of (pseudo-)replication and sample volume depends on the question, sample site and community, but a minimum volume of 1 L has been recommended combined with at least 14 μL of extracted eDNA for detecting given species^40^. For monitoring metazoan diversity of a site, it may be more beneficial to sample several occasions during the year to cover the variance for the whole year (see Bista *et al*.^20^ for an eDNA example) than sampling larger volumes in one time point. In addition, true replicates are necessary to explain variability among units of comparison^41^, and since we were not interested in the sensitivity of the eDNA method *per se*^39^ but patterns of potential differences in metazoan community composition in one time point, we focused our effort on taking four 1-L replicates with each experimental unit rather than technical PCR replication.

One small individual may contribute more DNA to the sample than eDNA adding stochasticity to the detection of taxa using eDNA. For example, one ethanol-preserved CN filter included presumably a piece of *Orthocladius telochaetus* (Diptera: Chironomidae) as the sample was totally dominated by reads of that species (28889/33730) which lowered the diversity measures and community similarities (Figs 2–4). The rare taxa also bias the traditional Sørensen-type similarity values: a large difference in sample size may give many rare species in one sample and few in the other, resulting in low Sørensen and Bray-Curtis similarity index values even for replicate samples^37^. Normalization of the number of reads/sample is commonly used to counteract the difference in sequencing (sampling) effort in metagenetic studies^42^, but this does not improve the performance of the traditional similarity indices^37^.

Therefore, not only did we analyse our samples using average dissimilarity of samples based on Bray-Curtis similarity and Sørensen dissimilarity (as common in ecological studies), but also using the bivariate Poisson-lognormal species abundance model. The latter approach offers several advantages, including serving as a control that the variance in community composition stayed at the same level through time (Supplementary Fig. S4). In addition, the influence of variable sample size, detection/non-detection of rare taxa and other random sampling effects are handled by the bivariate correlation since it makes use of the complete species abundance distributions and assumes Poisson sampling^35–37^. By modelling the log abundances, rare and common taxa are taken into account more equally and the effect of random variation in the most dominant taxa is reduced. Thus, the bivariate correlations provided approximately unbiased estimates of similarities and a more detailed analysis of consistency when sampling communities^36,37^.

Assuming that we sampled from the same communities, using different filtration and preservation strategies, the correlation values should be close to one at both sites^37^. Lower values indicate that there was more variance in the log abundances than expected, i.e. not only random Poisson sampling effects, but also other factors like our treatments or small-scale temporal heterogeneity of the sampled communities that had an effect on the correlation values. However, the bivariate correlation values were constant through the period of sampling in the river site (slight negative trend in the lake, see below) confirming that we were sampling the same community despite small-scale temporal heterogeneity (Supplementary Fig. S4). The higher values from the river site compared to the lake site (Fig. 4, Supplementary Fig. S4) indicate smaller variance and, consequently, better consistency in the representation of the community structure by the river than by the lake samples.

One reason for the higher variation in the lake samples may be due to the fact that they were diluted (1 in 50) due to the presence of PCR inhibitors. This likely directly affected the number of Metazoa OTUs, which were surprisingly low compared to the known diversity in the lake^43,44^. We used Qiagen DNeasy Blood and Tissue kit for DNA extraction, which is not as efficient as other kits at removing PCR inhibitors^23^. Previous studies have found that species specific detectability is reduced by 25% using column purification^45^: and 52% using dilution^46^ for removing PCR inhibitors. Thus, it is likely that the dilution of our lake samples for PCR have affected our results considerably. Another factor influencing the lower similarity and negative trend in the bivariate correlation values (Supplementary Fig. S4) in the lake samples presumably was the time difference in sampling for different preservation strategies, and a cloud of green-algae (probably *Volvox*) that drifted through the sampling location as the water for the dried filters was collected, which corrupted our comparisons to some extent. However, we can directly attribute the effect of pre-filtration in community composition results since we did sample pre-filtration and direct filtration samples for each preservation strategy at the same time.

### Filtration

We chose to filter the samples near the sampling location as filtering on site and immediate filter preservation minimizes time for eDNA decay in the samples. It is beneficial especially when sampling remote cold-water locations in warm, sunny conditions as the rate of eDNA degradation increases under higher temperatures and exposure to UV-light^29,47^ (however, see Robson *et al*.^48^ for a tropical example). The use of an electrical vacuum pump connected to a filter holder manifold guaranteed quick and regular filtering conditions for all of the samples without compromising a sterile workspace too much, thus, combining benefits of both on-site and laboratory filtering.

Our results (Supplementary Fig. S1) are in agreement with previous studies which show that cellulose nitrate or mixed cellulose ester (combination of cellulose nitrate and cellulose acetate) give the highest DNA yield when different filter membrane types are compared for eDNA capture^25–27^. This simply may be because cellulose nitrate and acetate act as electron donors while high-molecular weight DNA is an electron acceptor in an aqueous solution^49^. However, water eDNA capture is more complicated than that from laboratory-made southern blotting samples, and factors like pH, organic and inorganic particles and pore size influence the final DNA yield^23,26,28^.

Small pore size filters (0.20 µm^26,28^, <1.5 µm^23^) have been shown to yield the most eDNA, but they may clog easily in algal blooming or turbid waters. In such conditions, either larger pore-size filter or pre-filtration may be used^48,50^. Pre-filtration increases single species detection probability^48^, and based on our results, gives higher diversity index values (Supplementary Fig. S2) and more consistent community composition (Figs 3–4), but it lowers DNA yield^50^ (Supplementary Fig. S1) and the number of detected taxa (Fig. 2b,d). Also, pre-filtration is more expensive to carry out^48^, and therefore, a larger pore size filter may be preferred over pre-filtration, or alternatively, an increased volume of water to compensate for the pre-filtered DNA. If using a larger pore-size filter, one may use an equation for calculating a target water volume that will yield the same amount of eDNA as a smaller pore-size filter^28^.

### Filter preservation strategy

Preserving eDNA filters in ethanol or in lysis buffer immediately after filtration has been proven beneficial for maximum eDNA recovery and detection of given species^25,32^. The preserved filters may be stored at room temperature for later DNA extraction without loss of DNA and as such require less resources than the more commonly used freezing method^25,27,31^. Thus, ethanol or Longmire’s buffer (a type of lysis buffer) have been recommended for filter preservation in previous studies^25,32^.

Here, we showed that ethanol might be a poor preservative for filters since it produced a lower number of taxa in the river ecosystem (Fig. 2a,c) and a more variable community composition than other preservation strategies (Figs 3–4) despite having a higher concentration of DNA (Supplementary Fig. S1). This may originate from our handling of filters that were retrieved from the tubes for evaporation instead of evaporating the ethanol from the tube containing the filter. Either way, more complicated handling of ethanol-preserved filters is an additional liability and may increase the risk of contamination.

We also preserved filters dry on silica gel, which proved to be a good strategy as it gave the same number of OTUs (Fig. 2) as cooling and buffer preservation in the river site (the lake results may not be representative as discussed above). In addition, samples preserved dry had the most similar, i.e. most consistent, community composition (Fig. 4a). However, dried DNA will be vulnerable to changes in conditions if stored for longer periods^51^.

## Conclusions

One universal eDNA capture or preservation method may not be the suitable for all studies because of different physical and chemical characteristics of the study environments^13,22,46^, but our results support the use of cellulose nitrate or mixed cellulose ester filter membranes to capture eDNA^26^, while the choice whether to pre-filter or not remains more elusive and dependent on the specific conditions. Further, we recommend preserving filters either dry on silica gel or in a lysis buffer, which give coherent results both for species detection^25,27,31,32^ and community composition (Figs 2–4), although lakes and rivers differ in their environmental factors. Drying may be preferred if the DNA extraction protocol is not known during sampling but the extraction will take place in the near future, while the use of lysis buffer simplifies the extraction process and samples may be stored for several weeks^31^.

## Methods

### Sampling

Water was collected from two sites in Norway: river Atna at Dørålseter (August 11 2015, N 61.99347°, E 09.80343°, 1032 m above sea level) and lake Jonsvatn at Jonsborg (September 28 2015, N 63.39569°, E 10.55370°, 150 m above sea level).

At each of the two sites, water was collected by submerging sterile 2-L (river site) or 1-L (lake site) rectangular polyethylene terephthalate bottles (Nalgene/VWR International, Radnor, PA, USA) just below the surface. At the river site, water was collected upstream of where the collector was standing, and care was taken not to place feet in the water. Sixteen 2-L samples were collected within two hours. At the lake site, 32 1-L samples were collected from the end of the pier within four hours, with only bottle and gloved hand touching the water. Water was not collected at one time point since it was important to keep time between sampling and filtering about the same for all samples to ensure similar conditions for possible eDNA decay in the samples^29^.

### Sample filtration and DNA extraction

One litre of water was used as a unit of comparison (alias sample), and all samples were filtered on site by using an electrical vacuum pump connected to a manifold (Pall Laboratory, Port Washington, NY, USA) carrying three individually operated filter holder bases. At the river site, two types of filters were used: 0.2 µm polyethersulfone (PES) (Pall Laboratory) and 0.45 µm mixed cellulose ester (CN) (Pall Laboratory) filters. These two types were chosen as easy-to-use in the field because they are attached to a 300-mL reservoir, sterile and individually packed. At the lake site, 0.45 CN filters were used and the effect of pre-filtration using a 12 µm CN (Whatman, GE Healthcare Life Sciences, Chicago, IL, USA) filters was tested.

Four replicate filters were stored in ethanol, in Qiagen ATL lysis buffer (Qiagen GmbH, Hilden, Germany; sold standalone), on ice and dried on silica gel, using 0.2-µm PES and 0.45-µm CN filters at the river site and 0.45-µm CN with or without pre-filtration at the lake site (Fig. 1). As negative control, 500 mL molecular grade water was processed using each preservation strategy. The filters were carefully folded and put into 1.5-mL centrifuge tubes (ethanol, lysis-buffer and on-ice strategies) or unfolded onto a sterile petri dish and into a quick-zip plastic bag with 100 g of silica gel (dry strategy). 1.5 mL of 99% molecular grade ethanol and 1.0 mL of Qiagen buffer ATL were added to the tubes in the ethanol and lysis-buffer strategy, respectively. The tubes in the on-ice strategy were kept on ice for 3–5 hours until placed at −20 °C for one week before DNA extraction. The ethanol preserved, lysis-buffer preserved and dried filters were kept in the dark at room temperature for one week before DNA extraction. Prior to DNA extraction, the lysis buffer from the lysis-buffer preserved filters was divided into two tubes each containing 500 µL; the dry and on-ice preserved filters were submerged in 500 µL of buffer ATL in tubes; the ethanol preserved filters were carefully removed from the tubes, dried on sterile petri dishes, refolded and put back into 1.5 mL tubes with 500 µL of buffer ATL. The DNEasy Blood and Tissue kit (Qiagen) was used for DNA extraction. Samples were incubated with 50 µL of proteinase K overnight at 37 °C on a rocking platform (200 rpm). The remaining extraction followed the standard manufacturer’s protocol, except the DNA was eluted with 100 µL of elution buffer. The extracted DNA was quantified with Qubit 2.0 (Invitrogen, CA, USA), using the dsDNA HS Assay Kit according to the manufacturer’s protocol.

### PCR amplification and sequencing

We amplified two regions of interest: the 239 bp F230 fragment at the 5′ end and the 310 bp BE fragment at the 3′ end of the standard COI DNA barcode region. The F230 fragment was amplified, using the LCO1490 forward primer (GGTCAACAAATCATAAAGATATTGG)^52^ and the 230_R reverse primer (CTTATRTTRTTTATICGIGGRAAIGC)^34^, and the BE fragment was amplified with the B forward primer (CCIGAYATRGCITTYCCICG)^11^ and the R5 reverse primer (GTRATIGCICCIGCIARIACIGG)^53^. Both fragments were amplified once with attached Illumina (San Diego, CA, USA) adapters 5′-TCGTCGGCAGCGTCAGATGTGTATAAGAGACAG‐3′ (forward) and 5′-GTCTCGTGGGCTCGGAGATGTGTATAAGAGACAG‐3′ (reverse). The PCR reactions had a final volume of 25 μL containing 2 μL DNA template (lake eDNA diluted 1:50 due to PCR inhibition in original concentrations), 17.8 μL molecular biology grade water, 2.5 μL 10× reaction buffer (200 mM Tris HCl, 500 mM KCl, pH 8.4), 1 μL MgCl_2_ (50 mM), 0.5 μL dNTPs mix (10 mM), 0.5 μL forward primer (10 mM), 0.5 μL reverse primer (10 mM), and 0.2 μL Invitrogen’s Platinum Taq polymerase (5 U/μL). All PCRs included negative control reactions (no DNA template). The PCR conditions were, with a heated lid, 94 °C for 5 min, followed by a total of 35 cycles of 94 °C for 40 s, 46 °C for 1 min, and 72 °C for 20 s, and a final extension at 72 °C for 2 min, and hold at 10 °C. PCR products were visualized on a 1.5% agarose gel to check the amplification success and a subset of samples was quantified using PicoGreen (Thermo Fisher Scientific, Waltham, MA, USA) according to the manufacturer’s protocol. In the second step, the Illumina tailed amplicons were dual indexed, using Nextera XT Index 1 and 2 primers (FC-131–1002, Illumina) in a reduced-cycle PCR according to the manufacturer’s protocol. The amplification reactions contained the same reagent concentrations as above with three modifications: 3 µL of amplicon, 1 µL of each primer and 0.25 µL of Platinum Taq (Invitrogen). Indexed amplicons were pooled into a library and sequenced on a flow cell, using the 600-cycle V3 Illumina MiSeq sequencing kit (MS-102-3003).

### Bioinformatic processing

Resulting raw F230 and BE amplicon reads were processed with mothur v.1.36.1^42^. First, the forward and reverse reads were merged keeping only the reads with no mismatch in primer sequence, using the command make.contigs. The F230 (and BE) forward and reverse fragments overlapped on average with 282 bases (238 bases). All F230 (BE) reads shorter than 250 (330) bases and longer than 340 (375) bases as well as reads with ambiguous bases were removed with command screen.seqs. Primer sequences were removed using the command trim.seqs. After the quality control, the resulting good quality reads were processed further, using usearch v8.1.1831_win32^54^. Exact duplicates were removed using the command -derep_fulllength, and the reads were de-noised using a sequence identity threshold of 98% in the command -cluster_otus. The above steps were done for each sample and the resulting operational taxonomic unit (OTU) fasta files were pooled, using merge.files in mothur. The resulting pooled OTU file was de-replicated, using -derep_fulllength, and the final OTUs clustered, using -cluster_otus at 97% similarity level. The abundance of each OTU in each sample was searched using -usearch_global against the pooled OTU-file.

The number of raw reads in the negative extraction controls (n = 10) was 0.4 and 2.2% of the raw reads in the river and lake samples, respectively, and 2.8 and 11.0% in the negative control filters (n = 11), respectively, showing signs of minor cross-contamination both in the PCR and indexing as well as in the field sampling. This was dealt with by removing OTUs that were possible contaminants, i.e. OTUs present only or predominantly in the negative samples (41 F230 OTUs, 44 BE OTUs).

The OTUs were assigned taxonomically in two steps. First, they were searched against the NCBI non-redundant nucleotide database using the BLAST 2.3.0+^55^ (F230 search performed July 21, 2016; BE November 18, 2016). Taxonomic assignment of an OTU was done, using the lowest common ancestor algorithm in MEGAN 6.4.19^56^ (minimum bit score 100, top percentage 8.0 and minimum support 1). The Metazoa OTUs were translated to amino acids and aligned, using Mafft 7 online tool^57^ to remove all non-COI OTUs and OTUs with stop codons in mitochondrial invertebrate code. Secondly, the Metazoa OTUs were assigned taxonomically using BOLD v.4 Species Level Barcode Records (F230 search performed August 19, 2016; BE November 22, 2016; >97% matches were considered as an assignment). To generate a DNA-species subset, the assigned F230 and BE OTUs were pooled and OTUs assigned to the same taxonomic name were merged into one DNA-species^34^. The number of reads/sample was normalized to the lowest number of reads/sample, which was 33730 F230 reads and 26837 BE reads/river sample and 19431 F230 reads and 12720 BE reads/lake sample for the OTU comparisons, and to 60358 reads/river sample and 32151 reads/lake sample for DNA-species comparisons. The non-normalized number of reads and OTUs/sample are listed in the Supplementary Table S1. Simpson index and Pielou’s evenness were calculated using PAST 3.04^58^.

### Statistical analyses

To find statistically significant differences (p < 0.05) in the DNA concentration and metazoan diversity among different filtering and preservation strategies, three-way and two-way ANOVA followed by Tukey’s pairwise comparisons were used. To find statistically significant (p < 0.05) differences in the community composition, two-way PERMANOVA with Bray-Curtis similarity and Sørensen dissimilarity with 9999 permutations was used. In addition, average dissimilarity of samples from their group centroid in principal coordinate space was calculated (Bray-Curtis similarity and Sørensen dissimilarity), using the betadisper function in the R-package vegan^59,60^ and tested with three-way and two-way ANOVA. The data were approximately normally distributed (Shapiro-Wilk W). The test results are listed in the Supplementary Methods. Lake Jonsvatn included 1–13 DNA-species/sample, and DNA-species were used for the community composition comparisons only at the river site. To study the consistency of the filtration and preservation strategies in more detail, similarity in community composition was analysed by fitting the bivariate Poisson-lognormal species (based on F230 OTUs) abundance distribution to pairs of samples^35–37^, using the R-package poilog^60,61^ and taking into account for the effect of covariance of dependent pairs of correlations^62^. These analyses were done, using non-normalized data. See Diserud *et al*.^37^ for a more elaborate description and discussion of the method.

### Data Availability

The dataset generated and analysed during the current study is available in the ENA SRA repository with study name PRJEB21623. Scripts used in the current study are included in the Supplementary information file (Supplementary Methods).

## Electronic supplementary material

Supplementary information
