# Supplementary material for: Environmental DNA filtration techniques affect recovered biodiversity
Source: Sci Rep. 2018 Mar 16;8:4682. doi: 10.1038/s41598-018-23052-8 (PMC5856736; doi:10.1038/s41598-018-23052-8)
Supplement: Supplementary file 1 — Supplementary information [file 41598_2018_23052_MOESM1_ESM.docx]

Environmental DNA filtration techniques affect recovered biodiversity

Markus Majaneva, Ola H. Diserud, Shannon H.C. Eagle, Erik Boström, Mehrdad Hajibabaei & Torbjørn Ekrem


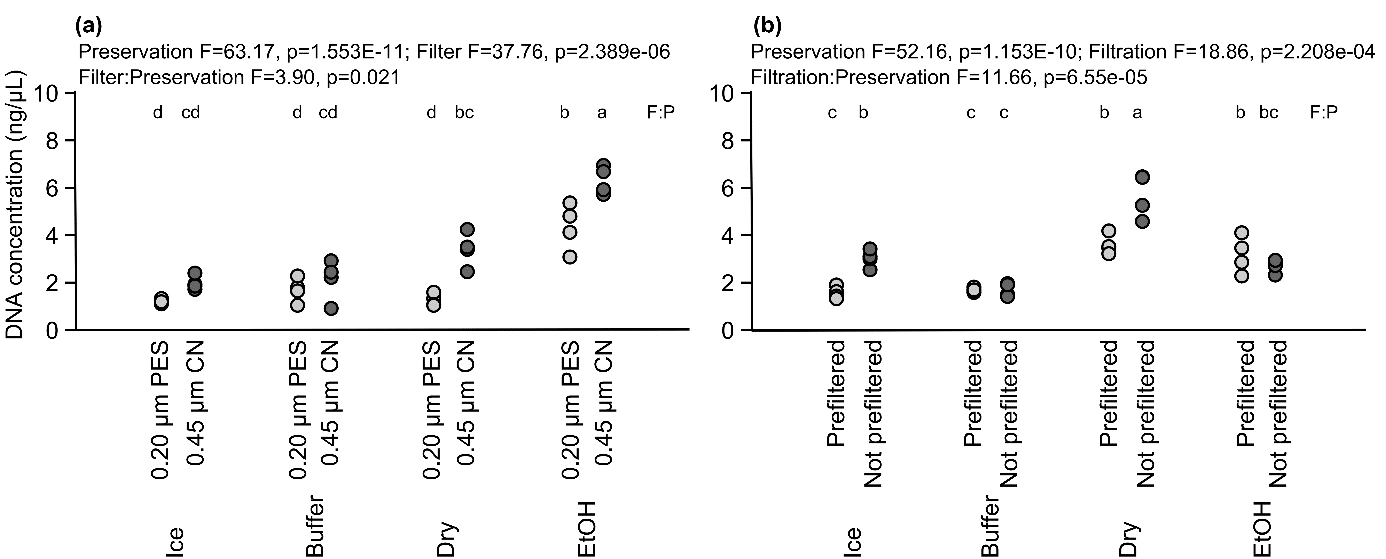


**Supplementary Figure S1** Concentration of DNA in the samples collected from (a) River Atna and (b) Lake Jonsvatn. The filters were preserved on ice (Ice), in Qiagen ATL lysis buffer (Buffer), on silica gel (Dry) or in 99% ethanol (EtOH). At the river site, 0.20-µm polyethersulfone (PES) or 0.45-µm mixed cellulose ester (CN) filters were used. At the lake site, the samples were either filtered directly onto 0.45-µm CN filters or pre-filtered through 12-µm CN filters before eDNA capture onto 0.45-µm CN filters. Two-way ANOVA followed by Tukey’s HSD was used to test differences among the methods, and the F-statistic value (F) and significance (p) are given for significantly different treatments. The small letters denote significantly different groupings of treatments based on the interaction term.


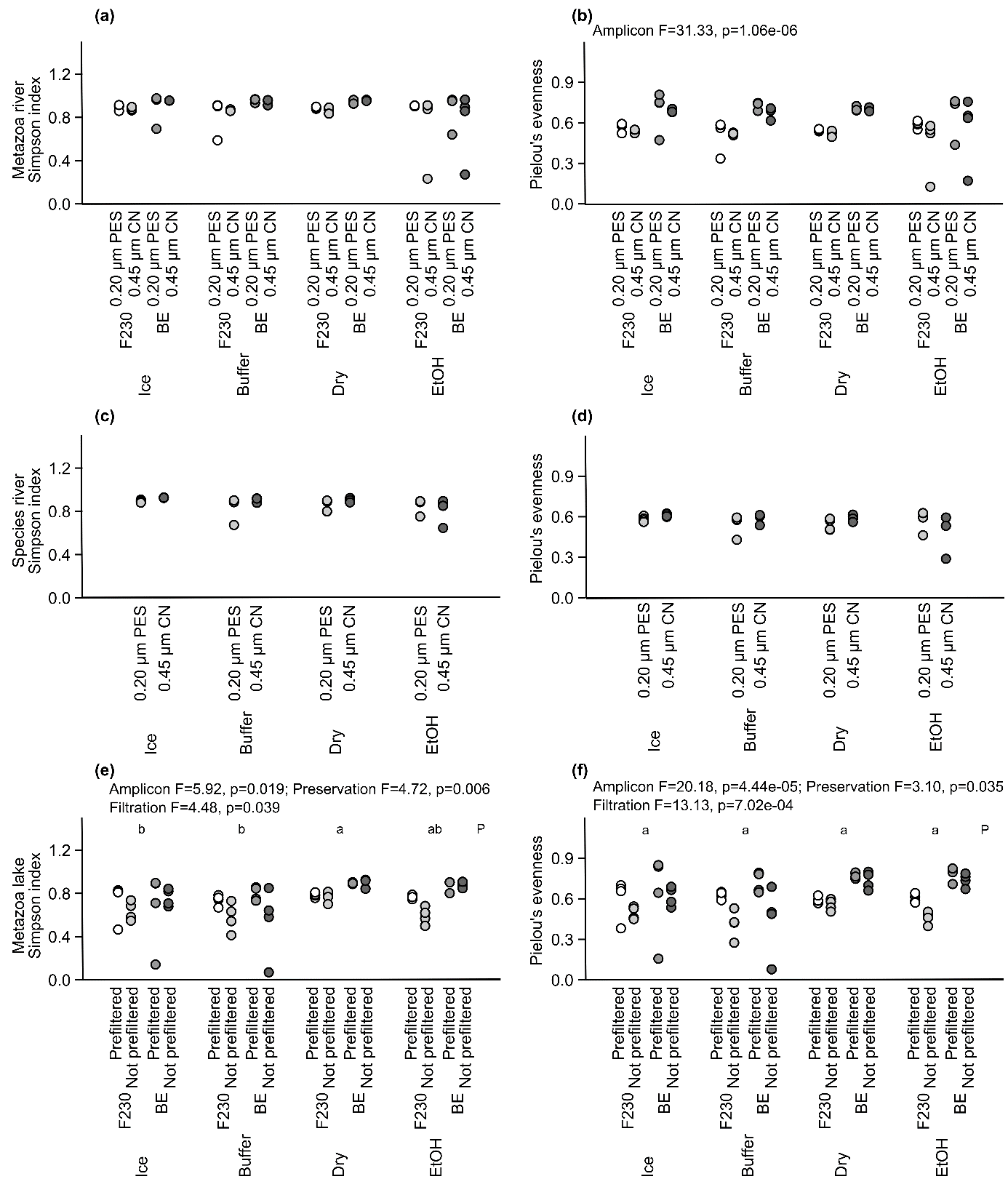


**Supplementary Figure S2** Simpson index and Pielou’s evenness calculated for Metazoa OTUs (a–b) and DNA-species (c–d) collected from River Atna and for Metazoa OTUs from Lake Jonsvatn (e–f). The filters were preserved on ice (Ice), in Qiagen ATL lysis buffer (Buffer), on silica gel (Dry) or in 99% ethanol (EtOH). At the river site, 0.20-µm polyethersulfone (PES) or 0.45-µm mixed cellulose ester (CN) filters were used. At the lake site, the samples were either filtered directly onto 0.45-µm CN filters or pre-filtered through 12-µm CN filters before eDNA capture onto 0.45-µm CN filters. Three and two-way ANOVA followed by Tukey’s HSD was used to test differences among the methods, and the F-statistic value (F) and significance (p) are given for significantly different treatments. The small letters denote significantly different groupings of preservation strategies (e and f).


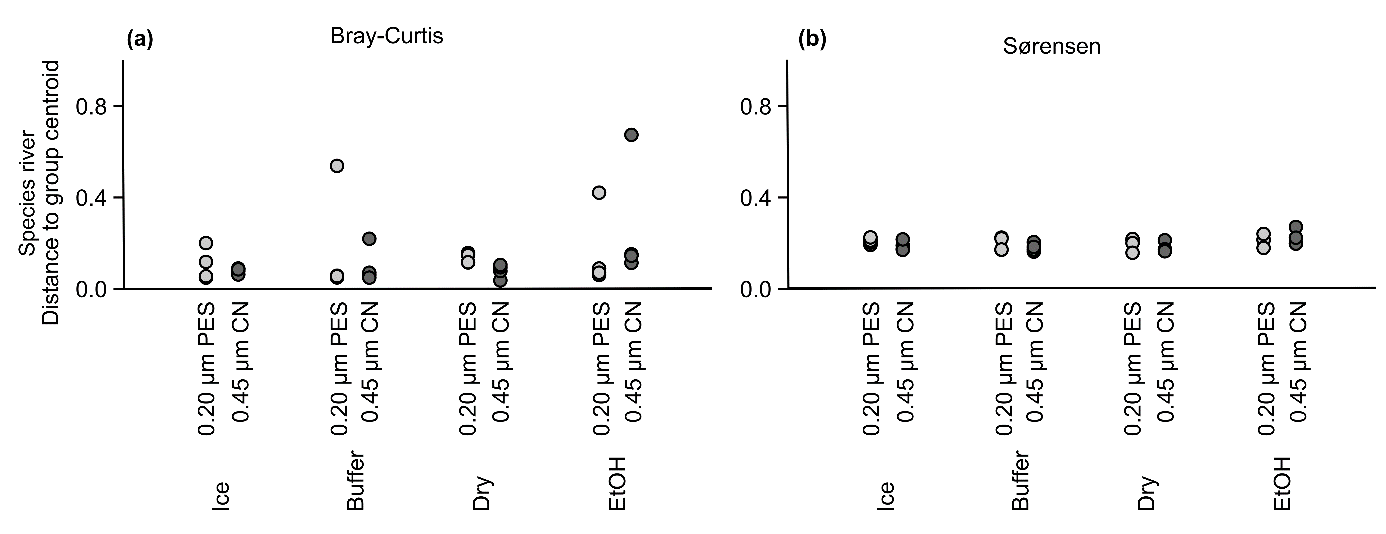


**Supplementary Figure S3** Distance to group centroid in principal coordinate space based on Bray-Curtis similarity (a) and Sørensen dissimilarity (b) calculated for DNA-species collected from River Atna. The filters were preserved on ice (Ice), in Qiagen ATL lysis buffer (Buffer), on silica gel (Dry) or in 99% ethanol (EtOH). At the river site, 0.20-µm polyethersulfone (PES) or 0.45-µm mixed cellulose ester (CN) filters were used. At the lake site, the samples were either filtered directly onto 0.45-µm CN filters or pre-filtered through 12-µm CN filters before eDNA capture onto 0.45-µm CN filters.


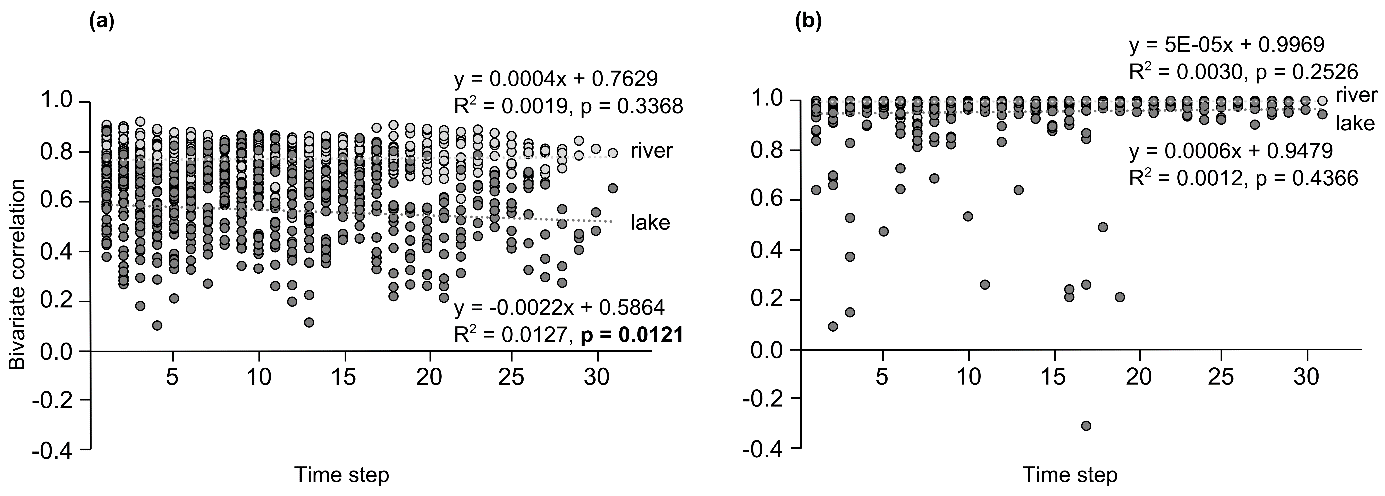


**Supplementary Figure S4** Bivariate correlation values between all pairs of samples plotted against the time difference between pairs of samples. The lighter grey circles denote the river Atna samples and the darker grey circles denote the lake Jonsvatn samples. Bivariate correlation values were calculated based on abundance of F230 OTUs (a) and based on presence/absence of F230 OTUs (b), and the linear trend lines and corresponding correlation statistics are given. The significant negative trend of lake Jonsvatn samples is highlighted in (a).

| **Supplementary Table S1** The non-normalized number of raw reads, good quality (g-q) paired reads, 97-% OTUs, OTUs affiliated with Metazoa and OTUs assigned to metazoan species in BOLD (ID Metazoa OTUs) in the samples (n = 64) and sequenced negative control samples (n = 21). | | | | | | | | | |
| --- | --- | --- | --- | --- | --- | --- | --- | --- | --- |
| Sample information | | | | F230 fragment | | | | |  |
| Sample | Site | Filtration | Preservation | raw reads | g-q paired reads | OTUs | Metazoa OTUs | ID Metazoa OTUs |  |
| JbN451 | Lake | 0.45 µm CN | Buffer | 198694 | 77036 | 201 | 43 | 10 |  |
| JbN452 | Lake | 0.45 µm CN | Buffer | 116826 | 42768 | 201 | 31 | 6 |  |
| JbN453 | Lake | 0.45 µm CN | Buffer | 88218 | 32716 | 156 | 28 | 5 |  |
| JbN454 | Lake | 0.45 µm CN | Buffer | 88688 | 29838 | 140 | 31 | 4 |  |
| JbP451 | Lake | 12 µm CN + 0.45 µm CN | Buffer | 129100 | 47499 | 155 | 20 | 3 |  |
| JbP452 | Lake | 12 µm CN + 0.45 µm CN | Buffer | 166380 | 60932 | 156 | 16 | 0 |  |
| JbP453 | Lake | 12 µm CN + 0.45 µm CN | Buffer | 123568 | 44199 | 155 | 19 | 3 |  |
| JbP454 | Lake | 12 µm CN + 0.45 µm CN | Buffer | 69634 | 19639 | 142 | 14 | 3 |  |
| JdN451 | Lake | 0.45 µm CN | Dry | 149952 | 55831 | 287 | 44 | 7 |  |
| JdN452 | Lake | 0.45 µm CN | Dry | 153698 | 56747 | 325 | 47 | 12 |  |
| JdN453 | Lake | 0.45 µm CN | Dry | 172082 | 63670 | 355 | 48 | 10 |  |
| JdN454 | Lake | 0.45 µm CN | Dry | 244506 | 89697 | 301 | 39 | 7 |  |
| Jdp451 | Lake | 12 µm CN + 0.45 µm CN | Dry | 154600 | 58438 | 292 | 33 | 10 |  |
| Jdp452 | Lake | 12 µm CN + 0.45 µm CN | Dry | 169934 | 61159 | 283 | 23 | 4 |  |
| Jdp453 | Lake | 12 µm CN + 0.45 µm CN | Dry | 187852 | 67239 | 279 | 32 | 7 |  |
| Jdp454 | Lake | 12 µm CN + 0.45 µm CN | Dry | 195322 | 71298 | 311 | 33 | 5 |  |
| JeN451 | Lake | 0.45 µm CN | EtOH | 102138 | 36440 | 191 | 22 | 6 |  |
| JeN452 | Lake | 0.45 µm CN | EtOH | 106102 | 38627 | 179 | 27 | 6 |  |
| JeN453 | Lake | 0.45 µm CN | EtOH | 140058 | 52225 | 243 | 33 | 13 |  |
| JeN454 | Lake | 0.45 µm CN | EtOH | 145754 | 51256 | 234 | 27 | 6 |  |
| Jep451 | Lake | 12 µm CN + 0.45 µm CN | EtOH | 99616 | 35297 | 174 | 26 | 11 |  |
| Jep452 | Lake | 12 µm CN + 0.45 µm CN | EtOH | 142582 | 50784 | 200 | 23 | 6 |  |
| Jep453 | Lake | 12 µm CN + 0.45 µm CN | EtOH | 105780 | 38955 | 190 | 20 | 5 |  |
| Jep454 | Lake | 12 µm CN + 0.45 µm CN | EtOH | 77616 | 27165 | 146 | 16 | 2 |  |
| JiN451 | Lake | 0.45 µm CN | Ice | 131688 | 47847 | 240 | 38 | 10 |  |
| JiN452 | Lake | 0.45 µm CN | Ice | 127028 | 47623 | 226 | 35 | 8 |  |
| JiN453 | Lake | 0.45 µm CN | Ice | 147438 | 55081 | 245 | 37 | 10 |  |
| JiN454 | Lake | 0.45 µm CN | Ice | 156324 | 57783 | 256 | 34 | 6 |  |
| JiP451 | Lake | 12 µm CN + 0.45 µm CN | Ice | 129000 | 47995 | 180 | 19 | 4 |  |
| JiP452 | Lake | 12 µm CN + 0.45 µm CN | Ice | 139816 | 51406 | 192 | 20 | 4 |  |
| JiP453 | Lake | 12 µm CN + 0.45 µm CN | Ice | 155412 | 57240 | 204 | 21 | 6 |  |
| JiP454 | Lake | 12 µm CN + 0.45 µm CN | Ice | 97446 | 36159 | 172 | 29 | 12 |  |
|  |  |  |  |  |  |  |  |  |  |
| **Supplementary Table S1** continued. | | |  |  |  |  |  |  |  |
| Sample information | | | | F230 fragment | | | | |  |
| Sample | Site | Filtration | Preservation | raw reads | g-q paired reads | OTUs | Metazoa OTUs | ID Metazoa OTUs |  |
| DbC1 | River | 0.45 µm CN | Buffer | 161444 | 65416 | 979 | 379 | 270 |  |
| DbC2 | River | 0.45 µm CN | Buffer | 145160 | 59143 | 939 | 389 | 276 |  |
| DbC3 | River | 0.45 µm CN | Buffer | 160976 | 66570 | 986 | 380 | 275 |  |
| DbC4 | River | 0.45 µm CN | Buffer | 154022 | 62863 | 960 | 365 | 254 |  |
| DbP1 | River | 0.20 µm PES | Buffer | 193330 | 75967 | 1068 | 342 | 242 |  |
| DbP2 | River | 0.20 µm PES | Buffer | 184920 | 73370 | 581 | 215 | 168 |  |
| DbP3 | River | 0.20 µm PES | Buffer | 194210 | 75839 | 1182 | 331 | 224 |  |
| DbP4 | River | 0.20 µm PES | Buffer | 186574 | 74168 | 981 | 316 | 229 |  |
| DdC1 | River | 0.45 µm CN | Dry | 141072 | 57152 | 1228 | 398 | 279 |  |
| DdC2 | River | 0.45 µm CN | Dry | 127012 | 51606 | 1075 | 369 | 251 |  |
| DdC3 | River | 0.45 µm CN | Dry | 170072 | 68342 | 1257 | 406 | 283 |  |
| DdC4 | River | 0.45 µm CN | Dry | 102002 | 40664 | 1107 | 361 | 257 |  |
| DdP1 | River | 0.20 µm PES | Dry | 184400 | 74491 | 1041 | 346 | 243 |  |
| DdP2 | River | 0.20 µm PES | Dry | 159282 | 62918 | 1109 | 375 | 268 |  |
| DdP3 | River | 0.20 µm PES | Dry | 204130 | 81000 | 933 | 319 | 229 |  |
| DdP4 | River | 0.20 µm PES | Dry | 227256 | 90740 | 973 | 311 | 221 |  |
| Dec1 | River | 0.45 µm CN | EtOH | 181472 | 71355 | 1455 | 336 | 243 |  |
| Dec2 | River | 0.45 µm CN | EtOH | 218166 | 90463 | 402 | 151 | 118 |  |
| Dec3 | River | 0.45 µm CN | EtOH | 155862 | 59763 | 1180 | 301 | 228 |  |
| Dec4 | River | 0.45 µm CN | EtOH | 247094 | 93816 | 1135 | 276 | 203 |  |
| DeP1 | River | 0.20 µm PES | EtOH | 174230 | 67742 | 1150 | 292 | 220 |  |
| DeP2 | River | 0.20 µm PES | EtOH | 158844 | 59924 | 1260 | 287 | 200 |  |
| DeP3 | River | 0.20 µm PES | EtOH | 184214 | 69752 | 1077 | 254 | 184 |  |
| DeP4 | River | 0.20 µm PES | EtOH | 161728 | 61471 | 904 | 199 | 145 |  |
| Dic1 | River | 0.45 µm CN | Ice | 138776 | 56990 | 775 | 350 | 244 |  |
| Dic2 | River | 0.45 µm CN | Ice | 152778 | 61769 | 862 | 403 | 281 |  |
| Dic3 | River | 0.45 µm CN | Ice | 165808 | 66512 | 971 | 428 | 296 |  |
| Dic4 | River | 0.45 µm CN | Ice | 175670 | 69300 | 1013 | 434 | 306 |  |
| DiP1 | River | 0.20 µm PES | Ice | 171174 | 68456 | 877 | 324 | 237 |  |
| DiP2 | River | 0.20 µm PES | Ice | 207584 | 82204 | 768 | 312 | 222 |  |
| DiP3 | River | 0.20 µm PES | Ice | 194054 | 76751 | 770 | 304 | 209 |  |
| DiP4 | River | 0.20 µm PES | Ice | 214040 | 84533 | 946 | 333 | 225 |  |
|  |  |  |  |  |  |  |  |  |  |
| **Supplementary Table S1** continued. | | |  |  |  |  |  |  |  |
| Sample information | | | | BE fragment | | | | | F230+BE |
| Sample | Site | Filtration | Preservation | raw reads | g-q paired reads | OTUs | Metazoa OTUs | ID Metazoa OTUs | DNA-species |
| JbN451 | Lake | 0.45 µm CN | Buffer | 71948 | 48717 | 158 | 25 | 8 | 11 |
| JbN452 | Lake | 0.45 µm CN | Buffer | 67655 | 46021 | 214 | 28 | 5 | 7 |
| JbN453 | Lake | 0.45 µm CN | Buffer | 65952 | 44968 | 188 | 37 | 9 | 11 |
| JbN454 | Lake | 0.45 µm CN | Buffer | 47925 | 28929 | 166 | 27 | 6 | 5 |
| JbP451 | Lake | 12 µm CN + 0.45 µm CN | Buffer | 53620 | 36198 | 153 | 19 | 4 | 4 |
| JbP452 | Lake | 12 µm CN + 0.45 µm CN | Buffer | 32128 | 21576 | 141 | 15 | 3 | 2 |
| JbP453 | Lake | 12 µm CN + 0.45 µm CN | Buffer | 48549 | 33310 | 142 | 17 | 2 | 5 |
| JbP454 | Lake | 12 µm CN + 0.45 µm CN | Buffer | 28901 | 12931 | 131 | 11 | 1 | 4 |
| JdN451 | Lake | 0.45 µm CN | Dry | 71580 | 49486 | 337 | 50 | 13 | 10 |
| JdN452 | Lake | 0.45 µm CN | Dry | 77010 | 53013 | 327 | 33 | 7 | 13 |
| JdN453 | Lake | 0.45 µm CN | Dry | 78757 | 53522 | 374 | 49 | 10 | 12 |
| JdN454 | Lake | 0.45 µm CN | Dry | 44103 | 29782 | 271 | 45 | 13 | 15 |
| Jdp451 | Lake | 12 µm CN + 0.45 µm CN | Dry | 41520 | 29177 | 225 | 28 | 2 | 10 |
| Jdp452 | Lake | 12 µm CN + 0.45 µm CN | Dry | 49275 | 33438 | 246 | 30 | 6 | 7 |
| Jdp453 | Lake | 12 µm CN + 0.45 µm CN | Dry | 45670 | 30988 | 252 | 30 | 2 | 7 |
| Jdp454 | Lake | 12 µm CN + 0.45 µm CN | Dry | 57091 | 38872 | 268 | 35 | 7 | 9 |
| JeN451 | Lake | 0.45 µm CN | EtOH | 67173 | 44218 | 267 | 34 | 8 | 10 |
| JeN452 | Lake | 0.45 µm CN | EtOH | 56811 | 38411 | 198 | 31 | 5 | 8 |
| JeN453 | Lake | 0.45 µm CN | EtOH | 62526 | 43148 | 241 | 33 | 9 | 15 |
| JeN454 | Lake | 0.45 µm CN | EtOH | 40503 | 26381 | 201 | 31 | 10 | 13 |
| Jep451 | Lake | 12 µm CN + 0.45 µm CN | EtOH | 66335 | 43696 | 199 | 23 | 4 | 12 |
| Jep452 | Lake | 12 µm CN + 0.45 µm CN | EtOH | 49543 | 31829 | 198 | 27 | 3 | 8 |
| Jep453 | Lake | 12 µm CN + 0.45 µm CN | EtOH | 69757 | 47349 | 188 | 24 | 2 | 4 |
| Jep454 | Lake | 12 µm CN + 0.45 µm CN | EtOH | 63534 | 41617 | 174 | 20 | 3 | 5 |
| JiN451 | Lake | 0.45 µm CN | Ice | 73557 | 49878 | 262 | 36 | 11 | 14 |
| JiN452 | Lake | 0.45 µm CN | Ice | 62590 | 43381 | 220 | 39 | 13 | 15 |
| JiN453 | Lake | 0.45 µm CN | Ice | 88113 | 60273 | 293 | 38 | 10 | 9 |
| JiN454 | Lake | 0.45 µm CN | Ice | 74556 | 51482 | 275 | 35 | 10 | 10 |
| JiP451 | Lake | 12 µm CN + 0.45 µm CN | Ice | 80344 | 53699 | 190 | 19 | 3 | 5 |
| JiP452 | Lake | 12 µm CN + 0.45 µm CN | Ice | 45671 | 31073 | 179 | 20 | 5 | 8 |
| JiP453 | Lake | 12 µm CN + 0.45 µm CN | Ice | 53128 | 36304 | 164 | 20 | 6 | 9 |
| JiP454 | Lake | 12 µm CN + 0.45 µm CN | Ice | 79639 | 55059 | 218 | 20 | 4 | 13 |
|  |  |  |  |  |  |  |  |  |  |
| **Supplementary Table S1** continued. | | |  |  |  |  |  |  |  |
| Sample information | | | | BE fragment | | | | | F230+BE |
| Sample | Site | Filtration | Preservation | raw reads | g-q paired reads | OTUs | Metazoa OTUs | ID Metazoa OTUs | DNA-species |
| DbC1 | River | 0.45 µm CN | Buffer | 53666 | 42102 | 1619 | 362 | 214 | 276 |
| DbC2 | River | 0.45 µm CN | Buffer | 52957 | 41355 | 1693 | 408 | 237 | 280 |
| DbC3 | River | 0.45 µm CN | Buffer | 58643 | 46030 | 1747 | 425 | 246 | 286 |
| DbC4 | River | 0.45 µm CN | Buffer | 53598 | 42039 | 1690 | 380 | 222 | 261 |
| DbP1 | River | 0.20 µm PES | Buffer | 77596 | 57750 | 1861 | 345 | 203 | 264 |
| DbP2 | River | 0.20 µm PES | Buffer | 70848 | 53338 | 1205 | 237 | 147 | 184 |
| DbP3 | River | 0.20 µm PES | Buffer | 66165 | 48453 | 1865 | 293 | 166 | 217 |
| DbP4 | River | 0.20 µm PES | Buffer | 69051 | 52154 | 1704 | 334 | 210 | 249 |
| DdC1 | River | 0.45 µm CN | Dry | 53383 | 41088 | 2065 | 388 | 218 | 270 |
| DdC2 | River | 0.45 µm CN | Dry | 45331 | 34875 | 1651 | 353 | 201 | 255 |
| DdC3 | River | 0.45 µm CN | Dry | 58837 | 45072 | 2053 | 392 | 240 | 282 |
| DdC4 | River | 0.45 µm CN | Dry | 37254 | 27890 | 1942 | 373 | 208 | 270 |
| DdP1 | River | 0.20 µm PES | Dry | 65268 | 50318 | 1650 | 351 | 206 | 251 |
| DdP2 | River | 0.20 µm PES | Dry | 64994 | 48831 | 1836 | 349 | 200 | 263 |
| DdP3 | River | 0.20 µm PES | Dry | 69739 | 52555 | 1409 | 279 | 164 | 220 |
| DdP4 | River | 0.20 µm PES | Dry | 69109 | 52079 | 1438 | 292 | 170 | 209 |
| Dec1 | River | 0.45 µm CN | EtOH | 59005 | 39174 | 2325 | 220 | 122 | 221 |
| Dec2 | River | 0.45 µm CN | EtOH | 74395 | 56948 | 1499 | 183 | 107 | 138 |
| Dec3 | River | 0.45 µm CN | EtOH | 48064 | 30891 | 1746 | 204 | 108 | 204 |
| Dec4 | River | 0.45 µm CN | EtOH | 77100 | 48242 | 1530 | 164 | 81 | 181 |
| DeP1 | River | 0.20 µm PES | EtOH | 63365 | 43807 | 1858 | 195 | 115 | 212 |
| DeP2 | River | 0.20 µm PES | EtOH | 54284 | 35632 | 1950 | 185 | 107 | 187 |
| DeP3 | River | 0.20 µm PES | EtOH | 52463 | 34089 | 1624 | 178 | 112 | 177 |
| DeP4 | River | 0.20 µm PES | EtOH | 46389 | 31727 | 1426 | 126 | 73 | 137 |
| Dic1 | River | 0.45 µm CN | Ice | 49888 | 39719 | 1273 | 347 | 223 | 261 |
| Dic2 | River | 0.45 µm CN | Ice | 49841 | 39056 | 1179 | 338 | 218 | 288 |
| Dic3 | River | 0.45 µm CN | Ice | 49453 | 37903 | 1485 | 409 | 242 | 305 |
| Dic4 | River | 0.45 µm CN | Ice | 57596 | 43030 | 1554 | 436 | 263 | 316 |
| DiP1 | River | 0.20 µm PES | Ice | 64009 | 49050 | 1321 | 316 | 197 | 230 |
| DiP2 | River | 0.20 µm PES | Ice | 65074 | 49286 | 1121 | 274 | 174 | 227 |
| DiP3 | River | 0.20 µm PES | Ice | 69038 | 51582 | 1087 | 254 | 156 | 215 |
| DiP4 | River | 0.20 µm PES | Ice | 102887 | 77477 | 1394 | 306 | 177 | 233 |
|  |  |  |  |  |  |  |  |  |  |
| **Supplementary Table S1** continued. | | |  |  |  |  |  |  |  |
| Sample information | | | | F230 fragment | | | | |  |
| Sample | Site | Filtration | Preservation | raw reads | g-q paired reads | OTUs | Metazoa OTUs | ID Metazoa OTUs |  |
| Jbene | Lake extr. neg. | - | - | 659 | 325 | 16 | 3 | 1 |  |
| JdPene | Lake extr. neg. | - | - | 7366 | 5800 | 15 | 10 | 10 |  |
| Jeene | Lake extr. neg. | - | - | 437 | 68 | 13 | 10 | 10 |  |
| Jiene | Lake extr. neg. | - | - | 3785 | 2703 | 15 | 9 | 9 |  |
| DbCene | River extr. neg. | - | - | 1456 | 637 | 26 | 15 | 14 |  |
| DbPene | River extr. neg. | - | - | 531 | 377 | 7 | 6 | 6 |  |
| DdCene | River extr. neg. | - | - | 714 | 524 | 10 | 10 | 9 |  |
| DdPene | River extr. neg. | - | - | 202 | 119 | 5 | 4 | 4 |  |
| DeCene | River extr. neg. | - | - | 2032 | 1507 | 7 | 6 | 6 |  |
| DiCene | River extr. neg. | - | - | 2147 | 1676 | 8 | 4 | 4 |  |
| JbPne | Lake neg. | 0.20 µm PES | Buffer | 14802 | 9836 | 14 | 6 | 5 |  |
| JdPne | Lake neg. | 0.20 µm PES | Dry | 1163 | 692 | 20 | 15 | 15 |  |
| JePne | Lake neg. | 0.20 µm PES | EtOH | 4083 | 3076 | 13 | 10 | 10 |  |
| JiPne | Lake neg. | 0.20 µm PES | Ice | 50270 | 37929 | 13 | 9 | 9 |  |
| DbCne | River neg. | 0.45 µm CN | Buffer | 7129 | 5791 | 19 | 12 | 11 |  |
| DbPne | River neg. | 0.20 µm PES | Buffer | 26751 | 21761 | 48 | 27 | 27 |  |
| DdCne | River neg. | 0.45 µm CN | Dry | 479 | 350 | 4 | 2 | 2 |  |
| DdPne | River neg. | 0.20 µm PES | Dry | 10899 | 8653 | 5 | 4 | 4 |  |
| DePne | River neg. | 0.20 µm PES | EtOH | 2661 | 2069 | 6 | 6 | 6 |  |
| DiCne | River neg. | 0.45 µm CN | Ice | 2741 | 2220 | 6 | 3 | 2 |  |
| DeCne | River neg. | 0.45 µm CN | EtOH | 3726 | 2825 | 8 | 5 | 5 |  |
|  |  |  |  |  |  |  |  |  |  |
| **Supplementary Table S1** continued. | | |  |  |  |  |  |  |  |
| Sample information | | | | BE fragment | | | | | F230+BE |
| Sample | Site | Filtration | Preservation | raw reads | g-q paired reads | OTUs | Metazoa OTUs | ID Metazoa OTUs | DNA-species |
|  |  |  |  |  |  |  |  |  |  |
| Jbene | Lake extr. neg. | - | - | 114 | 37 | 18 | 4 | 3 | na |
| JdPene | Lake extr. neg. | - | - | 4455 | 3683 | 16 | 6 | 6 | na |
| Jeene | Lake extr. neg. | - | - | 131 | 20 | 12 | 5 | 4 | na |
| Jiene | Lake extr. neg. | - | - | 362 | 198 | 9 | 4 | 4 | na |
| DbCene | River extr. neg. | - | - | 272 | 54 | 19 | 10 | 8 | na |
| DbPene | River extr. neg. | - | - | 71 | 57 | 1 | 0 | 0 | na |
| DdCene | River extr. neg. | - | - | 10 | 5 | 2 | 0 | 0 | na |
| DdPene | River extr. neg. | - | - | 17 | 0 | 0 | 0 | 0 | na |
| DeCene | River extr. neg. | - | - | 6 | 0 | 0 | 0 | 0 | na |
| DiCene | River extr. neg. | - | - | 9 | 4 | 3 | 1 | 1 | na |
| JbPne | Lake neg. | 0.20 µm PES | Buffer | 4850 | 3158 | 15 | 5 | 4 | na |
| JdPne | Lake neg. | 0.20 µm PES | Dry | 198 | 64 | 6 | 4 | 3 | na |
| JePne | Lake neg. | 0.20 µm PES | EtOH | 2629 | 1816 | 16 | 6 | 5 | na |
| JiPne | Lake neg. | 0.20 µm PES | Ice | 14268 | 10268 | 19 | 8 | 6 | na |
| DbCne | River neg. | 0.45 µm CN | Buffer | 325 | 241 | 13 | 6 | 6 | na |
| DbPne | River neg. | 0.20 µm PES | Buffer | 3323 | 2465 | 33 | 12 | 11 | na |
| DdCne | River neg. | 0.45 µm CN | Dry | 632 | 501 | 1 | 1 | 1 | na |
| DdPne | River neg. | 0.20 µm PES | Dry | 141 | 82 | 1 | 1 | 1 | na |
| DePne | River neg. | 0.20 µm PES | EtOH | 164 | 116 | 4 | 4 | 4 | na |
| DiCne | River neg. | 0.45 µm CN | Ice | 183 | 108 | 6 | 3 | 3 | na |
| DeCne | River neg. | 0.45 µm CN | EtOH | 63 | 25 | 2 | 1 | 1 | na |
|  |  |  |  |  |  |  |  |  |  |

Supplementary Methods. Scripts used in bioinformatic and statistical analysis

mothur > fastq.info(file=fastqFiles1.file, oligos=primers.oligos, checkorient=t, fasta=f, qfile=f, format=illumina1.8+)

fastq.info(file=fastqFiles2.file, oligos=primers.oligos, checkorient=t, fasta=f, qfile=f, format=illumina1.8+)

fastq.info(file=fastqFiles3.file, oligos=primers.oligos, checkorient=t, fasta=f, qfile=f, format=illumina1.8+)

fastq.info(file=fastqFiles4.file, oligos=primers.oligos, checkorient=t, fasta=f, qfile=f, format=illumina1.8+)

fastq.info(file=fastqFiles5.file, oligos=primers.oligos, checkorient=t, fasta=f, qfile=f, format=illumina1.8+)

fastq.info(file=fastqFiles6.file, oligos=primers.oligos, checkorient=t, fasta=f, qfile=f, format=illumina1.8+)

fastq.info(file=fastqFiles7.file, oligos=primers.oligos, checkorient=t, fasta=f, qfile=f, format=illumina1.8+)

fastq.info(file=fastqFiles8.file, oligos=primers.oligos, checkorient=t, fasta=f, qfile=f, format=illumina1.8+)

fastq.info(file=fastqFiles9.file, oligos=primers.oligos, checkorient=t, fasta=f, qfile=f, format=illumina1.8+)

fastq.info(file=fastqFiles10.file, oligos=primers.oligos, checkorient=t, fasta=f, qfile=f, format=illumina1.8+)

fastq.info(file=fastqFiles11.file, oligos=primers.oligos, checkorient=t, fasta=f, qfile=f, format=illumina1.8+)

fastq.info(file=fastqFiles12.file, oligos=primers.oligos, checkorient=t, fasta=f, qfile=f, format=illumina1.8+)

fastq.info(file=fastqFiles13.file, oligos=primers.oligos, checkorient=t, fasta=f, qfile=f, format=illumina1.8+)

fastq.info(file=fastqFiles14.file, oligos=primers.oligos, checkorient=t, fasta=f, qfile=f, format=illumina1.8+)

fastq.info(file=fastqFiles15.file, oligos=primers.oligos, checkorient=t, fasta=f, qfile=f, format=illumina1.8+)

fastq.info(file=fastqFiles16.file, oligos=primers.oligos, checkorient=t, fasta=f, qfile=f, format=illumina1.8+)

fastq.info(file=fastqFiles17.file, oligos=primers.oligos, checkorient=t, fasta=f, qfile=f, format=illumina1.8+)

fastq.info(file=fastqFiles18.file, oligos=primers.oligos, checkorient=t, fasta=f, qfile=f, format=illumina1.8+)

fastq.info(file=fastqFiles19.file, oligos=primers.oligos, checkorient=t, fasta=f, qfile=f, format=illumina1.8+)

fastq.info(file=fastqFiles20.file, oligos=primers.oligos, checkorient=t, fasta=f, qfile=f, format=illumina1.8+)

fastq.info(file=fastqFiles21.file, oligos=primers.oligos, checkorient=t, fasta=f, qfile=f, format=illumina1.8+)

fastq.info(file=fastqFiles22.file, oligos=primers.oligos, checkorient=t, fasta=f, qfile=f, format=illumina1.8+)

fastq.info(file=fastqFiles23.file, oligos=primers.oligos, checkorient=t, fasta=f, qfile=f, format=illumina1.8+)

fastq.info(file=fastqFiles24.file, oligos=primers.oligos, checkorient=t, fasta=f, qfile=f, format=illumina1.8+)

fastq.info(file=fastqFiles25.file, oligos=primers.oligos, checkorient=t, fasta=f, qfile=f, format=illumina1.8+)

fastq.info(file=fastqFiles26.file, oligos=primers.oligos, checkorient=t, fasta=f, qfile=f, format=illumina1.8+)

fastq.info(file=fastqFiles27.file, oligos=primers.oligos, checkorient=t, fasta=f, qfile=f, format=illumina1.8+)

fastq.info(file=fastqFiles28.file, oligos=primers.oligos, checkorient=t, fasta=f, qfile=f, format=illumina1.8+)

fastq.info(file=fastqFiles29.file, oligos=primers.oligos, checkorient=t, fasta=f, qfile=f, format=illumina1.8+)

fastq.info(file=fastqFiles30.file, oligos=primers.oligos, checkorient=t, fasta=f, qfile=f, format=illumina1.8+)

fastq.info(file=fastqFiles31.file, oligos=primers.oligos, checkorient=t, fasta=f, qfile=f, format=illumina1.8+)

fastq.info(file=fastqFiles32.file, oligos=primers.oligos, checkorient=t, fasta=f, qfile=f, format=illumina1.8+)

fastq.info(file=fastqFiles33.file, oligos=primers.oligos, checkorient=t, fasta=f, qfile=f, format=illumina1.8+)

fastq.info(file=fastqFiles34.file, oligos=primers.oligos, checkorient=t, fasta=f, qfile=f, format=illumina1.8+)

fastq.info(file=fastqFiles35.file, oligos=primers.oligos, checkorient=t, fasta=f, qfile=f, format=illumina1.8+)

fastq.info(file=fastqFiles36.file, oligos=primers.oligos, checkorient=t, fasta=f, qfile=f, format=illumina1.8+)

fastq.info(file=fastqFiles37.file, oligos=primers.oligos, checkorient=t, fasta=f, qfile=f, format=illumina1.8+)

fastq.info(file=fastqFiles38.file, oligos=primers.oligos, checkorient=t, fasta=f, qfile=f, format=illumina1.8+)

fastq.info(file=fastqFiles39.file, oligos=primers.oligos, checkorient=t, fasta=f, qfile=f, format=illumina1.8+)

fastq.info(file=fastqFiles40.file, oligos=primers.oligos, checkorient=t, fasta=f, qfile=f, format=illumina1.8+)

fastq.info(file=fastqFiles41.file, oligos=primers.oligos, checkorient=t, fasta=f, qfile=f, format=illumina1.8+)

fastq.info(file=fastqFiles42.file, oligos=primers.oligos, checkorient=t, fasta=f, qfile=f, format=illumina1.8+)

fastq.info(file=fastqFiles43.file, oligos=primers.oligos, checkorient=t, fasta=f, qfile=f, format=illumina1.8+)

fastq.info(file=fastqFiles44.file, oligos=primers.oligos, checkorient=t, fasta=f, qfile=f, format=illumina1.8+)

fastq.info(file=fastqFiles45.file, oligos=primers.oligos, checkorient=t, fasta=f, qfile=f, format=illumina1.8+)

fastq.info(file=fastqFiles46.file, oligos=primers.oligos, checkorient=t, fasta=f, qfile=f, format=illumina1.8+)

fastq.info(file=fastqFiles47.file, oligos=primers.oligos, checkorient=t, fasta=f, qfile=f, format=illumina1.8+)

fastq.info(file=fastqFiles48.file, oligos=primers.oligos, checkorient=t, fasta=f, qfile=f, format=illumina1.8+)

fastq.info(file=fastqFiles49.file, oligos=primers.oligos, checkorient=t, fasta=f, qfile=f, format=illumina1.8+)

fastq.info(file=fastqFiles50.file, oligos=primers.oligos, checkorient=t, fasta=f, qfile=f, format=illumina1.8+)

fastq.info(file=fastqFiles51.file, oligos=primers.oligos, checkorient=t, fasta=f, qfile=f, format=illumina1.8+)

fastq.info(file=fastqFiles52.file, oligos=primers.oligos, checkorient=t, fasta=f, qfile=f, format=illumina1.8+)

fastq.info(file=fastqFiles53.file, oligos=primers.oligos, checkorient=t, fasta=f, qfile=f, format=illumina1.8+)

fastq.info(file=fastqFiles54.file, oligos=primers.oligos, checkorient=t, fasta=f, qfile=f, format=illumina1.8+)

fastq.info(file=fastqFiles55.file, oligos=primers.oligos, checkorient=t, fasta=f, qfile=f, format=illumina1.8+)

fastq.info(file=fastqFiles56.file, oligos=primers.oligos, checkorient=t, fasta=f, qfile=f, format=illumina1.8+)

fastq.info(file=fastqFiles57.file, oligos=primers.oligos, checkorient=t, fasta=f, qfile=f, format=illumina1.8+)

fastq.info(file=fastqFiles58.file, oligos=primers.oligos, checkorient=t, fasta=f, qfile=f, format=illumina1.8+)

fastq.info(file=fastqFiles59.file, oligos=primers.oligos, checkorient=t, fasta=f, qfile=f, format=illumina1.8+)

fastq.info(file=fastqFiles60.file, oligos=primers.oligos, checkorient=t, fasta=f, qfile=f, format=illumina1.8+)

fastq.info(file=fastqFiles61.file, oligos=primers.oligos, checkorient=t, fasta=f, qfile=f, format=illumina1.8+)

fastq.info(file=fastqFiles62.file, oligos=primers.oligos, checkorient=t, fasta=f, qfile=f, format=illumina1.8+)

fastq.info(file=fastqFiles63.file, oligos=primers.oligos, checkorient=t, fasta=f, qfile=f, format=illumina1.8+)

fastq.info(file=fastqFiles64.file, oligos=primers.oligos, checkorient=t, fasta=f, qfile=f, format=illumina1.8+)

fastq.info(file=fastqFiles65.file, oligos=primers.oligos, checkorient=t, fasta=f, qfile=f, format=illumina1.8+)

fastq.info(file=fastqFiles66.file, oligos=primers.oligos, checkorient=t, fasta=f, qfile=f, format=illumina1.8+)

fastq.info(file=fastqFiles67.file, oligos=primers.oligos, checkorient=t, fasta=f, qfile=f, format=illumina1.8+)

fastq.info(file=fastqFiles68.file, oligos=primers.oligos, checkorient=t, fasta=f, qfile=f, format=illumina1.8+)

fastq.info(file=fastqFiles69.file, oligos=primers.oligos, checkorient=t, fasta=f, qfile=f, format=illumina1.8+)

fastq.info(file=fastqFiles70.file, oligos=primers.oligos, checkorient=t, fasta=f, qfile=f, format=illumina1.8+)

fastq.info(file=fastqFiles71.file, oligos=primers.oligos, checkorient=t, fasta=f, qfile=f, format=illumina1.8+)

fastq.info(file=fastqFiles72.file, oligos=primers.oligos, checkorient=t, fasta=f, qfile=f, format=illumina1.8+)

fastq.info(file=fastqFiles73.file, oligos=primers.oligos, checkorient=t, fasta=f, qfile=f, format=illumina1.8+)

fastq.info(file=fastqFiles74.file, oligos=primers.oligos, checkorient=t, fasta=f, qfile=f, format=illumina1.8+)

fastq.info(file=fastqFiles75.file, oligos=primers.oligos, checkorient=t, fasta=f, qfile=f, format=illumina1.8+)

fastq.info(file=fastqFiles76.file, oligos=primers.oligos, checkorient=t, fasta=f, qfile=f, format=illumina1.8+)

fastq.info(file=fastqFiles77.file, oligos=primers.oligos, checkorient=t, fasta=f, qfile=f, format=illumina1.8+)

fastq.info(file=fastqFiles78.file, oligos=primers.oligos, checkorient=t, fasta=f, qfile=f, format=illumina1.8+)

fastq.info(file=fastqFiles79.file, oligos=primers.oligos, checkorient=t, fasta=f, qfile=f, format=illumina1.8+)

fastq.info(file=fastqFiles80.file, oligos=primers.oligos, checkorient=t, fasta=f, qfile=f, format=illumina1.8+)

fastq.info(file=fastqFiles81.file, oligos=primers.oligos, checkorient=t, fasta=f, qfile=f, format=illumina1.8+)

fastq.info(file=fastqFiles82.file, oligos=primers.oligos, checkorient=t, fasta=f, qfile=f, format=illumina1.8+)

fastq.info(file=fastqFiles83.file, oligos=primers.oligos, checkorient=t, fasta=f, qfile=f, format=illumina1.8+)

fastq.info(file=fastqFiles84.file, oligos=primers.oligos, checkorient=t, fasta=f, qfile=f, format=illumina1.8+)

fastq.info(file=fastqFiles85.file, oligos=primers.oligos, checkorient=t, fasta=f, qfile=f, format=illumina1.8+)

mothur> make.contigs(ffastq=fastqFiles84.F230_forward.fastq, rfastq=fastqFiles84.F230_reverse.fastq, trimoverlap=T)

summary.seqs(fasta=fastqFiles84.F230_forward.trim.contigs.fasta)

screen.seqs(fasta=fastqFiles84.F230_forward.trim.contigs.fasta, maxambig=0, minlength=250, maxlength=340, contigsreport=fastqFiles84.F230_forward.contigs.report, minoverlap=25)

summary.seqs(fasta=fastqFiles84.F230_forward.trim.contigs.good.fasta)

mothur> make.contigs(ffastq=fastqFiles85.F230_forward.fastq, rfastq=fastqFiles85.F230_reverse.fastq, trimoverlap=T)

make.contigs(ffastq=fastqFiles83.F230_forward.fastq, rfastq=fastqFiles83.F230_reverse.fastq, trimoverlap=T)

make.contigs(ffastq=fastqFiles82.F230_forward.fastq, rfastq=fastqFiles82.F230_reverse.fastq, trimoverlap=T)

make.contigs(ffastq=fastqFiles81.F230_forward.fastq, rfastq=fastqFiles81.F230_reverse.fastq, trimoverlap=T)

make.contigs(ffastq=fastqFiles80.F230_forward.fastq, rfastq=fastqFiles80.F230_reverse.fastq, trimoverlap=T)

make.contigs(ffastq=fastqFiles79.F230_forward.fastq, rfastq=fastqFiles79.F230_reverse.fastq, trimoverlap=T)

make.contigs(ffastq=fastqFiles78.F230_forward.fastq, rfastq=fastqFiles78.F230_reverse.fastq, trimoverlap=T)

make.contigs(ffastq=fastqFiles77.F230_forward.fastq, rfastq=fastqFiles77.F230_reverse.fastq, trimoverlap=T)

make.contigs(ffastq=fastqFiles76.F230_forward.fastq, rfastq=fastqFiles76.F230_reverse.fastq, trimoverlap=T)

make.contigs(ffastq=fastqFiles75.F230_forward.fastq, rfastq=fastqFiles75.F230_reverse.fastq, trimoverlap=T)

make.contigs(ffastq=fastqFiles74.F230_forward.fastq, rfastq=fastqFiles74.F230_reverse.fastq, trimoverlap=T)

make.contigs(ffastq=fastqFiles73.F230_forward.fastq, rfastq=fastqFiles73.F230_reverse.fastq, trimoverlap=T)

make.contigs(ffastq=fastqFiles72.F230_forward.fastq, rfastq=fastqFiles72.F230_reverse.fastq, trimoverlap=T)

make.contigs(ffastq=fastqFiles71.F230_forward.fastq, rfastq=fastqFiles71.F230_reverse.fastq, trimoverlap=T)

make.contigs(ffastq=fastqFiles70.F230_forward.fastq, rfastq=fastqFiles70.F230_reverse.fastq, trimoverlap=T)

make.contigs(ffastq=fastqFiles69.F230_forward.fastq, rfastq=fastqFiles69.F230_reverse.fastq, trimoverlap=T)

make.contigs(ffastq=fastqFiles68.F230_forward.fastq, rfastq=fastqFiles68.F230_reverse.fastq, trimoverlap=T)

make.contigs(ffastq=fastqFiles67.F230_forward.fastq, rfastq=fastqFiles67.F230_reverse.fastq, trimoverlap=T)

make.contigs(ffastq=fastqFiles66.F230_forward.fastq, rfastq=fastqFiles66.F230_reverse.fastq, trimoverlap=T)

make.contigs(ffastq=fastqFiles65.F230_forward.fastq, rfastq=fastqFiles65.F230_reverse.fastq, trimoverlap=T)

make.contigs(ffastq=fastqFiles64.F230_forward.fastq, rfastq=fastqFiles64.F230_reverse.fastq, trimoverlap=T)

make.contigs(ffastq=fastqFiles63.F230_forward.fastq, rfastq=fastqFiles63.F230_reverse.fastq, trimoverlap=T)

make.contigs(ffastq=fastqFiles62.F230_forward.fastq, rfastq=fastqFiles62.F230_reverse.fastq, trimoverlap=T)

make.contigs(ffastq=fastqFiles61.F230_forward.fastq, rfastq=fastqFiles61.F230_reverse.fastq, trimoverlap=T)

make.contigs(ffastq=fastqFiles60.F230_forward.fastq, rfastq=fastqFiles60.F230_reverse.fastq, trimoverlap=T)

make.contigs(ffastq=fastqFiles59.F230_forward.fastq, rfastq=fastqFiles59.F230_reverse.fastq, trimoverlap=T)

make.contigs(ffastq=fastqFiles58.F230_forward.fastq, rfastq=fastqFiles58.F230_reverse.fastq, trimoverlap=T)

make.contigs(ffastq=fastqFiles57.F230_forward.fastq, rfastq=fastqFiles57.F230_reverse.fastq, trimoverlap=T)

make.contigs(ffastq=fastqFiles56.F230_forward.fastq, rfastq=fastqFiles56.F230_reverse.fastq, trimoverlap=T)

make.contigs(ffastq=fastqFiles55.F230_forward.fastq, rfastq=fastqFiles55.F230_reverse.fastq, trimoverlap=T)

make.contigs(ffastq=fastqFiles54.F230_forward.fastq, rfastq=fastqFiles54.F230_reverse.fastq, trimoverlap=T)

make.contigs(ffastq=fastqFiles53.F230_forward.fastq, rfastq=fastqFiles53.F230_reverse.fastq, trimoverlap=T)

make.contigs(ffastq=fastqFiles52.F230_forward.fastq, rfastq=fastqFiles52.F230_reverse.fastq, trimoverlap=T)

make.contigs(ffastq=fastqFiles51.F230_forward.fastq, rfastq=fastqFiles51.F230_reverse.fastq, trimoverlap=T)

make.contigs(ffastq=fastqFiles50.F230_forward.fastq, rfastq=fastqFiles50.F230_reverse.fastq, trimoverlap=T)

make.contigs(ffastq=fastqFiles49.F230_forward.fastq, rfastq=fastqFiles49.F230_reverse.fastq, trimoverlap=T)

make.contigs(ffastq=fastqFiles48.F230_forward.fastq, rfastq=fastqFiles48.F230_reverse.fastq, trimoverlap=T)

make.contigs(ffastq=fastqFiles47.F230_forward.fastq, rfastq=fastqFiles47.F230_reverse.fastq, trimoverlap=T)

make.contigs(ffastq=fastqFiles46.F230_forward.fastq, rfastq=fastqFiles46.F230_reverse.fastq, trimoverlap=T)

make.contigs(ffastq=fastqFiles45.F230_forward.fastq, rfastq=fastqFiles45.F230_reverse.fastq, trimoverlap=T)

make.contigs(ffastq=fastqFiles44.F230_forward.fastq, rfastq=fastqFiles44.F230_reverse.fastq, trimoverlap=T)

make.contigs(ffastq=fastqFiles43.F230_forward.fastq, rfastq=fastqFiles43.F230_reverse.fastq, trimoverlap=T)

make.contigs(ffastq=fastqFiles42.F230_forward.fastq, rfastq=fastqFiles42.F230_reverse.fastq, trimoverlap=T)

make.contigs(ffastq=fastqFiles41.F230_forward.fastq, rfastq=fastqFiles41.F230_reverse.fastq, trimoverlap=T)

make.contigs(ffastq=fastqFiles40.F230_forward.fastq, rfastq=fastqFiles40.F230_reverse.fastq, trimoverlap=T)

make.contigs(ffastq=fastqFiles39.F230_forward.fastq, rfastq=fastqFiles39.F230_reverse.fastq, trimoverlap=T)

make.contigs(ffastq=fastqFiles38.F230_forward.fastq, rfastq=fastqFiles38.F230_reverse.fastq, trimoverlap=T)

make.contigs(ffastq=fastqFiles37.F230_forward.fastq, rfastq=fastqFiles37.F230_reverse.fastq, trimoverlap=T)

make.contigs(ffastq=fastqFiles36.F230_forward.fastq, rfastq=fastqFiles36.F230_reverse.fastq, trimoverlap=T)

make.contigs(ffastq=fastqFiles35.F230_forward.fastq, rfastq=fastqFiles35.F230_reverse.fastq, trimoverlap=T)

make.contigs(ffastq=fastqFiles34.F230_forward.fastq, rfastq=fastqFiles34.F230_reverse.fastq, trimoverlap=T)

make.contigs(ffastq=fastqFiles33.F230_forward.fastq, rfastq=fastqFiles33.F230_reverse.fastq, trimoverlap=T)

make.contigs(ffastq=fastqFiles32.F230_forward.fastq, rfastq=fastqFiles32.F230_reverse.fastq, trimoverlap=T)

make.contigs(ffastq=fastqFiles31.F230_forward.fastq, rfastq=fastqFiles31.F230_reverse.fastq, trimoverlap=T)

make.contigs(ffastq=fastqFiles30.F230_forward.fastq, rfastq=fastqFiles30.F230_reverse.fastq, trimoverlap=T)

make.contigs(ffastq=fastqFiles29.F230_forward.fastq, rfastq=fastqFiles29.F230_reverse.fastq, trimoverlap=T)

make.contigs(ffastq=fastqFiles28.F230_forward.fastq, rfastq=fastqFiles28.F230_reverse.fastq, trimoverlap=T)

make.contigs(ffastq=fastqFiles27.F230_forward.fastq, rfastq=fastqFiles27.F230_reverse.fastq, trimoverlap=T)

make.contigs(ffastq=fastqFiles26.F230_forward.fastq, rfastq=fastqFiles26.F230_reverse.fastq, trimoverlap=T)

make.contigs(ffastq=fastqFiles25.F230_forward.fastq, rfastq=fastqFiles25.F230_reverse.fastq, trimoverlap=T)

make.contigs(ffastq=fastqFiles24.F230_forward.fastq, rfastq=fastqFiles24.F230_reverse.fastq, trimoverlap=T)

make.contigs(ffastq=fastqFiles23.F230_forward.fastq, rfastq=fastqFiles23.F230_reverse.fastq, trimoverlap=T)

make.contigs(ffastq=fastqFiles22.F230_forward.fastq, rfastq=fastqFiles22.F230_reverse.fastq, trimoverlap=T)

make.contigs(ffastq=fastqFiles21.F230_forward.fastq, rfastq=fastqFiles21.F230_reverse.fastq, trimoverlap=T)

make.contigs(ffastq=fastqFiles20.F230_forward.fastq, rfastq=fastqFiles20.F230_reverse.fastq, trimoverlap=T)

make.contigs(ffastq=fastqFiles19.F230_forward.fastq, rfastq=fastqFiles19.F230_reverse.fastq, trimoverlap=T)

make.contigs(ffastq=fastqFiles18.F230_forward.fastq, rfastq=fastqFiles18.F230_reverse.fastq, trimoverlap=T)

make.contigs(ffastq=fastqFiles17.F230_forward.fastq, rfastq=fastqFiles17.F230_reverse.fastq, trimoverlap=T)

make.contigs(ffastq=fastqFiles16.F230_forward.fastq, rfastq=fastqFiles16.F230_reverse.fastq, trimoverlap=T)

make.contigs(ffastq=fastqFiles15.F230_forward.fastq, rfastq=fastqFiles15.F230_reverse.fastq, trimoverlap=T)

make.contigs(ffastq=fastqFiles14.F230_forward.fastq, rfastq=fastqFiles14.F230_reverse.fastq, trimoverlap=T)

make.contigs(ffastq=fastqFiles13.F230_forward.fastq, rfastq=fastqFiles13.F230_reverse.fastq, trimoverlap=T)

make.contigs(ffastq=fastqFiles12.F230_forward.fastq, rfastq=fastqFiles12.F230_reverse.fastq, trimoverlap=T)

make.contigs(ffastq=fastqFiles11.F230_forward.fastq, rfastq=fastqFiles11.F230_reverse.fastq, trimoverlap=T)

make.contigs(ffastq=fastqFiles10.F230_forward.fastq, rfastq=fastqFiles10.F230_reverse.fastq, trimoverlap=T)

make.contigs(ffastq=fastqFiles9.F230_forward.fastq, rfastq=fastqFiles9.F230_reverse.fastq, trimoverlap=T)

make.contigs(ffastq=fastqFiles8.F230_forward.fastq, rfastq=fastqFiles8.F230_reverse.fastq, trimoverlap=T)

make.contigs(ffastq=fastqFiles7.F230_forward.fastq, rfastq=fastqFiles7.F230_reverse.fastq, trimoverlap=T)

make.contigs(ffastq=fastqFiles6.F230_forward.fastq, rfastq=fastqFiles6.F230_reverse.fastq, trimoverlap=T)

make.contigs(ffastq=fastqFiles5.F230_forward.fastq, rfastq=fastqFiles5.F230_reverse.fastq, trimoverlap=T)

make.contigs(ffastq=fastqFiles4.F230_forward.fastq, rfastq=fastqFiles4.F230_reverse.fastq, trimoverlap=T)

make.contigs(ffastq=fastqFiles3.F230_forward.fastq, rfastq=fastqFiles3.F230_reverse.fastq, trimoverlap=T)

make.contigs(ffastq=fastqFiles2.F230_forward.fastq, rfastq=fastqFiles2.F230_reverse.fastq, trimoverlap=T)

make.contigs(ffastq=fastqFiles1.F230_forward.fastq, rfastq=fastqFiles1.F230_reverse.fastq, trimoverlap=T)

add the header row to the contigs.report-files

screen.seqs(fasta=fastqFiles85.F230_forward.trim.contigs.fasta, maxambig=0, minlength=250, maxlength=340, contigsreport=fastqFiles85.F230_forward.contigs.report, minoverlap=25)

screen.seqs(fasta=fastqFiles83.F230_forward.trim.contigs.fasta, maxambig=0, minlength=250, maxlength=340, contigsreport=fastqFiles83.F230_forward.contigs.report, minoverlap=25)

screen.seqs(fasta=fastqFiles82.F230_forward.trim.contigs.fasta, maxambig=0, minlength=250, maxlength=340, contigsreport=fastqFiles82.F230_forward.contigs.report, minoverlap=25)

screen.seqs(fasta=fastqFiles81.F230_forward.trim.contigs.fasta, maxambig=0, minlength=250, maxlength=340, contigsreport=fastqFiles81.F230_forward.contigs.report, minoverlap=25)

screen.seqs(fasta=fastqFiles80.F230_forward.trim.contigs.fasta, maxambig=0, minlength=250, maxlength=340, contigsreport=fastqFiles80.F230_forward.contigs.report, minoverlap=25)

screen.seqs(fasta=fastqFiles79.F230_forward.trim.contigs.fasta, maxambig=0, minlength=250, maxlength=340, contigsreport=fastqFiles79.F230_forward.contigs.report, minoverlap=25)

screen.seqs(fasta=fastqFiles78.F230_forward.trim.contigs.fasta, maxambig=0, minlength=250, maxlength=340, contigsreport=fastqFiles78.F230_forward.contigs.report, minoverlap=25)

screen.seqs(fasta=fastqFiles77.F230_forward.trim.contigs.fasta, maxambig=0, minlength=250, maxlength=340, contigsreport=fastqFiles77.F230_forward.contigs.report, minoverlap=25)

screen.seqs(fasta=fastqFiles76.F230_forward.trim.contigs.fasta, maxambig=0, minlength=250, maxlength=340, contigsreport=fastqFiles76.F230_forward.contigs.report, minoverlap=25)

screen.seqs(fasta=fastqFiles75.F230_forward.trim.contigs.fasta, maxambig=0, minlength=250, maxlength=340, contigsreport=fastqFiles75.F230_forward.contigs.report, minoverlap=25)

screen.seqs(fasta=fastqFiles74.F230_forward.trim.contigs.fasta, maxambig=0, minlength=250, maxlength=340, contigsreport=fastqFiles74.F230_forward.contigs.report, minoverlap=25)

screen.seqs(fasta=fastqFiles73.F230_forward.trim.contigs.fasta, maxambig=0, minlength=250, maxlength=340, contigsreport=fastqFiles73.F230_forward.contigs.report, minoverlap=25)

screen.seqs(fasta=fastqFiles72.F230_forward.trim.contigs.fasta, maxambig=0, minlength=250, maxlength=340, contigsreport=fastqFiles72.F230_forward.contigs.report, minoverlap=25)

screen.seqs(fasta=fastqFiles71.F230_forward.trim.contigs.fasta, maxambig=0, minlength=250, maxlength=340, contigsreport=fastqFiles71.F230_forward.contigs.report, minoverlap=25)

screen.seqs(fasta=fastqFiles70.F230_forward.trim.contigs.fasta, maxambig=0, minlength=250, maxlength=340, contigsreport=fastqFiles70.F230_forward.contigs.report, minoverlap=25)

screen.seqs(fasta=fastqFiles69.F230_forward.trim.contigs.fasta, maxambig=0, minlength=250, maxlength=340, contigsreport=fastqFiles69.F230_forward.contigs.report, minoverlap=25)

screen.seqs(fasta=fastqFiles68.F230_forward.trim.contigs.fasta, maxambig=0, minlength=250, maxlength=340, contigsreport=fastqFiles68.F230_forward.contigs.report, minoverlap=25)

screen.seqs(fasta=fastqFiles67.F230_forward.trim.contigs.fasta, maxambig=0, minlength=250, maxlength=340, contigsreport=fastqFiles67.F230_forward.contigs.report, minoverlap=25)

screen.seqs(fasta=fastqFiles66.F230_forward.trim.contigs.fasta, maxambig=0, minlength=250, maxlength=340, contigsreport=fastqFiles66.F230_forward.contigs.report, minoverlap=25)

screen.seqs(fasta=fastqFiles65.F230_forward.trim.contigs.fasta, maxambig=0, minlength=250, maxlength=340, contigsreport=fastqFiles65.F230_forward.contigs.report, minoverlap=25)

screen.seqs(fasta=fastqFiles64.F230_forward.trim.contigs.fasta, maxambig=0, minlength=250, maxlength=340, contigsreport=fastqFiles64.F230_forward.contigs.report, minoverlap=25)

screen.seqs(fasta=fastqFiles63.F230_forward.trim.contigs.fasta, maxambig=0, minlength=250, maxlength=340, contigsreport=fastqFiles63.F230_forward.contigs.report, minoverlap=25)

screen.seqs(fasta=fastqFiles62.F230_forward.trim.contigs.fasta, maxambig=0, minlength=250, maxlength=340, contigsreport=fastqFiles62.F230_forward.contigs.report, minoverlap=25)

screen.seqs(fasta=fastqFiles61.F230_forward.trim.contigs.fasta, maxambig=0, minlength=250, maxlength=340, contigsreport=fastqFiles61.F230_forward.contigs.report, minoverlap=25)

screen.seqs(fasta=fastqFiles60.F230_forward.trim.contigs.fasta, maxambig=0, minlength=250, maxlength=340, contigsreport=fastqFiles60.F230_forward.contigs.report, minoverlap=25)

screen.seqs(fasta=fastqFiles59.F230_forward.trim.contigs.fasta, maxambig=0, minlength=250, maxlength=340, contigsreport=fastqFiles59.F230_forward.contigs.report, minoverlap=25)

screen.seqs(fasta=fastqFiles58.F230_forward.trim.contigs.fasta, maxambig=0, minlength=250, maxlength=340, contigsreport=fastqFiles58.F230_forward.contigs.report, minoverlap=25)

screen.seqs(fasta=fastqFiles57.F230_forward.trim.contigs.fasta, maxambig=0, minlength=250, maxlength=340, contigsreport=fastqFiles57.F230_forward.contigs.report, minoverlap=25)

screen.seqs(fasta=fastqFiles56.F230_forward.trim.contigs.fasta, maxambig=0, minlength=250, maxlength=340, contigsreport=fastqFiles56.F230_forward.contigs.report, minoverlap=25)

screen.seqs(fasta=fastqFiles55.F230_forward.trim.contigs.fasta, maxambig=0, minlength=250, maxlength=340, contigsreport=fastqFiles55.F230_forward.contigs.report, minoverlap=25)

screen.seqs(fasta=fastqFiles54.F230_forward.trim.contigs.fasta, maxambig=0, minlength=250, maxlength=340, contigsreport=fastqFiles54.F230_forward.contigs.report, minoverlap=25)

screen.seqs(fasta=fastqFiles53.F230_forward.trim.contigs.fasta, maxambig=0, minlength=250, maxlength=340, contigsreport=fastqFiles53.F230_forward.contigs.report, minoverlap=25)

screen.seqs(fasta=fastqFiles52.F230_forward.trim.contigs.fasta, maxambig=0, minlength=250, maxlength=340, contigsreport=fastqFiles52.F230_forward.contigs.report, minoverlap=25)

screen.seqs(fasta=fastqFiles51.F230_forward.trim.contigs.fasta, maxambig=0, minlength=250, maxlength=340, contigsreport=fastqFiles51.F230_forward.contigs.report, minoverlap=25)

screen.seqs(fasta=fastqFiles50.F230_forward.trim.contigs.fasta, maxambig=0, minlength=250, maxlength=340, contigsreport=fastqFiles50.F230_forward.contigs.report, minoverlap=25)

screen.seqs(fasta=fastqFiles49.F230_forward.trim.contigs.fasta, maxambig=0, minlength=250, maxlength=340, contigsreport=fastqFiles49.F230_forward.contigs.report, minoverlap=25)

screen.seqs(fasta=fastqFiles48.F230_forward.trim.contigs.fasta, maxambig=0, minlength=250, maxlength=340, contigsreport=fastqFiles48.F230_forward.contigs.report, minoverlap=25)

screen.seqs(fasta=fastqFiles47.F230_forward.trim.contigs.fasta, maxambig=0, minlength=250, maxlength=340, contigsreport=fastqFiles47.F230_forward.contigs.report, minoverlap=25)

screen.seqs(fasta=fastqFiles46.F230_forward.trim.contigs.fasta, maxambig=0, minlength=250, maxlength=340, contigsreport=fastqFiles46.F230_forward.contigs.report, minoverlap=25)

screen.seqs(fasta=fastqFiles45.F230_forward.trim.contigs.fasta, maxambig=0, minlength=250, maxlength=340, contigsreport=fastqFiles45.F230_forward.contigs.report, minoverlap=25)

screen.seqs(fasta=fastqFiles44.F230_forward.trim.contigs.fasta, maxambig=0, minlength=250, maxlength=340, contigsreport=fastqFiles44.F230_forward.contigs.report, minoverlap=25)

screen.seqs(fasta=fastqFiles43.F230_forward.trim.contigs.fasta, maxambig=0, minlength=250, maxlength=340, contigsreport=fastqFiles43.F230_forward.contigs.report, minoverlap=25)

screen.seqs(fasta=fastqFiles42.F230_forward.trim.contigs.fasta, maxambig=0, minlength=250, maxlength=340, contigsreport=fastqFiles42.F230_forward.contigs.report, minoverlap=25)

screen.seqs(fasta=fastqFiles41.F230_forward.trim.contigs.fasta, maxambig=0, minlength=250, maxlength=340, contigsreport=fastqFiles41.F230_forward.contigs.report, minoverlap=25)

screen.seqs(fasta=fastqFiles40.F230_forward.trim.contigs.fasta, maxambig=0, minlength=250, maxlength=340, contigsreport=fastqFiles40.F230_forward.contigs.report, minoverlap=25)

screen.seqs(fasta=fastqFiles39.F230_forward.trim.contigs.fasta, maxambig=0, minlength=250, maxlength=340, contigsreport=fastqFiles39.F230_forward.contigs.report, minoverlap=25)

screen.seqs(fasta=fastqFiles38.F230_forward.trim.contigs.fasta, maxambig=0, minlength=250, maxlength=340, contigsreport=fastqFiles38.F230_forward.contigs.report, minoverlap=25)

screen.seqs(fasta=fastqFiles37.F230_forward.trim.contigs.fasta, maxambig=0, minlength=250, maxlength=340, contigsreport=fastqFiles37.F230_forward.contigs.report, minoverlap=25)

screen.seqs(fasta=fastqFiles36.F230_forward.trim.contigs.fasta, maxambig=0, minlength=250, maxlength=340, contigsreport=fastqFiles36.F230_forward.contigs.report, minoverlap=25)

screen.seqs(fasta=fastqFiles35.F230_forward.trim.contigs.fasta, maxambig=0, minlength=250, maxlength=340, contigsreport=fastqFiles35.F230_forward.contigs.report, minoverlap=25)

screen.seqs(fasta=fastqFiles34.F230_forward.trim.contigs.fasta, maxambig=0, minlength=250, maxlength=340, contigsreport=fastqFiles34.F230_forward.contigs.report, minoverlap=25)

screen.seqs(fasta=fastqFiles33.F230_forward.trim.contigs.fasta, maxambig=0, minlength=250, maxlength=340, contigsreport=fastqFiles33.F230_forward.contigs.report, minoverlap=25)

screen.seqs(fasta=fastqFiles32.F230_forward.trim.contigs.fasta, maxambig=0, minlength=250, maxlength=340, contigsreport=fastqFiles32.F230_forward.contigs.report, minoverlap=25)

screen.seqs(fasta=fastqFiles31.F230_forward.trim.contigs.fasta, maxambig=0, minlength=250, maxlength=340, contigsreport=fastqFiles31.F230_forward.contigs.report, minoverlap=25)

screen.seqs(fasta=fastqFiles30.F230_forward.trim.contigs.fasta, maxambig=0, minlength=250, maxlength=340, contigsreport=fastqFiles30.F230_forward.contigs.report, minoverlap=25)

screen.seqs(fasta=fastqFiles29.F230_forward.trim.contigs.fasta, maxambig=0, minlength=250, maxlength=340, contigsreport=fastqFiles29.F230_forward.contigs.report, minoverlap=25)

screen.seqs(fasta=fastqFiles28.F230_forward.trim.contigs.fasta, maxambig=0, minlength=250, maxlength=340, contigsreport=fastqFiles28.F230_forward.contigs.report, minoverlap=25)

screen.seqs(fasta=fastqFiles27.F230_forward.trim.contigs.fasta, maxambig=0, minlength=250, maxlength=340, contigsreport=fastqFiles27.F230_forward.contigs.report, minoverlap=25)

screen.seqs(fasta=fastqFiles26.F230_forward.trim.contigs.fasta, maxambig=0, minlength=250, maxlength=340, contigsreport=fastqFiles26.F230_forward.contigs.report, minoverlap=25)

screen.seqs(fasta=fastqFiles25.F230_forward.trim.contigs.fasta, maxambig=0, minlength=250, maxlength=340, contigsreport=fastqFiles25.F230_forward.contigs.report, minoverlap=25)

screen.seqs(fasta=fastqFiles24.F230_forward.trim.contigs.fasta, maxambig=0, minlength=250, maxlength=340, contigsreport=fastqFiles24.F230_forward.contigs.report, minoverlap=25)

screen.seqs(fasta=fastqFiles23.F230_forward.trim.contigs.fasta, maxambig=0, minlength=250, maxlength=340, contigsreport=fastqFiles23.F230_forward.contigs.report, minoverlap=25)

screen.seqs(fasta=fastqFiles22.F230_forward.trim.contigs.fasta, maxambig=0, minlength=250, maxlength=340, contigsreport=fastqFiles22.F230_forward.contigs.report, minoverlap=25)

screen.seqs(fasta=fastqFiles21.F230_forward.trim.contigs.fasta, maxambig=0, minlength=250, maxlength=340, contigsreport=fastqFiles21.F230_forward.contigs.report, minoverlap=25)

screen.seqs(fasta=fastqFiles20.F230_forward.trim.contigs.fasta, maxambig=0, minlength=250, maxlength=340, contigsreport=fastqFiles20.F230_forward.contigs.report, minoverlap=25)

screen.seqs(fasta=fastqFiles19.F230_forward.trim.contigs.fasta, maxambig=0, minlength=250, maxlength=340, contigsreport=fastqFiles19.F230_forward.contigs.report, minoverlap=25)

screen.seqs(fasta=fastqFiles18.F230_forward.trim.contigs.fasta, maxambig=0, minlength=250, maxlength=340, contigsreport=fastqFiles18.F230_forward.contigs.report, minoverlap=25)

screen.seqs(fasta=fastqFiles17.F230_forward.trim.contigs.fasta, maxambig=0, minlength=250, maxlength=340, contigsreport=fastqFiles17.F230_forward.contigs.report, minoverlap=25)

screen.seqs(fasta=fastqFiles16.F230_forward.trim.contigs.fasta, maxambig=0, minlength=250, maxlength=340, contigsreport=fastqFiles16.F230_forward.contigs.report, minoverlap=25)

screen.seqs(fasta=fastqFiles15.F230_forward.trim.contigs.fasta, maxambig=0, minlength=250, maxlength=340, contigsreport=fastqFiles15.F230_forward.contigs.report, minoverlap=25)

screen.seqs(fasta=fastqFiles14.F230_forward.trim.contigs.fasta, maxambig=0, minlength=250, maxlength=340, contigsreport=fastqFiles14.F230_forward.contigs.report, minoverlap=25)

screen.seqs(fasta=fastqFiles13.F230_forward.trim.contigs.fasta, maxambig=0, minlength=250, maxlength=340, contigsreport=fastqFiles13.F230_forward.contigs.report, minoverlap=25)

screen.seqs(fasta=fastqFiles12.F230_forward.trim.contigs.fasta, maxambig=0, minlength=250, maxlength=340, contigsreport=fastqFiles12.F230_forward.contigs.report, minoverlap=25)

screen.seqs(fasta=fastqFiles11.F230_forward.trim.contigs.fasta, maxambig=0, minlength=250, maxlength=340, contigsreport=fastqFiles11.F230_forward.contigs.report, minoverlap=25)

screen.seqs(fasta=fastqFiles10.F230_forward.trim.contigs.fasta, maxambig=0, minlength=250, maxlength=340, contigsreport=fastqFiles10.F230_forward.contigs.report, minoverlap=25)

screen.seqs(fasta=fastqFiles9.F230_forward.trim.contigs.fasta, maxambig=0, minlength=250, maxlength=340, contigsreport=fastqFiles9.F230_forward.contigs.report, minoverlap=25)

screen.seqs(fasta=fastqFiles8.F230_forward.trim.contigs.fasta, maxambig=0, minlength=250, maxlength=340, contigsreport=fastqFiles8.F230_forward.contigs.report, minoverlap=25)

screen.seqs(fasta=fastqFiles7.F230_forward.trim.contigs.fasta, maxambig=0, minlength=250, maxlength=340, contigsreport=fastqFiles7.F230_forward.contigs.report, minoverlap=25)

screen.seqs(fasta=fastqFiles6.F230_forward.trim.contigs.fasta, maxambig=0, minlength=250, maxlength=340, contigsreport=fastqFiles6.F230_forward.contigs.report, minoverlap=25)

screen.seqs(fasta=fastqFiles5.F230_forward.trim.contigs.fasta, maxambig=0, minlength=250, maxlength=340, contigsreport=fastqFiles5.F230_forward.contigs.report, minoverlap=25)

screen.seqs(fasta=fastqFiles4.F230_forward.trim.contigs.fasta, maxambig=0, minlength=250, maxlength=340, contigsreport=fastqFiles4.F230_forward.contigs.report, minoverlap=25)

screen.seqs(fasta=fastqFiles3.F230_forward.trim.contigs.fasta, maxambig=0, minlength=250, maxlength=340, contigsreport=fastqFiles3.F230_forward.contigs.report, minoverlap=25)

screen.seqs(fasta=fastqFiles2.F230_forward.trim.contigs.fasta, maxambig=0, minlength=250, maxlength=340, contigsreport=fastqFiles2.F230_forward.contigs.report, minoverlap=25)

screen.seqs(fasta=fastqFiles1.F230_forward.trim.contigs.fasta, maxambig=0, minlength=250, maxlength=340, contigsreport=fastqFiles1.F230_forward.contigs.report, minoverlap=25)

primers removed:

trim.seqs(fasta=fastqFiles84.F230_forward.trim.contigs.good.fasta, oligos=F230.oligos)

trim.seqs(fasta=fastqFiles85.F230_forward.trim.contigs.good.fasta, oligos=F230.oligos)

trim.seqs(fasta=fastqFiles83.F230_forward.trim.contigs.good.fasta, oligos=F230.oligos)

trim.seqs(fasta=fastqFiles82.F230_forward.trim.contigs.good.fasta, oligos=F230.oligos)

trim.seqs(fasta=fastqFiles81.F230_forward.trim.contigs.good.fasta, oligos=F230.oligos)

trim.seqs(fasta=fastqFiles80.F230_forward.trim.contigs.good.fasta, oligos=F230.oligos)

trim.seqs(fasta=fastqFiles79.F230_forward.trim.contigs.good.fasta, oligos=F230.oligos)

trim.seqs(fasta=fastqFiles78.F230_forward.trim.contigs.good.fasta, oligos=F230.oligos)

trim.seqs(fasta=fastqFiles77.F230_forward.trim.contigs.good.fasta, oligos=F230.oligos)

trim.seqs(fasta=fastqFiles76.F230_forward.trim.contigs.good.fasta, oligos=F230.oligos)

trim.seqs(fasta=fastqFiles75.F230_forward.trim.contigs.good.fasta, oligos=F230.oligos)

trim.seqs(fasta=fastqFiles74.F230_forward.trim.contigs.good.fasta, oligos=F230.oligos)

trim.seqs(fasta=fastqFiles73.F230_forward.trim.contigs.good.fasta, oligos=F230.oligos)

trim.seqs(fasta=fastqFiles72.F230_forward.trim.contigs.good.fasta, oligos=F230.oligos)

trim.seqs(fasta=fastqFiles71.F230_forward.trim.contigs.good.fasta, oligos=F230.oligos)

trim.seqs(fasta=fastqFiles70.F230_forward.trim.contigs.good.fasta, oligos=F230.oligos)

trim.seqs(fasta=fastqFiles69.F230_forward.trim.contigs.good.fasta, oligos=F230.oligos)

trim.seqs(fasta=fastqFiles68.F230_forward.trim.contigs.good.fasta, oligos=F230.oligos)

trim.seqs(fasta=fastqFiles67.F230_forward.trim.contigs.good.fasta, oligos=F230.oligos)

trim.seqs(fasta=fastqFiles66.F230_forward.trim.contigs.good.fasta, oligos=F230.oligos)

trim.seqs(fasta=fastqFiles65.F230_forward.trim.contigs.good.fasta, oligos=F230.oligos)

trim.seqs(fasta=fastqFiles64.F230_forward.trim.contigs.good.fasta, oligos=F230.oligos)

trim.seqs(fasta=fastqFiles63.F230_forward.trim.contigs.good.fasta, oligos=F230.oligos)

trim.seqs(fasta=fastqFiles62.F230_forward.trim.contigs.good.fasta, oligos=F230.oligos)

trim.seqs(fasta=fastqFiles61.F230_forward.trim.contigs.good.fasta, oligos=F230.oligos)

trim.seqs(fasta=fastqFiles60.F230_forward.trim.contigs.good.fasta, oligos=F230.oligos)

trim.seqs(fasta=fastqFiles59.F230_forward.trim.contigs.good.fasta, oligos=F230.oligos)

trim.seqs(fasta=fastqFiles58.F230_forward.trim.contigs.good.fasta, oligos=F230.oligos)

trim.seqs(fasta=fastqFiles57.F230_forward.trim.contigs.good.fasta, oligos=F230.oligos)

trim.seqs(fasta=fastqFiles56.F230_forward.trim.contigs.good.fasta, oligos=F230.oligos)

trim.seqs(fasta=fastqFiles55.F230_forward.trim.contigs.good.fasta, oligos=F230.oligos)

trim.seqs(fasta=fastqFiles54.F230_forward.trim.contigs.good.fasta, oligos=F230.oligos)

trim.seqs(fasta=fastqFiles53.F230_forward.trim.contigs.good.fasta, oligos=F230.oligos)

trim.seqs(fasta=fastqFiles52.F230_forward.trim.contigs.good.fasta, oligos=F230.oligos)

trim.seqs(fasta=fastqFiles51.F230_forward.trim.contigs.good.fasta, oligos=F230.oligos)

trim.seqs(fasta=fastqFiles50.F230_forward.trim.contigs.good.fasta, oligos=F230.oligos)

trim.seqs(fasta=fastqFiles49.F230_forward.trim.contigs.good.fasta, oligos=F230.oligos)

trim.seqs(fasta=fastqFiles48.F230_forward.trim.contigs.good.fasta, oligos=F230.oligos)

trim.seqs(fasta=fastqFiles47.F230_forward.trim.contigs.good.fasta, oligos=F230.oligos)

trim.seqs(fasta=fastqFiles46.F230_forward.trim.contigs.good.fasta, oligos=F230.oligos)

trim.seqs(fasta=fastqFiles45.F230_forward.trim.contigs.good.fasta, oligos=F230.oligos)

trim.seqs(fasta=fastqFiles44.F230_forward.trim.contigs.good.fasta, oligos=F230.oligos)

trim.seqs(fasta=fastqFiles43.F230_forward.trim.contigs.good.fasta, oligos=F230.oligos)

trim.seqs(fasta=fastqFiles42.F230_forward.trim.contigs.good.fasta, oligos=F230.oligos)

trim.seqs(fasta=fastqFiles41.F230_forward.trim.contigs.good.fasta, oligos=F230.oligos)

trim.seqs(fasta=fastqFiles40.F230_forward.trim.contigs.good.fasta, oligos=F230.oligos)

trim.seqs(fasta=fastqFiles39.F230_forward.trim.contigs.good.fasta, oligos=F230.oligos)

trim.seqs(fasta=fastqFiles38.F230_forward.trim.contigs.good.fasta, oligos=F230.oligos)

trim.seqs(fasta=fastqFiles37.F230_forward.trim.contigs.good.fasta, oligos=F230.oligos)

trim.seqs(fasta=fastqFiles36.F230_forward.trim.contigs.good.fasta, oligos=F230.oligos)

trim.seqs(fasta=fastqFiles35.F230_forward.trim.contigs.good.fasta, oligos=F230.oligos)

trim.seqs(fasta=fastqFiles34.F230_forward.trim.contigs.good.fasta, oligos=F230.oligos)

trim.seqs(fasta=fastqFiles33.F230_forward.trim.contigs.good.fasta, oligos=F230.oligos)

trim.seqs(fasta=fastqFiles32.F230_forward.trim.contigs.good.fasta, oligos=F230.oligos)

trim.seqs(fasta=fastqFiles31.F230_forward.trim.contigs.good.fasta, oligos=F230.oligos)

trim.seqs(fasta=fastqFiles30.F230_forward.trim.contigs.good.fasta, oligos=F230.oligos)

trim.seqs(fasta=fastqFiles29.F230_forward.trim.contigs.good.fasta, oligos=F230.oligos)

trim.seqs(fasta=fastqFiles28.F230_forward.trim.contigs.good.fasta, oligos=F230.oligos)

trim.seqs(fasta=fastqFiles27.F230_forward.trim.contigs.good.fasta, oligos=F230.oligos)

trim.seqs(fasta=fastqFiles26.F230_forward.trim.contigs.good.fasta, oligos=F230.oligos)

trim.seqs(fasta=fastqFiles25.F230_forward.trim.contigs.good.fasta, oligos=F230.oligos)

trim.seqs(fasta=fastqFiles24.F230_forward.trim.contigs.good.fasta, oligos=F230.oligos)

trim.seqs(fasta=fastqFiles23.F230_forward.trim.contigs.good.fasta, oligos=F230.oligos)

trim.seqs(fasta=fastqFiles22.F230_forward.trim.contigs.good.fasta, oligos=F230.oligos)

trim.seqs(fasta=fastqFiles21.F230_forward.trim.contigs.good.fasta, oligos=F230.oligos)

trim.seqs(fasta=fastqFiles20.F230_forward.trim.contigs.good.fasta, oligos=F230.oligos)

trim.seqs(fasta=fastqFiles19.F230_forward.trim.contigs.good.fasta, oligos=F230.oligos)

trim.seqs(fasta=fastqFiles18.F230_forward.trim.contigs.good.fasta, oligos=F230.oligos)

trim.seqs(fasta=fastqFiles17.F230_forward.trim.contigs.good.fasta, oligos=F230.oligos)

trim.seqs(fasta=fastqFiles16.F230_forward.trim.contigs.good.fasta, oligos=F230.oligos)

trim.seqs(fasta=fastqFiles15.F230_forward.trim.contigs.good.fasta, oligos=F230.oligos)

trim.seqs(fasta=fastqFiles14.F230_forward.trim.contigs.good.fasta, oligos=F230.oligos)

trim.seqs(fasta=fastqFiles13.F230_forward.trim.contigs.good.fasta, oligos=F230.oligos)

trim.seqs(fasta=fastqFiles12.F230_forward.trim.contigs.good.fasta, oligos=F230.oligos)

trim.seqs(fasta=fastqFiles11.F230_forward.trim.contigs.good.fasta, oligos=F230.oligos)

trim.seqs(fasta=fastqFiles10.F230_forward.trim.contigs.good.fasta, oligos=F230.oligos)

trim.seqs(fasta=fastqFiles9.F230_forward.trim.contigs.good.fasta, oligos=F230.oligos)

trim.seqs(fasta=fastqFiles8.F230_forward.trim.contigs.good.fasta, oligos=F230.oligos)

trim.seqs(fasta=fastqFiles7.F230_forward.trim.contigs.good.fasta, oligos=F230.oligos)

trim.seqs(fasta=fastqFiles6.F230_forward.trim.contigs.good.fasta, oligos=F230.oligos)

trim.seqs(fasta=fastqFiles5.F230_forward.trim.contigs.good.fasta, oligos=F230.oligos)

trim.seqs(fasta=fastqFiles4.F230_forward.trim.contigs.good.fasta, oligos=F230.oligos)

trim.seqs(fasta=fastqFiles3.F230_forward.trim.contigs.good.fasta, oligos=F230.oligos)

trim.seqs(fasta=fastqFiles2.F230_forward.trim.contigs.good.fasta, oligos=F230.oligos)

trim.seqs(fasta=fastqFiles1.F230_forward.trim.contigs.good.fasta, oligos=F230.oligos)

usearch:

usearch -derep_fulllength fastqFiles84.F230_forward.trim.contigs.good.trim.fasta -relabel DiP4. -fastaout fastqFiles84.F230_uniques.fasta -sizeout

usearch -derep_fulllength fastqFiles85.F230_forward.trim.contigs.good.trim.fasta -relabel DeCne. -fastaout fastqFiles85.F230_uniques.fasta -sizeout

usearch -derep_fulllength fastqFiles83.F230_forward.trim.contigs.good.trim.fasta -relabel DiP3. -fastaout fastqFiles83.F230_uniques.fasta -sizeout

usearch -derep_fulllength fastqFiles82.F230_forward.trim.contigs.good.trim.fasta -relabel DiP2. -fastaout fastqFiles82.F230_uniques.fasta -sizeout

usearch -derep_fulllength fastqFiles81.F230_forward.trim.contigs.good.trim.fasta -relabel DiP1. -fastaout fastqFiles81.F230_uniques.fasta -sizeout

usearch -derep_fulllength fastqFiles80.F230_forward.trim.contigs.good.trim.fasta -relabel DiCne. -fastaout fastqFiles80.F230_uniques.fasta -sizeout

usearch -derep_fulllength fastqFiles79.F230_forward.trim.contigs.good.trim.fasta -relabel DiCene. -fastaout fastqFiles79.F230_uniques.fasta -sizeout

usearch -derep_fulllength fastqFiles78.F230_forward.trim.contigs.good.trim.fasta -relabel Dic4. -fastaout fastqFiles78.F230_uniques.fasta -sizeout

usearch -derep_fulllength fastqFiles77.F230_forward.trim.contigs.good.trim.fasta -relabel Dic3. -fastaout fastqFiles77.F230_uniques.fasta -sizeout

usearch -derep_fulllength fastqFiles76.F230_forward.trim.contigs.good.trim.fasta -relabel Dic2. -fastaout fastqFiles76.F230_uniques.fasta -sizeout

usearch -derep_fulllength fastqFiles75.F230_forward.trim.contigs.good.trim.fasta -relabel Dic1. -fastaout fastqFiles75.F230_uniques.fasta -sizeout

usearch -derep_fulllength fastqFiles74.F230_forward.trim.contigs.good.trim.fasta -relabel DePne. -fastaout fastqFiles74.F230_uniques.fasta -sizeout

usearch -derep_fulllength fastqFiles73.F230_forward.trim.contigs.good.trim.fasta -relabel DeP4. -fastaout fastqFiles73.F230_uniques.fasta -sizeout

usearch -derep_fulllength fastqFiles72.F230_forward.trim.contigs.good.trim.fasta -relabel DeP3. -fastaout fastqFiles72.F230_uniques.fasta -sizeout

usearch -derep_fulllength fastqFiles71.F230_forward.trim.contigs.good.trim.fasta -relabel DeP2. -fastaout fastqFiles71.F230_uniques.fasta -sizeout

usearch -derep_fulllength fastqFiles70.F230_forward.trim.contigs.good.trim.fasta -relabel DeP1. -fastaout fastqFiles70.F230_uniques.fasta -sizeout

usearch -derep_fulllength fastqFiles69.F230_forward.trim.contigs.good.trim.fasta -relabel DeCene. -fastaout fastqFiles69.F230_uniques.fasta -sizeout

usearch -derep_fulllength fastqFiles68.F230_forward.trim.contigs.good.trim.fasta -relabel Dec4. -fastaout fastqFiles68.F230_uniques.fasta -sizeout

usearch -derep_fulllength fastqFiles67.F230_forward.trim.contigs.good.trim.fasta -relabel Dec3. -fastaout fastqFiles67.F230_uniques.fasta -sizeout

usearch -derep_fulllength fastqFiles66.F230_forward.trim.contigs.good.trim.fasta -relabel Dec2. -fastaout fastqFiles66.F230_uniques.fasta -sizeout

usearch -derep_fulllength fastqFiles65.F230_forward.trim.contigs.good.trim.fasta -relabel Dec1. -fastaout fastqFiles65.F230_uniques.fasta -sizeout

usearch -derep_fulllength fastqFiles64.F230_forward.trim.contigs.good.trim.fasta -relabel DdPne. -fastaout fastqFiles64.F230_uniques.fasta -sizeout

usearch -derep_fulllength fastqFiles63.F230_forward.trim.contigs.good.trim.fasta -relabel DdPene. -fastaout fastqFiles63.F230_uniques.fasta -sizeout

usearch -derep_fulllength fastqFiles62.F230_forward.trim.contigs.good.trim.fasta -relabel DdP4. -fastaout fastqFiles62.F230_uniques.fasta -sizeout

usearch -derep_fulllength fastqFiles61.F230_forward.trim.contigs.good.trim.fasta -relabel DdP3. -fastaout fastqFiles61.F230_uniques.fasta -sizeout

usearch -derep_fulllength fastqFiles60.F230_forward.trim.contigs.good.trim.fasta -relabel DdP2. -fastaout fastqFiles60.F230_uniques.fasta -sizeout

usearch -derep_fulllength fastqFiles59.F230_forward.trim.contigs.good.trim.fasta -relabel DdP1. -fastaout fastqFiles59.F230_uniques.fasta -sizeout

usearch -derep_fulllength fastqFiles58.F230_forward.trim.contigs.good.trim.fasta -relabel DdCne. -fastaout fastqFiles58.F230_uniques.fasta -sizeout

usearch -derep_fulllength fastqFiles57.F230_forward.trim.contigs.good.trim.fasta -relabel DdCene. -fastaout fastqFiles57.F230_uniques.fasta -sizeout

usearch -derep_fulllength fastqFiles56.F230_forward.trim.contigs.good.trim.fasta -relabel DdC4. -fastaout fastqFiles56.F230_uniques.fasta -sizeout

usearch -derep_fulllength fastqFiles55.F230_forward.trim.contigs.good.trim.fasta -relabel DdC3. -fastaout fastqFiles55.F230_uniques.fasta -sizeout

usearch -derep_fulllength fastqFiles54.F230_forward.trim.contigs.good.trim.fasta -relabel DdC2. -fastaout fastqFiles54.F230_uniques.fasta -sizeout

usearch -derep_fulllength fastqFiles53.F230_forward.trim.contigs.good.trim.fasta -relabel DdC1. -fastaout fastqFiles53.F230_uniques.fasta -sizeout

usearch -derep_fulllength fastqFiles52.F230_forward.trim.contigs.good.trim.fasta -relabel DbPne. -fastaout fastqFiles52.F230_uniques.fasta -sizeout

usearch -derep_fulllength fastqFiles51.F230_forward.trim.contigs.good.trim.fasta -relabel DbPene. -fastaout fastqFiles51.F230_uniques.fasta -sizeout

usearch -derep_fulllength fastqFiles50.F230_forward.trim.contigs.good.trim.fasta -relabel DbP4. -fastaout fastqFiles50.F230_uniques.fasta -sizeout

usearch -derep_fulllength fastqFiles49.F230_forward.trim.contigs.good.trim.fasta -relabel DbP3. -fastaout fastqFiles49.F230_uniques.fasta -sizeout

usearch -derep_fulllength fastqFiles48.F230_forward.trim.contigs.good.trim.fasta -relabel DbP2. -fastaout fastqFiles48.F230_uniques.fasta -sizeout

usearch -derep_fulllength fastqFiles47.F230_forward.trim.contigs.good.trim.fasta -relabel DbP1. -fastaout fastqFiles47.F230_uniques.fasta -sizeout

usearch -derep_fulllength fastqFiles46.F230_forward.trim.contigs.good.trim.fasta -relabel DbCne. -fastaout fastqFiles46.F230_uniques.fasta -sizeout

usearch -derep_fulllength fastqFiles45.F230_forward.trim.contigs.good.trim.fasta -relabel DbCene. -fastaout fastqFiles45.F230_uniques.fasta -sizeout

usearch -derep_fulllength fastqFiles44.F230_forward.trim.contigs.good.trim.fasta -relabel DbC4. -fastaout fastqFiles44.F230_uniques.fasta -sizeout

usearch -derep_fulllength fastqFiles43.F230_forward.trim.contigs.good.trim.fasta -relabel DbC3. -fastaout fastqFiles43.F230_uniques.fasta -sizeout

usearch -derep_fulllength fastqFiles42.F230_forward.trim.contigs.good.trim.fasta -relabel DbC2. -fastaout fastqFiles42.F230_uniques.fasta -sizeout

usearch -derep_fulllength fastqFiles41.F230_forward.trim.contigs.good.trim.fasta -relabel DbC1. -fastaout fastqFiles41.F230_uniques.fasta -sizeout

usearch -derep_fulllength fastqFiles40.F230_forward.trim.contigs.good.trim.fasta -relabel JiPne. -fastaout fastqFiles40.F230_uniques.fasta -sizeout

usearch -derep_fulllength fastqFiles39.F230_forward.trim.contigs.good.trim.fasta -relabel Jiene. -fastaout fastqFiles39.F230_uniques.fasta -sizeout

usearch -derep_fulllength fastqFiles38.F230_forward.trim.contigs.good.trim.fasta -relabel JePne. -fastaout fastqFiles38.F230_uniques.fasta -sizeout

usearch -derep_fulllength fastqFiles37.F230_forward.trim.contigs.good.trim.fasta -relabel Jeene. -fastaout fastqFiles37.F230_uniques.fasta -sizeout

usearch -derep_fulllength fastqFiles36.F230_forward.trim.contigs.good.trim.fasta -relabel JdPne. -fastaout fastqFiles36.F230_uniques.fasta -sizeout

usearch -derep_fulllength fastqFiles35.F230_forward.trim.contigs.good.trim.fasta -relabel JdPene. -fastaout fastqFiles35.F230_uniques.fasta -sizeout

usearch -derep_fulllength fastqFiles34.F230_forward.trim.contigs.good.trim.fasta -relabel JiP454. -fastaout fastqFiles34.F230_uniques.fasta -sizeout

usearch -derep_fulllength fastqFiles33.F230_forward.trim.contigs.good.trim.fasta -relabel JiP453. -fastaout fastqFiles33.F230_uniques.fasta -sizeout

usearch -derep_fulllength fastqFiles32.F230_forward.trim.contigs.good.trim.fasta -relabel JiP452. -fastaout fastqFiles32.F230_uniques.fasta -sizeout

usearch -derep_fulllength fastqFiles31.F230_forward.trim.contigs.good.trim.fasta -relabel JiP451. -fastaout fastqFiles31.F230_uniques.fasta -sizeout

usearch -derep_fulllength fastqFiles30.F230_forward.trim.contigs.good.trim.fasta -relabel JiN454. -fastaout fastqFiles30.F230_uniques.fasta -sizeout

usearch -derep_fulllength fastqFiles29.F230_forward.trim.contigs.good.trim.fasta -relabel JiN453. -fastaout fastqFiles29.F230_uniques.fasta -sizeout

usearch -derep_fulllength fastqFiles28.F230_forward.trim.contigs.good.trim.fasta -relabel JiN452. -fastaout fastqFiles28.F230_uniques.fasta -sizeout

usearch -derep_fulllength fastqFiles27.F230_forward.trim.contigs.good.trim.fasta -relabel JiN451. -fastaout fastqFiles27.F230_uniques.fasta -sizeout

usearch -derep_fulllength fastqFiles26.F230_forward.trim.contigs.good.trim.fasta -relabel Jep454. -fastaout fastqFiles26.F230_uniques.fasta -sizeout

usearch -derep_fulllength fastqFiles25.F230_forward.trim.contigs.good.trim.fasta -relabel Jep453. -fastaout fastqFiles25.F230_uniques.fasta -sizeout

usearch -derep_fulllength fastqFiles24.F230_forward.trim.contigs.good.trim.fasta -relabel Jep452. -fastaout fastqFiles24.F230_uniques.fasta -sizeout

usearch -derep_fulllength fastqFiles23.F230_forward.trim.contigs.good.trim.fasta -relabel Jep451. -fastaout fastqFiles23.F230_uniques.fasta -sizeout

usearch -derep_fulllength fastqFiles22.F230_forward.trim.contigs.good.trim.fasta -relabel JeN454. -fastaout fastqFiles22.F230_uniques.fasta -sizeout

usearch -derep_fulllength fastqFiles21.F230_forward.trim.contigs.good.trim.fasta -relabel JeN453. -fastaout fastqFiles21.F230_uniques.fasta -sizeout

usearch -derep_fulllength fastqFiles20.F230_forward.trim.contigs.good.trim.fasta -relabel JeN452. -fastaout fastqFiles20.F230_uniques.fasta -sizeout

usearch -derep_fulllength fastqFiles19.F230_forward.trim.contigs.good.trim.fasta -relabel JeN451. -fastaout fastqFiles19.F230_uniques.fasta -sizeout

usearch -derep_fulllength fastqFiles18.F230_forward.trim.contigs.good.trim.fasta -relabel Jdp454. -fastaout fastqFiles18.F230_uniques.fasta -sizeout

usearch -derep_fulllength fastqFiles17.F230_forward.trim.contigs.good.trim.fasta -relabel Jdp453. -fastaout fastqFiles17.F230_uniques.fasta -sizeout

usearch -derep_fulllength fastqFiles16.F230_forward.trim.contigs.good.trim.fasta -relabel Jdp452. -fastaout fastqFiles16.F230_uniques.fasta -sizeout

usearch -derep_fulllength fastqFiles15.F230_forward.trim.contigs.good.trim.fasta -relabel Jdp451. -fastaout fastqFiles15.F230_uniques.fasta -sizeout

usearch -derep_fulllength fastqFiles14.F230_forward.trim.contigs.good.trim.fasta -relabel JdN454. -fastaout fastqFiles14.F230_uniques.fasta -sizeout

usearch -derep_fulllength fastqFiles13.F230_forward.trim.contigs.good.trim.fasta -relabel JdN453. -fastaout fastqFiles13.F230_uniques.fasta -sizeout

usearch -derep_fulllength fastqFiles12.F230_forward.trim.contigs.good.trim.fasta -relabel JdN452. -fastaout fastqFiles12.F230_uniques.fasta -sizeout

usearch -derep_fulllength fastqFiles11.F230_forward.trim.contigs.good.trim.fasta -relabel JdN451. -fastaout fastqFiles11.F230_uniques.fasta -sizeout

usearch -derep_fulllength fastqFiles10.F230_forward.trim.contigs.good.trim.fasta -relabel JbPne. -fastaout fastqFiles10.F230_uniques.fasta -sizeout

usearch -derep_fulllength fastqFiles9.F230_forward.trim.contigs.good.trim.fasta -relabel JbP454. -fastaout fastqFiles9.F230_uniques.fasta -sizeout

usearch -derep_fulllength fastqFiles8.F230_forward.trim.contigs.good.trim.fasta -relabel JbP453. -fastaout fastqFiles8.F230_uniques.fasta -sizeout

usearch -derep_fulllength fastqFiles7.F230_forward.trim.contigs.good.trim.fasta -relabel JbP452. -fastaout fastqFiles7.F230_uniques.fasta -sizeout

usearch -derep_fulllength fastqFiles6.F230_forward.trim.contigs.good.trim.fasta -relabel JbP451. -fastaout fastqFiles6.F230_uniques.fasta -sizeout

usearch -derep_fulllength fastqFiles5.F230_forward.trim.contigs.good.trim.fasta -relabel JbN454. -fastaout fastqFiles5.F230_uniques.fasta -sizeout

usearch -derep_fulllength fastqFiles4.F230_forward.trim.contigs.good.trim.fasta -relabel JbN453. -fastaout fastqFiles4.F230_uniques.fasta -sizeout

usearch -derep_fulllength fastqFiles3.F230_forward.trim.contigs.good.trim.fasta -relabel JbN452. -fastaout fastqFiles3.F230_uniques.fasta -sizeout

usearch -derep_fulllength fastqFiles2.F230_forward.trim.contigs.good.trim.fasta -relabel JbN451. -fastaout fastqFiles2.F230_uniques.fasta -sizeout

usearch -derep_fulllength fastqFiles1.F230_forward.trim.contigs.good.trim.fasta -relabel Jbene. -fastaout fastqFiles1.F230_uniques.fasta -sizeout

usearch -cluster_otus fastqFiles84.F230_uniques.fasta -sizein -minsize 1 -otu_radius_pct 2.0 -sizeout -otus fastqFiles84.F230_otus.fa

usearch -cluster_otus fastqFiles85.F230_uniques.fasta -sizein -minsize 1 -otu_radius_pct 2.0 -sizeout -otus fastqFiles85.F230_otus.fa

usearch -cluster_otus fastqFiles83.F230_uniques.fasta -sizein -minsize 1 -otu_radius_pct 2.0 -sizeout -otus fastqFiles83.F230_otus.fa

usearch -cluster_otus fastqFiles82.F230_uniques.fasta -sizein -minsize 1 -otu_radius_pct 2.0 -sizeout -otus fastqFiles82.F230_otus.fa

usearch -cluster_otus fastqFiles81.F230_uniques.fasta -sizein -minsize 1 -otu_radius_pct 2.0 -sizeout -otus fastqFiles81.F230_otus.fa

usearch -cluster_otus fastqFiles80.F230_uniques.fasta -sizein -minsize 1 -otu_radius_pct 2.0 -sizeout -otus fastqFiles80.F230_otus.fa

usearch -cluster_otus fastqFiles79.F230_uniques.fasta -sizein -minsize 1 -otu_radius_pct 2.0 -sizeout -otus fastqFiles79.F230_otus.fa

usearch -cluster_otus fastqFiles78.F230_uniques.fasta -sizein -minsize 1 -otu_radius_pct 2.0 -sizeout -otus fastqFiles78.F230_otus.fa

usearch -cluster_otus fastqFiles77.F230_uniques.fasta -sizein -minsize 1 -otu_radius_pct 2.0 -sizeout -otus fastqFiles77.F230_otus.fa

usearch -cluster_otus fastqFiles76.F230_uniques.fasta -sizein -minsize 1 -otu_radius_pct 2.0 -sizeout -otus fastqFiles76.F230_otus.fa

usearch -cluster_otus fastqFiles75.F230_uniques.fasta -sizein -minsize 1 -otu_radius_pct 2.0 -sizeout -otus fastqFiles75.F230_otus.fa

usearch -cluster_otus fastqFiles74.F230_uniques.fasta -sizein -minsize 1 -otu_radius_pct 2.0 -sizeout -otus fastqFiles74.F230_otus.fa

usearch -cluster_otus fastqFiles73.F230_uniques.fasta -sizein -minsize 1 -otu_radius_pct 2.0 -sizeout -otus fastqFiles73.F230_otus.fa

usearch -cluster_otus fastqFiles72.F230_uniques.fasta -sizein -minsize 1 -otu_radius_pct 2.0 -sizeout -otus fastqFiles72.F230_otus.fa

usearch -cluster_otus fastqFiles71.F230_uniques.fasta -sizein -minsize 1 -otu_radius_pct 2.0 -sizeout -otus fastqFiles71.F230_otus.fa

usearch -cluster_otus fastqFiles70.F230_uniques.fasta -sizein -minsize 1 -otu_radius_pct 2.0 -sizeout -otus fastqFiles70.F230_otus.fa

usearch -cluster_otus fastqFiles69.F230_uniques.fasta -sizein -minsize 1 -otu_radius_pct 2.0 -sizeout -otus fastqFiles69.F230_otus.fa

usearch -cluster_otus fastqFiles68.F230_uniques.fasta -sizein -minsize 1 -otu_radius_pct 2.0 -sizeout -otus fastqFiles68.F230_otus.fa

usearch -cluster_otus fastqFiles67.F230_uniques.fasta -sizein -minsize 1 -otu_radius_pct 2.0 -sizeout -otus fastqFiles67.F230_otus.fa

usearch -cluster_otus fastqFiles66.F230_uniques.fasta -sizein -minsize 1 -otu_radius_pct 2.0 -sizeout -otus fastqFiles66.F230_otus.fa

usearch -cluster_otus fastqFiles65.F230_uniques.fasta -sizein -minsize 1 -otu_radius_pct 2.0 -sizeout -otus fastqFiles65.F230_otus.fa

usearch -cluster_otus fastqFiles64.F230_uniques.fasta -sizein -minsize 1 -otu_radius_pct 2.0 -sizeout -otus fastqFiles64.F230_otus.fa

usearch -cluster_otus fastqFiles63.F230_uniques.fasta -sizein -minsize 1 -otu_radius_pct 2.0 -sizeout -otus fastqFiles63.F230_otus.fa

usearch -cluster_otus fastqFiles62.F230_uniques.fasta -sizein -minsize 1 -otu_radius_pct 2.0 -sizeout -otus fastqFiles62.F230_otus.fa

usearch -cluster_otus fastqFiles61.F230_uniques.fasta -sizein -minsize 1 -otu_radius_pct 2.0 -sizeout -otus fastqFiles61.F230_otus.fa

usearch -cluster_otus fastqFiles60.F230_uniques.fasta -sizein -minsize 1 -otu_radius_pct 2.0 -sizeout -otus fastqFiles60.F230_otus.fa

usearch -cluster_otus fastqFiles59.F230_uniques.fasta -sizein -minsize 1 -otu_radius_pct 2.0 -sizeout -otus fastqFiles59.F230_otus.fa

usearch -cluster_otus fastqFiles58.F230_uniques.fasta -sizein -minsize 1 -otu_radius_pct 2.0 -sizeout -otus fastqFiles58.F230_otus.fa

usearch -cluster_otus fastqFiles57.F230_uniques.fasta -sizein -minsize 1 -otu_radius_pct 2.0 -sizeout -otus fastqFiles57.F230_otus.fa

usearch -cluster_otus fastqFiles56.F230_uniques.fasta -sizein -minsize 1 -otu_radius_pct 2.0 -sizeout -otus fastqFiles56.F230_otus.fa

usearch -cluster_otus fastqFiles55.F230_uniques.fasta -sizein -minsize 1 -otu_radius_pct 2.0 -sizeout -otus fastqFiles55.F230_otus.fa

usearch -cluster_otus fastqFiles54.F230_uniques.fasta -sizein -minsize 1 -otu_radius_pct 2.0 -sizeout -otus fastqFiles54.F230_otus.fa

usearch -cluster_otus fastqFiles53.F230_uniques.fasta -sizein -minsize 1 -otu_radius_pct 2.0 -sizeout -otus fastqFiles53.F230_otus.fa

usearch -cluster_otus fastqFiles52.F230_uniques.fasta -sizein -minsize 1 -otu_radius_pct 2.0 -sizeout -otus fastqFiles52.F230_otus.fa

usearch -cluster_otus fastqFiles51.F230_uniques.fasta -sizein -minsize 1 -otu_radius_pct 2.0 -sizeout -otus fastqFiles51.F230_otus.fa

usearch -cluster_otus fastqFiles50.F230_uniques.fasta -sizein -minsize 1 -otu_radius_pct 2.0 -sizeout -otus fastqFiles50.F230_otus.fa

usearch -cluster_otus fastqFiles49.F230_uniques.fasta -sizein -minsize 1 -otu_radius_pct 2.0 -sizeout -otus fastqFiles49.F230_otus.fa

usearch -cluster_otus fastqFiles48.F230_uniques.fasta -sizein -minsize 1 -otu_radius_pct 2.0 -sizeout -otus fastqFiles48.F230_otus.fa

usearch -cluster_otus fastqFiles47.F230_uniques.fasta -sizein -minsize 1 -otu_radius_pct 2.0 -sizeout -otus fastqFiles47.F230_otus.fa

usearch -cluster_otus fastqFiles46.F230_uniques.fasta -sizein -minsize 1 -otu_radius_pct 2.0 -sizeout -otus fastqFiles46.F230_otus.fa

usearch -cluster_otus fastqFiles45.F230_uniques.fasta -sizein -minsize 1 -otu_radius_pct 2.0 -sizeout -otus fastqFiles45.F230_otus.fa

usearch -cluster_otus fastqFiles44.F230_uniques.fasta -sizein -minsize 1 -otu_radius_pct 2.0 -sizeout -otus fastqFiles44.F230_otus.fa

usearch -cluster_otus fastqFiles43.F230_uniques.fasta -sizein -minsize 1 -otu_radius_pct 2.0 -sizeout -otus fastqFiles43.F230_otus.fa

usearch -cluster_otus fastqFiles42.F230_uniques.fasta -sizein -minsize 1 -otu_radius_pct 2.0 -sizeout -otus fastqFiles42.F230_otus.fa

usearch -cluster_otus fastqFiles41.F230_uniques.fasta -sizein -minsize 1 -otu_radius_pct 2.0 -sizeout -otus fastqFiles41.F230_otus.fa

usearch -cluster_otus fastqFiles40.F230_uniques.fasta -sizein -minsize 1 -otu_radius_pct 2.0 -sizeout -otus fastqFiles40.F230_otus.fa

usearch -cluster_otus fastqFiles39.F230_uniques.fasta -sizein -minsize 1 -otu_radius_pct 2.0 -sizeout -otus fastqFiles39.F230_otus.fa

usearch -cluster_otus fastqFiles38.F230_uniques.fasta -sizein -minsize 1 -otu_radius_pct 2.0 -sizeout -otus fastqFiles38.F230_otus.fa

usearch -cluster_otus fastqFiles37.F230_uniques.fasta -sizein -minsize 1 -otu_radius_pct 2.0 -sizeout -otus fastqFiles37.F230_otus.fa

usearch -cluster_otus fastqFiles36.F230_uniques.fasta -sizein -minsize 1 -otu_radius_pct 2.0 -sizeout -otus fastqFiles36.F230_otus.fa

usearch -cluster_otus fastqFiles35.F230_uniques.fasta -sizein -minsize 1 -otu_radius_pct 2.0 -sizeout -otus fastqFiles35.F230_otus.fa

usearch -cluster_otus fastqFiles34.F230_uniques.fasta -sizein -minsize 1 -otu_radius_pct 2.0 -sizeout -otus fastqFiles34.F230_otus.fa

usearch -cluster_otus fastqFiles33.F230_uniques.fasta -sizein -minsize 1 -otu_radius_pct 2.0 -sizeout -otus fastqFiles33.F230_otus.fa

usearch -cluster_otus fastqFiles32.F230_uniques.fasta -sizein -minsize 1 -otu_radius_pct 2.0 -sizeout -otus fastqFiles32.F230_otus.fa

usearch -cluster_otus fastqFiles31.F230_uniques.fasta -sizein -minsize 1 -otu_radius_pct 2.0 -sizeout -otus fastqFiles31.F230_otus.fa

usearch -cluster_otus fastqFiles30.F230_uniques.fasta -sizein -minsize 1 -otu_radius_pct 2.0 -sizeout -otus fastqFiles30.F230_otus.fa

usearch -cluster_otus fastqFiles29.F230_uniques.fasta -sizein -minsize 1 -otu_radius_pct 2.0 -sizeout -otus fastqFiles29.F230_otus.fa

usearch -cluster_otus fastqFiles28.F230_uniques.fasta -sizein -minsize 1 -otu_radius_pct 2.0 -sizeout -otus fastqFiles28.F230_otus.fa

usearch -cluster_otus fastqFiles27.F230_uniques.fasta -sizein -minsize 1 -otu_radius_pct 2.0 -sizeout -otus fastqFiles27.F230_otus.fa

usearch -cluster_otus fastqFiles26.F230_uniques.fasta -sizein -minsize 1 -otu_radius_pct 2.0 -sizeout -otus fastqFiles26.F230_otus.fa

usearch -cluster_otus fastqFiles25.F230_uniques.fasta -sizein -minsize 1 -otu_radius_pct 2.0 -sizeout -otus fastqFiles25.F230_otus.fa

usearch -cluster_otus fastqFiles24.F230_uniques.fasta -sizein -minsize 1 -otu_radius_pct 2.0 -sizeout -otus fastqFiles24.F230_otus.fa

usearch -cluster_otus fastqFiles23.F230_uniques.fasta -sizein -minsize 1 -otu_radius_pct 2.0 -sizeout -otus fastqFiles23.F230_otus.fa

usearch -cluster_otus fastqFiles22.F230_uniques.fasta -sizein -minsize 1 -otu_radius_pct 2.0 -sizeout -otus fastqFiles22.F230_otus.fa

usearch -cluster_otus fastqFiles21.F230_uniques.fasta -sizein -minsize 1 -otu_radius_pct 2.0 -sizeout -otus fastqFiles21.F230_otus.fa

usearch -cluster_otus fastqFiles20.F230_uniques.fasta -sizein -minsize 1 -otu_radius_pct 2.0 -sizeout -otus fastqFiles20.F230_otus.fa

usearch -cluster_otus fastqFiles19.F230_uniques.fasta -sizein -minsize 1 -otu_radius_pct 2.0 -sizeout -otus fastqFiles19.F230_otus.fa

usearch -cluster_otus fastqFiles18.F230_uniques.fasta -sizein -minsize 1 -otu_radius_pct 2.0 -sizeout -otus fastqFiles18.F230_otus.fa

usearch -cluster_otus fastqFiles17.F230_uniques.fasta -sizein -minsize 1 -otu_radius_pct 2.0 -sizeout -otus fastqFiles17.F230_otus.fa

usearch -cluster_otus fastqFiles16.F230_uniques.fasta -sizein -minsize 1 -otu_radius_pct 2.0 -sizeout -otus fastqFiles16.F230_otus.fa

usearch -cluster_otus fastqFiles15.F230_uniques.fasta -sizein -minsize 1 -otu_radius_pct 2.0 -sizeout -otus fastqFiles15.F230_otus.fa

usearch -cluster_otus fastqFiles14.F230_uniques.fasta -sizein -minsize 1 -otu_radius_pct 2.0 -sizeout -otus fastqFiles14.F230_otus.fa

usearch -cluster_otus fastqFiles13.F230_uniques.fasta -sizein -minsize 1 -otu_radius_pct 2.0 -sizeout -otus fastqFiles13.F230_otus.fa

usearch -cluster_otus fastqFiles12.F230_uniques.fasta -sizein -minsize 1 -otu_radius_pct 2.0 -sizeout -otus fastqFiles12.F230_otus.fa

usearch -cluster_otus fastqFiles11.F230_uniques.fasta -sizein -minsize 1 -otu_radius_pct 2.0 -sizeout -otus fastqFiles11.F230_otus.fa

usearch -cluster_otus fastqFiles10.F230_uniques.fasta -sizein -minsize 1 -otu_radius_pct 2.0 -sizeout -otus fastqFiles10.F230_otus.fa

usearch -cluster_otus fastqFiles9.F230_uniques.fasta -sizein -minsize 1 -otu_radius_pct 2.0 -sizeout -otus fastqFiles9.F230_otus.fa

usearch -cluster_otus fastqFiles8.F230_uniques.fasta -sizein -minsize 1 -otu_radius_pct 2.0 -sizeout -otus fastqFiles8.F230_otus.fa

usearch -cluster_otus fastqFiles7.F230_uniques.fasta -sizein -minsize 1 -otu_radius_pct 2.0 -sizeout -otus fastqFiles7.F230_otus.fa

usearch -cluster_otus fastqFiles6.F230_uniques.fasta -sizein -minsize 1 -otu_radius_pct 2.0 -sizeout -otus fastqFiles6.F230_otus.fa

usearch -cluster_otus fastqFiles5.F230_uniques.fasta -sizein -minsize 1 -otu_radius_pct 2.0 -sizeout -otus fastqFiles5.F230_otus.fa

usearch -cluster_otus fastqFiles4.F230_uniques.fasta -sizein -minsize 1 -otu_radius_pct 2.0 -sizeout -otus fastqFiles4.F230_otus.fa

usearch -cluster_otus fastqFiles3.F230_uniques.fasta -sizein -minsize 1 -otu_radius_pct 2.0 -sizeout -otus fastqFiles3.F230_otus.fa

usearch -cluster_otus fastqFiles2.F230_uniques.fasta -sizein -minsize 1 -otu_radius_pct 2.0 -sizeout -otus fastqFiles2.F230_otus.fa

usearch -cluster_otus fastqFiles1.F230_uniques.fasta -sizein -minsize 1 -otu_radius_pct 2.0 -sizeout -otus fastqFiles1.F230_otus.fa

mothur> merge.files(input=fastqFiles1.F230_otus.fa-fastqFiles2.F230_otus.fa-fastqFiles3.F230_otus.fa-fastqFiles4.F230_otus.fa-fastqFiles5.F230_otus.fa-fastqFiles6.F230_otus.fa-fastqFiles7.F230_otus.fa-fastqFiles8.F230_otus.fa-fastqFiles9.F230_otus.fa-fastqFiles10.F230_otus.fa-fastqFiles11.F230_otus.fa-fastqFiles12.F230_otus.fa-fastqFiles13.F230_otus.fa-fastqFiles14.F230_otus.fa-fastqFiles15.F230_otus.fa-fastqFiles16.F230_otus.fa-fastqFiles17.F230_otus.fa-fastqFiles18.F230_otus.fa-fastqFiles19.F230_otus.fa-fastqFiles20.F230_otus.fa-fastqFiles21.F230_otus.fa-fastqFiles22.F230_otus.fa-fastqFiles23.F230_otus.fa-fastqFiles24.F230_otus.fa-fastqFiles25.F230_otus.fa-fastqFiles26.F230_otus.fa-fastqFiles27.F230_otus.fa-fastqFiles28.F230_otus.fa-fastqFiles29.F230_otus.fa-fastqFiles30.F230_otus.fa-fastqFiles31.F230_otus.fa-fastqFiles32.F230_otus.fa-fastqFiles33.F230_otus.fa-fastqFiles34.F230_otus.fa-fastqFiles35.F230_otus.fa-fastqFiles36.F230_otus.fa-fastqFiles37.F230_otus.fa-fastqFiles38.F230_otus.fa-fastqFiles39.F230_otus.fa-fastqFiles40.F230_otus.fa-fastqFiles41.F230_otus.fa-fastqFiles42.F230_otus.fa-fastqFiles43.F230_otus.fa-fastqFiles44.F230_otus.fa-fastqFiles45.F230_otus.fa-fastqFiles46.F230_otus.fa-fastqFiles47.F230_otus.fa-fastqFiles48.F230_otus.fa-fastqFiles49.F230_otus.fa-fastqFiles50.F230_otus.fa-fastqFiles51.F230_otus.fa-fastqFiles52.F230_otus.fa-fastqFiles53.F230_otus.fa-fastqFiles54.F230_otus.fa-fastqFiles55.F230_otus.fa-fastqFiles56.F230_otus.fa-fastqFiles57.F230_otus.fa-fastqFiles58.F230_otus.fa-fastqFiles59.F230_otus.fa-fastqFiles60.F230_otus.fa-fastqFiles61.F230_otus.fa-fastqFiles62.F230_otus.fa-fastqFiles63.F230_otus.fa-fastqFiles64.F230_otus.fa-fastqFiles65.F230_otus.fa-fastqFiles66.F230_otus.fa-fastqFiles67.F230_otus.fa-fastqFiles68.F230_otus.fa-fastqFiles69.F230_otus.fa-fastqFiles70.F230_otus.fa-fastqFiles71.F230_otus.fa-fastqFiles72.F230_otus.fa-fastqFiles73.F230_otus.fa-fastqFiles74.F230_otus.fa-fastqFiles75.F230_otus.fa-fastqFiles76.F230_otus.fa-fastqFiles77.F230_otus.fa-fastqFiles78.F230_otus.fa-fastqFiles79.F230_otus.fa-fastqFiles80.F230_otus.fa-fastqFiles81.F230_otus.fa-fastqFiles82.F230_otus.fa-fastqFiles83.F230_otus.fa-fastqFiles84.F230_otus.fa-fastqFiles85.F230_otus.fa, output=otusALL.fa)

usearch -sortbysize otusALL.fa -fastaout otusALL_sorted.fa

usearch -cluster_otus otusALL_sorted.fa -minsize 1 -otu_radius_pct 3.0 -sizein -otus otusALL_sorted_clustered_3.0.fa -sizeout -log cluster_otusALLclustered3.0.log

usearch -usearch_global otusALL.fa -db otusALL_sorted_clustered_3.0.fa -strand plus -id 0.97 -mothur_shared_out otutab_3.0.shared

Removed 41 OTUs based on their presence only or mainly in negative samples

blastn -db nt -query E:\EBAI\eDNA\eDNARun\otusALL_sorted_clustered_3.0.fa -evalue 1e-20 -num_descriptions 100 -num_alignments 100 -out E:\EBAI\eDNA\eDNARun\otusALL_sorted_clustered_3.0.taxonomy.txt -remote

MEGAN 6.4.19:

import otusALL_sorted_clustered_3.0.taxonomy.txt

minimum bit score 100.0 (low because we want all metazoa to be separate)

top percentage 8.0

Minimum support percentage 0.0001

minimum support 1

Total: 8777

Not assigned: 27(0.3%)

No hits 3879(44.2%)

Export readName_to_taxonPath to files otusALL_sorted_clustered.taxonomy-eukaryota.txt, otusALL_sorted_clustered.taxonomy-bacteria.txt, otusALL_sorted_clustered.taxonomy-notassigned.txt and otusALL_sorted_clustered.taxonomy-nohits.txt

Results put together with OTU-table, manually

2360 Metazoa

Since we are interested in Metazoa and monitoring them, we used this data to take out only Metazoa sequences.

mothur > get.seqs(accnos=metazoa.accnos.txt, fasta=otusALL_sorted_clustered_3.0.fa)

output = otusALL_sorted_clustered_3.0.pick.fa, only metazoa sequences

Translated to aminoacids (frame 2,invertebrate mitochondrial) using http://www.bioinformatics.org/sms2/translate.html

otusALL_sorted_clustered_3.0.pick.translated.fa

Translated aligned using MAFFT online tool and checked for inconsistences (non-COI reads)

otusALL_sorted_clustered_3.0.pick.translated.aligned.fa

641 stop codons

we are looking for invertebrates, so I removed all seqs (149) that had stop codons in invertebrate code.

otusALL_sorted_clustered_3.0.pick.translated.aligned.stopsremoved.fa

otusALL_sorted_clustered_3.0.pick.translated.aligned.stopsremoved.removed.fa (149 seqs)

good qualitys in otusALL_sorted_clustered_3.0.pick.translated.aligned.stopsremoved.fa checked for non-COIs, those still removed.

no obvious non-CO1s left.

mothur > get.seqs(accnos=stops.accnos.txt, fasta=otusALL_sorted_clustered_3.0.pick.fa)

output = otusALL_sorted_clustered_3.0.pick.pick.fa, stops removed file, 2211 seqs left.

Metazoa blasted against BOLD v.4 Species Level Barcode Records (2,617,911 Sequences/172,374 Species/63,613 Interim Species). Put together.

No match reads blasted against BOLD v.4 all barcode records.

Normalizations

mothur > sub.sample(shared=otutab.lake.txt, size=19431)

mothur > sub.sample(shared=otutab.river.txt, size=33730)

BE fragment:

mothur> make.contigs(ffastq=fastqFiles84.BE_forward.fastq, rfastq=fastqFiles84.BE_reverse.fastq)

summary.seqs(fasta=fastqFiles84.BE_forward.trim.contigs.fasta)

screen.seqs(fasta=fastqFiles84.BE_forward.trim.contigs.fasta, maxambig=0, minlength=330, maxlength=375, contigsreport=fastqFiles84.BE_forward.contigs.report, minoverlap=25)

summary.seqs(fasta=fastqFiles84.BE_forward.trim.contigs.good.fasta)

mothur> make.contigs(ffastq=fastqFiles85.BE_forward.fastq, rfastq=fastqFiles85.BE_reverse.fastq)

make.contigs(ffastq=fastqFiles83.BE_forward.fastq, rfastq=fastqFiles83.BE_reverse.fastq)

make.contigs(ffastq=fastqFiles82.BE_forward.fastq, rfastq=fastqFiles82.BE_reverse.fastq)

make.contigs(ffastq=fastqFiles81.BE_forward.fastq, rfastq=fastqFiles81.BE_reverse.fastq)

make.contigs(ffastq=fastqFiles80.BE_forward.fastq, rfastq=fastqFiles80.BE_reverse.fastq)

make.contigs(ffastq=fastqFiles79.BE_forward.fastq, rfastq=fastqFiles79.BE_reverse.fastq)

make.contigs(ffastq=fastqFiles78.BE_forward.fastq, rfastq=fastqFiles78.BE_reverse.fastq)

make.contigs(ffastq=fastqFiles77.BE_forward.fastq, rfastq=fastqFiles77.BE_reverse.fastq)

make.contigs(ffastq=fastqFiles76.BE_forward.fastq, rfastq=fastqFiles76.BE_reverse.fastq)

make.contigs(ffastq=fastqFiles75.BE_forward.fastq, rfastq=fastqFiles75.BE_reverse.fastq)

make.contigs(ffastq=fastqFiles74.BE_forward.fastq, rfastq=fastqFiles74.BE_reverse.fastq)

make.contigs(ffastq=fastqFiles73.BE_forward.fastq, rfastq=fastqFiles73.BE_reverse.fastq)

make.contigs(ffastq=fastqFiles72.BE_forward.fastq, rfastq=fastqFiles72.BE_reverse.fastq)

make.contigs(ffastq=fastqFiles71.BE_forward.fastq, rfastq=fastqFiles71.BE_reverse.fastq)

make.contigs(ffastq=fastqFiles70.BE_forward.fastq, rfastq=fastqFiles70.BE_reverse.fastq)

make.contigs(ffastq=fastqFiles69.BE_forward.fastq, rfastq=fastqFiles69.BE_reverse.fastq)

make.contigs(ffastq=fastqFiles68.BE_forward.fastq, rfastq=fastqFiles68.BE_reverse.fastq)

make.contigs(ffastq=fastqFiles67.BE_forward.fastq, rfastq=fastqFiles67.BE_reverse.fastq)

make.contigs(ffastq=fastqFiles66.BE_forward.fastq, rfastq=fastqFiles66.BE_reverse.fastq)

make.contigs(ffastq=fastqFiles65.BE_forward.fastq, rfastq=fastqFiles65.BE_reverse.fastq)

make.contigs(ffastq=fastqFiles64.BE_forward.fastq, rfastq=fastqFiles64.BE_reverse.fastq)

make.contigs(ffastq=fastqFiles63.BE_forward.fastq, rfastq=fastqFiles63.BE_reverse.fastq)

make.contigs(ffastq=fastqFiles62.BE_forward.fastq, rfastq=fastqFiles62.BE_reverse.fastq)

make.contigs(ffastq=fastqFiles61.BE_forward.fastq, rfastq=fastqFiles61.BE_reverse.fastq)

make.contigs(ffastq=fastqFiles60.BE_forward.fastq, rfastq=fastqFiles60.BE_reverse.fastq)

make.contigs(ffastq=fastqFiles59.BE_forward.fastq, rfastq=fastqFiles59.BE_reverse.fastq)

make.contigs(ffastq=fastqFiles58.BE_forward.fastq, rfastq=fastqFiles58.BE_reverse.fastq)

make.contigs(ffastq=fastqFiles57.BE_forward.fastq, rfastq=fastqFiles57.BE_reverse.fastq)

make.contigs(ffastq=fastqFiles56.BE_forward.fastq, rfastq=fastqFiles56.BE_reverse.fastq)

make.contigs(ffastq=fastqFiles55.BE_forward.fastq, rfastq=fastqFiles55.BE_reverse.fastq)

make.contigs(ffastq=fastqFiles54.BE_forward.fastq, rfastq=fastqFiles54.BE_reverse.fastq)

make.contigs(ffastq=fastqFiles53.BE_forward.fastq, rfastq=fastqFiles53.BE_reverse.fastq)

make.contigs(ffastq=fastqFiles52.BE_forward.fastq, rfastq=fastqFiles52.BE_reverse.fastq)

make.contigs(ffastq=fastqFiles51.BE_forward.fastq, rfastq=fastqFiles51.BE_reverse.fastq)

make.contigs(ffastq=fastqFiles50.BE_forward.fastq, rfastq=fastqFiles50.BE_reverse.fastq)

make.contigs(ffastq=fastqFiles49.BE_forward.fastq, rfastq=fastqFiles49.BE_reverse.fastq)

make.contigs(ffastq=fastqFiles48.BE_forward.fastq, rfastq=fastqFiles48.BE_reverse.fastq)

make.contigs(ffastq=fastqFiles47.BE_forward.fastq, rfastq=fastqFiles47.BE_reverse.fastq)

make.contigs(ffastq=fastqFiles46.BE_forward.fastq, rfastq=fastqFiles46.BE_reverse.fastq)

make.contigs(ffastq=fastqFiles45.BE_forward.fastq, rfastq=fastqFiles45.BE_reverse.fastq)

make.contigs(ffastq=fastqFiles44.BE_forward.fastq, rfastq=fastqFiles44.BE_reverse.fastq)

make.contigs(ffastq=fastqFiles43.BE_forward.fastq, rfastq=fastqFiles43.BE_reverse.fastq)

make.contigs(ffastq=fastqFiles42.BE_forward.fastq, rfastq=fastqFiles42.BE_reverse.fastq)

make.contigs(ffastq=fastqFiles41.BE_forward.fastq, rfastq=fastqFiles41.BE_reverse.fastq)

make.contigs(ffastq=fastqFiles40.BE_forward.fastq, rfastq=fastqFiles40.BE_reverse.fastq)

make.contigs(ffastq=fastqFiles39.BE_forward.fastq, rfastq=fastqFiles39.BE_reverse.fastq)

make.contigs(ffastq=fastqFiles38.BE_forward.fastq, rfastq=fastqFiles38.BE_reverse.fastq)

make.contigs(ffastq=fastqFiles37.BE_forward.fastq, rfastq=fastqFiles37.BE_reverse.fastq)

make.contigs(ffastq=fastqFiles36.BE_forward.fastq, rfastq=fastqFiles36.BE_reverse.fastq)

make.contigs(ffastq=fastqFiles35.BE_forward.fastq, rfastq=fastqFiles35.BE_reverse.fastq)

make.contigs(ffastq=fastqFiles34.BE_forward.fastq, rfastq=fastqFiles34.BE_reverse.fastq)

make.contigs(ffastq=fastqFiles33.BE_forward.fastq, rfastq=fastqFiles33.BE_reverse.fastq)

make.contigs(ffastq=fastqFiles32.BE_forward.fastq, rfastq=fastqFiles32.BE_reverse.fastq)

make.contigs(ffastq=fastqFiles31.BE_forward.fastq, rfastq=fastqFiles31.BE_reverse.fastq)

make.contigs(ffastq=fastqFiles30.BE_forward.fastq, rfastq=fastqFiles30.BE_reverse.fastq)

make.contigs(ffastq=fastqFiles29.BE_forward.fastq, rfastq=fastqFiles29.BE_reverse.fastq)

make.contigs(ffastq=fastqFiles28.BE_forward.fastq, rfastq=fastqFiles28.BE_reverse.fastq)

make.contigs(ffastq=fastqFiles27.BE_forward.fastq, rfastq=fastqFiles27.BE_reverse.fastq)

make.contigs(ffastq=fastqFiles26.BE_forward.fastq, rfastq=fastqFiles26.BE_reverse.fastq)

make.contigs(ffastq=fastqFiles25.BE_forward.fastq, rfastq=fastqFiles25.BE_reverse.fastq)

make.contigs(ffastq=fastqFiles24.BE_forward.fastq, rfastq=fastqFiles24.BE_reverse.fastq)

make.contigs(ffastq=fastqFiles23.BE_forward.fastq, rfastq=fastqFiles23.BE_reverse.fastq)

make.contigs(ffastq=fastqFiles22.BE_forward.fastq, rfastq=fastqFiles22.BE_reverse.fastq)

make.contigs(ffastq=fastqFiles21.BE_forward.fastq, rfastq=fastqFiles21.BE_reverse.fastq)

make.contigs(ffastq=fastqFiles20.BE_forward.fastq, rfastq=fastqFiles20.BE_reverse.fastq)

make.contigs(ffastq=fastqFiles19.BE_forward.fastq, rfastq=fastqFiles19.BE_reverse.fastq)

make.contigs(ffastq=fastqFiles18.BE_forward.fastq, rfastq=fastqFiles18.BE_reverse.fastq)

make.contigs(ffastq=fastqFiles17.BE_forward.fastq, rfastq=fastqFiles17.BE_reverse.fastq)

make.contigs(ffastq=fastqFiles16.BE_forward.fastq, rfastq=fastqFiles16.BE_reverse.fastq)

make.contigs(ffastq=fastqFiles15.BE_forward.fastq, rfastq=fastqFiles15.BE_reverse.fastq)

make.contigs(ffastq=fastqFiles14.BE_forward.fastq, rfastq=fastqFiles14.BE_reverse.fastq)

make.contigs(ffastq=fastqFiles13.BE_forward.fastq, rfastq=fastqFiles13.BE_reverse.fastq)

make.contigs(ffastq=fastqFiles12.BE_forward.fastq, rfastq=fastqFiles12.BE_reverse.fastq)

make.contigs(ffastq=fastqFiles11.BE_forward.fastq, rfastq=fastqFiles11.BE_reverse.fastq)

make.contigs(ffastq=fastqFiles10.BE_forward.fastq, rfastq=fastqFiles10.BE_reverse.fastq)

make.contigs(ffastq=fastqFiles9.BE_forward.fastq, rfastq=fastqFiles9.BE_reverse.fastq)

make.contigs(ffastq=fastqFiles8.BE_forward.fastq, rfastq=fastqFiles8.BE_reverse.fastq)

make.contigs(ffastq=fastqFiles7.BE_forward.fastq, rfastq=fastqFiles7.BE_reverse.fastq)

make.contigs(ffastq=fastqFiles6.BE_forward.fastq, rfastq=fastqFiles6.BE_reverse.fastq)

make.contigs(ffastq=fastqFiles5.BE_forward.fastq, rfastq=fastqFiles5.BE_reverse.fastq)

make.contigs(ffastq=fastqFiles4.BE_forward.fastq, rfastq=fastqFiles4.BE_reverse.fastq)

make.contigs(ffastq=fastqFiles3.BE_forward.fastq, rfastq=fastqFiles3.BE_reverse.fastq)

make.contigs(ffastq=fastqFiles2.BE_forward.fastq, rfastq=fastqFiles2.BE_reverse.fastq)

make.contigs(ffastq=fastqFiles1.BE_forward.fastq, rfastq=fastqFiles1.BE_reverse.fastq)

add the header row to the contigs.report-files

screen.seqs(fasta=fastqFiles85.BE_forward.trim.contigs.fasta, maxambig=0, minlength=330, maxlength=375, contigsreport=fastqFiles85.BE_forward.contigs.report, minoverlap=25)

screen.seqs(fasta=fastqFiles83.BE_forward.trim.contigs.fasta, maxambig=0, minlength=330, maxlength=375, contigsreport=fastqFiles83.BE_forward.contigs.report, minoverlap=25)

screen.seqs(fasta=fastqFiles82.BE_forward.trim.contigs.fasta, maxambig=0, minlength=330, maxlength=375, contigsreport=fastqFiles82.BE_forward.contigs.report, minoverlap=25)

screen.seqs(fasta=fastqFiles81.BE_forward.trim.contigs.fasta, maxambig=0, minlength=330, maxlength=375, contigsreport=fastqFiles81.BE_forward.contigs.report, minoverlap=25)

screen.seqs(fasta=fastqFiles80.BE_forward.trim.contigs.fasta, maxambig=0, minlength=330, maxlength=375, contigsreport=fastqFiles80.BE_forward.contigs.report, minoverlap=25)

screen.seqs(fasta=fastqFiles79.BE_forward.trim.contigs.fasta, maxambig=0, minlength=330, maxlength=375, contigsreport=fastqFiles79.BE_forward.contigs.report, minoverlap=25)

screen.seqs(fasta=fastqFiles78.BE_forward.trim.contigs.fasta, maxambig=0, minlength=330, maxlength=375, contigsreport=fastqFiles78.BE_forward.contigs.report, minoverlap=25)

screen.seqs(fasta=fastqFiles77.BE_forward.trim.contigs.fasta, maxambig=0, minlength=330, maxlength=375, contigsreport=fastqFiles77.BE_forward.contigs.report, minoverlap=25)

screen.seqs(fasta=fastqFiles76.BE_forward.trim.contigs.fasta, maxambig=0, minlength=330, maxlength=375, contigsreport=fastqFiles76.BE_forward.contigs.report, minoverlap=25)

screen.seqs(fasta=fastqFiles75.BE_forward.trim.contigs.fasta, maxambig=0, minlength=330, maxlength=375, contigsreport=fastqFiles75.BE_forward.contigs.report, minoverlap=25)

screen.seqs(fasta=fastqFiles74.BE_forward.trim.contigs.fasta, maxambig=0, minlength=330, maxlength=375, contigsreport=fastqFiles74.BE_forward.contigs.report, minoverlap=25)

screen.seqs(fasta=fastqFiles73.BE_forward.trim.contigs.fasta, maxambig=0, minlength=330, maxlength=375, contigsreport=fastqFiles73.BE_forward.contigs.report, minoverlap=25)

screen.seqs(fasta=fastqFiles72.BE_forward.trim.contigs.fasta, maxambig=0, minlength=330, maxlength=375, contigsreport=fastqFiles72.BE_forward.contigs.report, minoverlap=25)

screen.seqs(fasta=fastqFiles71.BE_forward.trim.contigs.fasta, maxambig=0, minlength=330, maxlength=375, contigsreport=fastqFiles71.BE_forward.contigs.report, minoverlap=25)

screen.seqs(fasta=fastqFiles70.BE_forward.trim.contigs.fasta, maxambig=0, minlength=330, maxlength=375, contigsreport=fastqFiles70.BE_forward.contigs.report, minoverlap=25)

screen.seqs(fasta=fastqFiles69.BE_forward.trim.contigs.fasta, maxambig=0, minlength=330, maxlength=375, contigsreport=fastqFiles69.BE_forward.contigs.report, minoverlap=25)

screen.seqs(fasta=fastqFiles68.BE_forward.trim.contigs.fasta, maxambig=0, minlength=330, maxlength=375, contigsreport=fastqFiles68.BE_forward.contigs.report, minoverlap=25)

screen.seqs(fasta=fastqFiles67.BE_forward.trim.contigs.fasta, maxambig=0, minlength=330, maxlength=375, contigsreport=fastqFiles67.BE_forward.contigs.report, minoverlap=25)

screen.seqs(fasta=fastqFiles66.BE_forward.trim.contigs.fasta, maxambig=0, minlength=330, maxlength=375, contigsreport=fastqFiles66.BE_forward.contigs.report, minoverlap=25)

screen.seqs(fasta=fastqFiles65.BE_forward.trim.contigs.fasta, maxambig=0, minlength=330, maxlength=375, contigsreport=fastqFiles65.BE_forward.contigs.report, minoverlap=25)

screen.seqs(fasta=fastqFiles64.BE_forward.trim.contigs.fasta, maxambig=0, minlength=330, maxlength=375, contigsreport=fastqFiles64.BE_forward.contigs.report, minoverlap=25)

screen.seqs(fasta=fastqFiles63.BE_forward.trim.contigs.fasta, maxambig=0, minlength=330, maxlength=375, contigsreport=fastqFiles63.BE_forward.contigs.report, minoverlap=25)

screen.seqs(fasta=fastqFiles62.BE_forward.trim.contigs.fasta, maxambig=0, minlength=330, maxlength=375, contigsreport=fastqFiles62.BE_forward.contigs.report, minoverlap=25)

screen.seqs(fasta=fastqFiles61.BE_forward.trim.contigs.fasta, maxambig=0, minlength=330, maxlength=375, contigsreport=fastqFiles61.BE_forward.contigs.report, minoverlap=25)

screen.seqs(fasta=fastqFiles60.BE_forward.trim.contigs.fasta, maxambig=0, minlength=330, maxlength=375, contigsreport=fastqFiles60.BE_forward.contigs.report, minoverlap=25)

screen.seqs(fasta=fastqFiles59.BE_forward.trim.contigs.fasta, maxambig=0, minlength=330, maxlength=375, contigsreport=fastqFiles59.BE_forward.contigs.report, minoverlap=25)

screen.seqs(fasta=fastqFiles58.BE_forward.trim.contigs.fasta, maxambig=0, minlength=330, maxlength=375, contigsreport=fastqFiles58.BE_forward.contigs.report, minoverlap=25)

screen.seqs(fasta=fastqFiles57.BE_forward.trim.contigs.fasta, maxambig=0, minlength=330, maxlength=375, contigsreport=fastqFiles57.BE_forward.contigs.report, minoverlap=25)

screen.seqs(fasta=fastqFiles56.BE_forward.trim.contigs.fasta, maxambig=0, minlength=330, maxlength=375, contigsreport=fastqFiles56.BE_forward.contigs.report, minoverlap=25)

screen.seqs(fasta=fastqFiles55.BE_forward.trim.contigs.fasta, maxambig=0, minlength=330, maxlength=375, contigsreport=fastqFiles55.BE_forward.contigs.report, minoverlap=25)

screen.seqs(fasta=fastqFiles54.BE_forward.trim.contigs.fasta, maxambig=0, minlength=330, maxlength=375, contigsreport=fastqFiles54.BE_forward.contigs.report, minoverlap=25)

screen.seqs(fasta=fastqFiles53.BE_forward.trim.contigs.fasta, maxambig=0, minlength=330, maxlength=375, contigsreport=fastqFiles53.BE_forward.contigs.report, minoverlap=25)

screen.seqs(fasta=fastqFiles52.BE_forward.trim.contigs.fasta, maxambig=0, minlength=330, maxlength=375, contigsreport=fastqFiles52.BE_forward.contigs.report, minoverlap=25)

screen.seqs(fasta=fastqFiles51.BE_forward.trim.contigs.fasta, maxambig=0, minlength=330, maxlength=375, contigsreport=fastqFiles51.BE_forward.contigs.report, minoverlap=25)

screen.seqs(fasta=fastqFiles50.BE_forward.trim.contigs.fasta, maxambig=0, minlength=330, maxlength=375, contigsreport=fastqFiles50.BE_forward.contigs.report, minoverlap=25)

screen.seqs(fasta=fastqFiles49.BE_forward.trim.contigs.fasta, maxambig=0, minlength=330, maxlength=375, contigsreport=fastqFiles49.BE_forward.contigs.report, minoverlap=25)

screen.seqs(fasta=fastqFiles48.BE_forward.trim.contigs.fasta, maxambig=0, minlength=330, maxlength=375, contigsreport=fastqFiles48.BE_forward.contigs.report, minoverlap=25)

screen.seqs(fasta=fastqFiles47.BE_forward.trim.contigs.fasta, maxambig=0, minlength=330, maxlength=375, contigsreport=fastqFiles47.BE_forward.contigs.report, minoverlap=25)

screen.seqs(fasta=fastqFiles46.BE_forward.trim.contigs.fasta, maxambig=0, minlength=330, maxlength=375, contigsreport=fastqFiles46.BE_forward.contigs.report, minoverlap=25)

screen.seqs(fasta=fastqFiles45.BE_forward.trim.contigs.fasta, maxambig=0, minlength=330, maxlength=375, contigsreport=fastqFiles45.BE_forward.contigs.report, minoverlap=25)

screen.seqs(fasta=fastqFiles44.BE_forward.trim.contigs.fasta, maxambig=0, minlength=330, maxlength=375, contigsreport=fastqFiles44.BE_forward.contigs.report, minoverlap=25)

screen.seqs(fasta=fastqFiles43.BE_forward.trim.contigs.fasta, maxambig=0, minlength=330, maxlength=375, contigsreport=fastqFiles43.BE_forward.contigs.report, minoverlap=25)

screen.seqs(fasta=fastqFiles42.BE_forward.trim.contigs.fasta, maxambig=0, minlength=330, maxlength=375, contigsreport=fastqFiles42.BE_forward.contigs.report, minoverlap=25)

screen.seqs(fasta=fastqFiles41.BE_forward.trim.contigs.fasta, maxambig=0, minlength=330, maxlength=375, contigsreport=fastqFiles41.BE_forward.contigs.report, minoverlap=25)

screen.seqs(fasta=fastqFiles40.BE_forward.trim.contigs.fasta, maxambig=0, minlength=330, maxlength=375, contigsreport=fastqFiles40.BE_forward.contigs.report, minoverlap=25)

screen.seqs(fasta=fastqFiles39.BE_forward.trim.contigs.fasta, maxambig=0, minlength=330, maxlength=375, contigsreport=fastqFiles39.BE_forward.contigs.report, minoverlap=25)

screen.seqs(fasta=fastqFiles38.BE_forward.trim.contigs.fasta, maxambig=0, minlength=330, maxlength=375, contigsreport=fastqFiles38.BE_forward.contigs.report, minoverlap=25)

screen.seqs(fasta=fastqFiles37.BE_forward.trim.contigs.fasta, maxambig=0, minlength=330, maxlength=375, contigsreport=fastqFiles37.BE_forward.contigs.report, minoverlap=25)

screen.seqs(fasta=fastqFiles36.BE_forward.trim.contigs.fasta, maxambig=0, minlength=330, maxlength=375, contigsreport=fastqFiles36.BE_forward.contigs.report, minoverlap=25)

screen.seqs(fasta=fastqFiles35.BE_forward.trim.contigs.fasta, maxambig=0, minlength=330, maxlength=375, contigsreport=fastqFiles35.BE_forward.contigs.report, minoverlap=25)

screen.seqs(fasta=fastqFiles34.BE_forward.trim.contigs.fasta, maxambig=0, minlength=330, maxlength=375, contigsreport=fastqFiles34.BE_forward.contigs.report, minoverlap=25)

screen.seqs(fasta=fastqFiles33.BE_forward.trim.contigs.fasta, maxambig=0, minlength=330, maxlength=375, contigsreport=fastqFiles33.BE_forward.contigs.report, minoverlap=25)

screen.seqs(fasta=fastqFiles32.BE_forward.trim.contigs.fasta, maxambig=0, minlength=330, maxlength=375, contigsreport=fastqFiles32.BE_forward.contigs.report, minoverlap=25)

screen.seqs(fasta=fastqFiles31.BE_forward.trim.contigs.fasta, maxambig=0, minlength=330, maxlength=375, contigsreport=fastqFiles31.BE_forward.contigs.report, minoverlap=25)

screen.seqs(fasta=fastqFiles30.BE_forward.trim.contigs.fasta, maxambig=0, minlength=330, maxlength=375, contigsreport=fastqFiles30.BE_forward.contigs.report, minoverlap=25)

screen.seqs(fasta=fastqFiles29.BE_forward.trim.contigs.fasta, maxambig=0, minlength=330, maxlength=375, contigsreport=fastqFiles29.BE_forward.contigs.report, minoverlap=25)

screen.seqs(fasta=fastqFiles28.BE_forward.trim.contigs.fasta, maxambig=0, minlength=330, maxlength=375, contigsreport=fastqFiles28.BE_forward.contigs.report, minoverlap=25)

screen.seqs(fasta=fastqFiles27.BE_forward.trim.contigs.fasta, maxambig=0, minlength=330, maxlength=375, contigsreport=fastqFiles27.BE_forward.contigs.report, minoverlap=25)

screen.seqs(fasta=fastqFiles26.BE_forward.trim.contigs.fasta, maxambig=0, minlength=330, maxlength=375, contigsreport=fastqFiles26.BE_forward.contigs.report, minoverlap=25)

screen.seqs(fasta=fastqFiles25.BE_forward.trim.contigs.fasta, maxambig=0, minlength=330, maxlength=375, contigsreport=fastqFiles25.BE_forward.contigs.report, minoverlap=25)

screen.seqs(fasta=fastqFiles24.BE_forward.trim.contigs.fasta, maxambig=0, minlength=330, maxlength=375, contigsreport=fastqFiles24.BE_forward.contigs.report, minoverlap=25)

screen.seqs(fasta=fastqFiles23.BE_forward.trim.contigs.fasta, maxambig=0, minlength=330, maxlength=375, contigsreport=fastqFiles23.BE_forward.contigs.report, minoverlap=25)

screen.seqs(fasta=fastqFiles22.BE_forward.trim.contigs.fasta, maxambig=0, minlength=330, maxlength=375, contigsreport=fastqFiles22.BE_forward.contigs.report, minoverlap=25)

screen.seqs(fasta=fastqFiles21.BE_forward.trim.contigs.fasta, maxambig=0, minlength=330, maxlength=375, contigsreport=fastqFiles21.BE_forward.contigs.report, minoverlap=25)

screen.seqs(fasta=fastqFiles20.BE_forward.trim.contigs.fasta, maxambig=0, minlength=330, maxlength=375, contigsreport=fastqFiles20.BE_forward.contigs.report, minoverlap=25)

screen.seqs(fasta=fastqFiles19.BE_forward.trim.contigs.fasta, maxambig=0, minlength=330, maxlength=375, contigsreport=fastqFiles19.BE_forward.contigs.report, minoverlap=25)

screen.seqs(fasta=fastqFiles18.BE_forward.trim.contigs.fasta, maxambig=0, minlength=330, maxlength=375, contigsreport=fastqFiles18.BE_forward.contigs.report, minoverlap=25)

screen.seqs(fasta=fastqFiles17.BE_forward.trim.contigs.fasta, maxambig=0, minlength=330, maxlength=375, contigsreport=fastqFiles17.BE_forward.contigs.report, minoverlap=25)

screen.seqs(fasta=fastqFiles16.BE_forward.trim.contigs.fasta, maxambig=0, minlength=330, maxlength=375, contigsreport=fastqFiles16.BE_forward.contigs.report, minoverlap=25)

screen.seqs(fasta=fastqFiles15.BE_forward.trim.contigs.fasta, maxambig=0, minlength=330, maxlength=375, contigsreport=fastqFiles15.BE_forward.contigs.report, minoverlap=25)

screen.seqs(fasta=fastqFiles14.BE_forward.trim.contigs.fasta, maxambig=0, minlength=330, maxlength=375, contigsreport=fastqFiles14.BE_forward.contigs.report, minoverlap=25)

screen.seqs(fasta=fastqFiles13.BE_forward.trim.contigs.fasta, maxambig=0, minlength=330, maxlength=375, contigsreport=fastqFiles13.BE_forward.contigs.report, minoverlap=25)

screen.seqs(fasta=fastqFiles12.BE_forward.trim.contigs.fasta, maxambig=0, minlength=330, maxlength=375, contigsreport=fastqFiles12.BE_forward.contigs.report, minoverlap=25)

screen.seqs(fasta=fastqFiles11.BE_forward.trim.contigs.fasta, maxambig=0, minlength=330, maxlength=375, contigsreport=fastqFiles11.BE_forward.contigs.report, minoverlap=25)

screen.seqs(fasta=fastqFiles10.BE_forward.trim.contigs.fasta, maxambig=0, minlength=330, maxlength=375, contigsreport=fastqFiles10.BE_forward.contigs.report, minoverlap=25)

screen.seqs(fasta=fastqFiles9.BE_forward.trim.contigs.fasta, maxambig=0, minlength=330, maxlength=375, contigsreport=fastqFiles9.BE_forward.contigs.report, minoverlap=25)

screen.seqs(fasta=fastqFiles8.BE_forward.trim.contigs.fasta, maxambig=0, minlength=330, maxlength=375, contigsreport=fastqFiles8.BE_forward.contigs.report, minoverlap=25)

screen.seqs(fasta=fastqFiles7.BE_forward.trim.contigs.fasta, maxambig=0, minlength=330, maxlength=375, contigsreport=fastqFiles7.BE_forward.contigs.report, minoverlap=25)

screen.seqs(fasta=fastqFiles6.BE_forward.trim.contigs.fasta, maxambig=0, minlength=330, maxlength=375, contigsreport=fastqFiles6.BE_forward.contigs.report, minoverlap=25)

screen.seqs(fasta=fastqFiles5.BE_forward.trim.contigs.fasta, maxambig=0, minlength=330, maxlength=375, contigsreport=fastqFiles5.BE_forward.contigs.report, minoverlap=25)

screen.seqs(fasta=fastqFiles4.BE_forward.trim.contigs.fasta, maxambig=0, minlength=330, maxlength=375, contigsreport=fastqFiles4.BE_forward.contigs.report, minoverlap=25)

screen.seqs(fasta=fastqFiles3.BE_forward.trim.contigs.fasta, maxambig=0, minlength=330, maxlength=375, contigsreport=fastqFiles3.BE_forward.contigs.report, minoverlap=25)

screen.seqs(fasta=fastqFiles2.BE_forward.trim.contigs.fasta, maxambig=0, minlength=330, maxlength=375, contigsreport=fastqFiles2.BE_forward.contigs.report, minoverlap=25)

screen.seqs(fasta=fastqFiles1.BE_forward.trim.contigs.fasta, maxambig=0, minlength=330, maxlength=375, contigsreport=fastqFiles1.BE_forward.contigs.report, minoverlap=25)

primers removed:

trim.seqs(fasta=fastqFiles84.BE_forward.trim.contigs.good.fasta, oligos=BE.oligos)

trim.seqs(fasta=fastqFiles85.BE_forward.trim.contigs.good.fasta, oligos=BE.oligos)

trim.seqs(fasta=fastqFiles83.BE_forward.trim.contigs.good.fasta, oligos=BE.oligos)

trim.seqs(fasta=fastqFiles82.BE_forward.trim.contigs.good.fasta, oligos=BE.oligos)

trim.seqs(fasta=fastqFiles81.BE_forward.trim.contigs.good.fasta, oligos=BE.oligos)

trim.seqs(fasta=fastqFiles80.BE_forward.trim.contigs.good.fasta, oligos=BE.oligos)

trim.seqs(fasta=fastqFiles79.BE_forward.trim.contigs.good.fasta, oligos=BE.oligos)

trim.seqs(fasta=fastqFiles78.BE_forward.trim.contigs.good.fasta, oligos=BE.oligos)

trim.seqs(fasta=fastqFiles77.BE_forward.trim.contigs.good.fasta, oligos=BE.oligos)

trim.seqs(fasta=fastqFiles76.BE_forward.trim.contigs.good.fasta, oligos=BE.oligos)

trim.seqs(fasta=fastqFiles75.BE_forward.trim.contigs.good.fasta, oligos=BE.oligos)

trim.seqs(fasta=fastqFiles74.BE_forward.trim.contigs.good.fasta, oligos=BE.oligos)

trim.seqs(fasta=fastqFiles73.BE_forward.trim.contigs.good.fasta, oligos=BE.oligos)

trim.seqs(fasta=fastqFiles72.BE_forward.trim.contigs.good.fasta, oligos=BE.oligos)

trim.seqs(fasta=fastqFiles71.BE_forward.trim.contigs.good.fasta, oligos=BE.oligos)

trim.seqs(fasta=fastqFiles70.BE_forward.trim.contigs.good.fasta, oligos=BE.oligos)

trim.seqs(fasta=fastqFiles68.BE_forward.trim.contigs.good.fasta, oligos=BE.oligos)

trim.seqs(fasta=fastqFiles67.BE_forward.trim.contigs.good.fasta, oligos=BE.oligos)

trim.seqs(fasta=fastqFiles66.BE_forward.trim.contigs.good.fasta, oligos=BE.oligos)

trim.seqs(fasta=fastqFiles65.BE_forward.trim.contigs.good.fasta, oligos=BE.oligos)

trim.seqs(fasta=fastqFiles64.BE_forward.trim.contigs.good.fasta, oligos=BE.oligos)

trim.seqs(fasta=fastqFiles62.BE_forward.trim.contigs.good.fasta, oligos=BE.oligos)

trim.seqs(fasta=fastqFiles61.BE_forward.trim.contigs.good.fasta, oligos=BE.oligos)

trim.seqs(fasta=fastqFiles60.BE_forward.trim.contigs.good.fasta, oligos=BE.oligos)

trim.seqs(fasta=fastqFiles59.BE_forward.trim.contigs.good.fasta, oligos=BE.oligos)

trim.seqs(fasta=fastqFiles58.BE_forward.trim.contigs.good.fasta, oligos=BE.oligos)

trim.seqs(fasta=fastqFiles57.BE_forward.trim.contigs.good.fasta, oligos=BE.oligos)

trim.seqs(fasta=fastqFiles56.BE_forward.trim.contigs.good.fasta, oligos=BE.oligos)

trim.seqs(fasta=fastqFiles55.BE_forward.trim.contigs.good.fasta, oligos=BE.oligos)

trim.seqs(fasta=fastqFiles54.BE_forward.trim.contigs.good.fasta, oligos=BE.oligos)

trim.seqs(fasta=fastqFiles53.BE_forward.trim.contigs.good.fasta, oligos=BE.oligos)

trim.seqs(fasta=fastqFiles52.BE_forward.trim.contigs.good.fasta, oligos=BE.oligos)

trim.seqs(fasta=fastqFiles51.BE_forward.trim.contigs.good.fasta, oligos=BE.oligos)

trim.seqs(fasta=fastqFiles50.BE_forward.trim.contigs.good.fasta, oligos=BE.oligos)

trim.seqs(fasta=fastqFiles49.BE_forward.trim.contigs.good.fasta, oligos=BE.oligos)

trim.seqs(fasta=fastqFiles48.BE_forward.trim.contigs.good.fasta, oligos=BE.oligos)

trim.seqs(fasta=fastqFiles47.BE_forward.trim.contigs.good.fasta, oligos=BE.oligos)

trim.seqs(fasta=fastqFiles46.BE_forward.trim.contigs.good.fasta, oligos=BE.oligos)

trim.seqs(fasta=fastqFiles45.BE_forward.trim.contigs.good.fasta, oligos=BE.oligos)

trim.seqs(fasta=fastqFiles44.BE_forward.trim.contigs.good.fasta, oligos=BE.oligos)

trim.seqs(fasta=fastqFiles43.BE_forward.trim.contigs.good.fasta, oligos=BE.oligos)

trim.seqs(fasta=fastqFiles42.BE_forward.trim.contigs.good.fasta, oligos=BE.oligos)

trim.seqs(fasta=fastqFiles41.BE_forward.trim.contigs.good.fasta, oligos=BE.oligos)

trim.seqs(fasta=fastqFiles40.BE_forward.trim.contigs.good.fasta, oligos=BE.oligos)

trim.seqs(fasta=fastqFiles39.BE_forward.trim.contigs.good.fasta, oligos=BE.oligos)

trim.seqs(fasta=fastqFiles38.BE_forward.trim.contigs.good.fasta, oligos=BE.oligos)

trim.seqs(fasta=fastqFiles37.BE_forward.trim.contigs.good.fasta, oligos=BE.oligos)

trim.seqs(fasta=fastqFiles36.BE_forward.trim.contigs.good.fasta, oligos=BE.oligos)

trim.seqs(fasta=fastqFiles35.BE_forward.trim.contigs.good.fasta, oligos=BE.oligos)

trim.seqs(fasta=fastqFiles34.BE_forward.trim.contigs.good.fasta, oligos=BE.oligos)

trim.seqs(fasta=fastqFiles33.BE_forward.trim.contigs.good.fasta, oligos=BE.oligos)

trim.seqs(fasta=fastqFiles32.BE_forward.trim.contigs.good.fasta, oligos=BE.oligos)

trim.seqs(fasta=fastqFiles31.BE_forward.trim.contigs.good.fasta, oligos=BE.oligos)

trim.seqs(fasta=fastqFiles30.BE_forward.trim.contigs.good.fasta, oligos=BE.oligos)

trim.seqs(fasta=fastqFiles29.BE_forward.trim.contigs.good.fasta, oligos=BE.oligos)

trim.seqs(fasta=fastqFiles28.BE_forward.trim.contigs.good.fasta, oligos=BE.oligos)

trim.seqs(fasta=fastqFiles27.BE_forward.trim.contigs.good.fasta, oligos=BE.oligos)

trim.seqs(fasta=fastqFiles26.BE_forward.trim.contigs.good.fasta, oligos=BE.oligos)

trim.seqs(fasta=fastqFiles25.BE_forward.trim.contigs.good.fasta, oligos=BE.oligos)

trim.seqs(fasta=fastqFiles24.BE_forward.trim.contigs.good.fasta, oligos=BE.oligos)

trim.seqs(fasta=fastqFiles23.BE_forward.trim.contigs.good.fasta, oligos=BE.oligos)

trim.seqs(fasta=fastqFiles22.BE_forward.trim.contigs.good.fasta, oligos=BE.oligos)

trim.seqs(fasta=fastqFiles21.BE_forward.trim.contigs.good.fasta, oligos=BE.oligos)

trim.seqs(fasta=fastqFiles20.BE_forward.trim.contigs.good.fasta, oligos=BE.oligos)

trim.seqs(fasta=fastqFiles19.BE_forward.trim.contigs.good.fasta, oligos=BE.oligos)

trim.seqs(fasta=fastqFiles18.BE_forward.trim.contigs.good.fasta, oligos=BE.oligos)

trim.seqs(fasta=fastqFiles17.BE_forward.trim.contigs.good.fasta, oligos=BE.oligos)

trim.seqs(fasta=fastqFiles16.BE_forward.trim.contigs.good.fasta, oligos=BE.oligos)

trim.seqs(fasta=fastqFiles15.BE_forward.trim.contigs.good.fasta, oligos=BE.oligos)

trim.seqs(fasta=fastqFiles14.BE_forward.trim.contigs.good.fasta, oligos=BE.oligos)

trim.seqs(fasta=fastqFiles13.BE_forward.trim.contigs.good.fasta, oligos=BE.oligos)

trim.seqs(fasta=fastqFiles12.BE_forward.trim.contigs.good.fasta, oligos=BE.oligos)

trim.seqs(fasta=fastqFiles11.BE_forward.trim.contigs.good.fasta, oligos=BE.oligos)

trim.seqs(fasta=fastqFiles10.BE_forward.trim.contigs.good.fasta, oligos=BE.oligos)

trim.seqs(fasta=fastqFiles9.BE_forward.trim.contigs.good.fasta, oligos=BE.oligos)

trim.seqs(fasta=fastqFiles8.BE_forward.trim.contigs.good.fasta, oligos=BE.oligos)

trim.seqs(fasta=fastqFiles7.BE_forward.trim.contigs.good.fasta, oligos=BE.oligos)

trim.seqs(fasta=fastqFiles6.BE_forward.trim.contigs.good.fasta, oligos=BE.oligos)

trim.seqs(fasta=fastqFiles5.BE_forward.trim.contigs.good.fasta, oligos=BE.oligos)

trim.seqs(fasta=fastqFiles4.BE_forward.trim.contigs.good.fasta, oligos=BE.oligos)

trim.seqs(fasta=fastqFiles3.BE_forward.trim.contigs.good.fasta, oligos=BE.oligos)

trim.seqs(fasta=fastqFiles2.BE_forward.trim.contigs.good.fasta, oligos=BE.oligos)

trim.seqs(fasta=fastqFiles1.BE_forward.trim.contigs.good.fasta, oligos=BE.oligos)

usearch:

usearch -derep_fulllength fastqFiles84.BE_forward.trim.contigs.good.trim.fasta -relabel DiP4. -fastaout fastqFiles84.BE_uniques.fasta -sizeout

usearch -derep_fulllength fastqFiles85.BE_forward.trim.contigs.good.trim.fasta -relabel DeCne. -fastaout fastqFiles85.BE_uniques.fasta -sizeout

usearch -derep_fulllength fastqFiles83.BE_forward.trim.contigs.good.trim.fasta -relabel DiP3. -fastaout fastqFiles83.BE_uniques.fasta -sizeout

usearch -derep_fulllength fastqFiles82.BE_forward.trim.contigs.good.trim.fasta -relabel DiP2. -fastaout fastqFiles82.BE_uniques.fasta -sizeout

usearch -derep_fulllength fastqFiles81.BE_forward.trim.contigs.good.trim.fasta -relabel DiP1. -fastaout fastqFiles81.BE_uniques.fasta -sizeout

usearch -derep_fulllength fastqFiles80.BE_forward.trim.contigs.good.trim.fasta -relabel DiCne. -fastaout fastqFiles80.BE_uniques.fasta -sizeout

usearch -derep_fulllength fastqFiles79.BE_forward.trim.contigs.good.trim.fasta -relabel DiCene. -fastaout fastqFiles79.BE_uniques.fasta -sizeout

usearch -derep_fulllength fastqFiles78.BE_forward.trim.contigs.good.trim.fasta -relabel Dic4. -fastaout fastqFiles78.BE_uniques.fasta -sizeout

usearch -derep_fulllength fastqFiles77.BE_forward.trim.contigs.good.trim.fasta -relabel Dic3. -fastaout fastqFiles77.BE_uniques.fasta -sizeout

usearch -derep_fulllength fastqFiles76.BE_forward.trim.contigs.good.trim.fasta -relabel Dic2. -fastaout fastqFiles76.BE_uniques.fasta -sizeout

usearch -derep_fulllength fastqFiles75.BE_forward.trim.contigs.good.trim.fasta -relabel Dic1. -fastaout fastqFiles75.BE_uniques.fasta -sizeout

usearch -derep_fulllength fastqFiles74.BE_forward.trim.contigs.good.trim.fasta -relabel DePne. -fastaout fastqFiles74.BE_uniques.fasta -sizeout

usearch -derep_fulllength fastqFiles73.BE_forward.trim.contigs.good.trim.fasta -relabel DeP4. -fastaout fastqFiles73.BE_uniques.fasta -sizeout

usearch -derep_fulllength fastqFiles72.BE_forward.trim.contigs.good.trim.fasta -relabel DeP3. -fastaout fastqFiles72.BE_uniques.fasta -sizeout

usearch -derep_fulllength fastqFiles71.BE_forward.trim.contigs.good.trim.fasta -relabel DeP2. -fastaout fastqFiles71.BE_uniques.fasta -sizeout

usearch -derep_fulllength fastqFiles70.BE_forward.trim.contigs.good.trim.fasta -relabel DeP1. -fastaout fastqFiles70.BE_uniques.fasta -sizeout

usearch -derep_fulllength fastqFiles69.BE_forward.trim.contigs.good.trim.fasta -relabel DeCene. -fastaout fastqFiles69.BE_uniques.fasta -sizeout

usearch -derep_fulllength fastqFiles68.BE_forward.trim.contigs.good.trim.fasta -relabel Dec4. -fastaout fastqFiles68.BE_uniques.fasta -sizeout

usearch -derep_fulllength fastqFiles67.BE_forward.trim.contigs.good.trim.fasta -relabel Dec3. -fastaout fastqFiles67.BE_uniques.fasta -sizeout

usearch -derep_fulllength fastqFiles66.BE_forward.trim.contigs.good.trim.fasta -relabel Dec2. -fastaout fastqFiles66.BE_uniques.fasta -sizeout

usearch -derep_fulllength fastqFiles65.BE_forward.trim.contigs.good.trim.fasta -relabel Dec1. -fastaout fastqFiles65.BE_uniques.fasta -sizeout

usearch -derep_fulllength fastqFiles64.BE_forward.trim.contigs.good.trim.fasta -relabel DdPne. -fastaout fastqFiles64.BE_uniques.fasta -sizeout

usearch -derep_fulllength fastqFiles63.BE_forward.trim.contigs.good.trim.fasta -relabel DdPene. -fastaout fastqFiles63.BE_uniques.fasta -sizeout

usearch -derep_fulllength fastqFiles62.BE_forward.trim.contigs.good.trim.fasta -relabel DdP4. -fastaout fastqFiles62.BE_uniques.fasta -sizeout

usearch -derep_fulllength fastqFiles61.BE_forward.trim.contigs.good.trim.fasta -relabel DdP3. -fastaout fastqFiles61.BE_uniques.fasta -sizeout

usearch -derep_fulllength fastqFiles60.BE_forward.trim.contigs.good.trim.fasta -relabel DdP2. -fastaout fastqFiles60.BE_uniques.fasta -sizeout

usearch -derep_fulllength fastqFiles59.BE_forward.trim.contigs.good.trim.fasta -relabel DdP1. -fastaout fastqFiles59.BE_uniques.fasta -sizeout

usearch -derep_fulllength fastqFiles58.BE_forward.trim.contigs.good.trim.fasta -relabel DdCne. -fastaout fastqFiles58.BE_uniques.fasta -sizeout

usearch -derep_fulllength fastqFiles57.BE_forward.trim.contigs.good.trim.fasta -relabel DdCene. -fastaout fastqFiles57.BE_uniques.fasta -sizeout

usearch -derep_fulllength fastqFiles56.BE_forward.trim.contigs.good.trim.fasta -relabel DdC4. -fastaout fastqFiles56.BE_uniques.fasta -sizeout

usearch -derep_fulllength fastqFiles55.BE_forward.trim.contigs.good.trim.fasta -relabel DdC3. -fastaout fastqFiles55.BE_uniques.fasta -sizeout

usearch -derep_fulllength fastqFiles54.BE_forward.trim.contigs.good.trim.fasta -relabel DdC2. -fastaout fastqFiles54.BE_uniques.fasta -sizeout

usearch -derep_fulllength fastqFiles53.BE_forward.trim.contigs.good.trim.fasta -relabel DdC1. -fastaout fastqFiles53.BE_uniques.fasta -sizeout

usearch -derep_fulllength fastqFiles52.BE_forward.trim.contigs.good.trim.fasta -relabel DbPne. -fastaout fastqFiles52.BE_uniques.fasta -sizeout

usearch -derep_fulllength fastqFiles51.BE_forward.trim.contigs.good.trim.fasta -relabel DbPene. -fastaout fastqFiles51.BE_uniques.fasta -sizeout

usearch -derep_fulllength fastqFiles50.BE_forward.trim.contigs.good.trim.fasta -relabel DbP4. -fastaout fastqFiles50.BE_uniques.fasta -sizeout

usearch -derep_fulllength fastqFiles49.BE_forward.trim.contigs.good.trim.fasta -relabel DbP3. -fastaout fastqFiles49.BE_uniques.fasta -sizeout

usearch -derep_fulllength fastqFiles48.BE_forward.trim.contigs.good.trim.fasta -relabel DbP2. -fastaout fastqFiles48.BE_uniques.fasta -sizeout

usearch -derep_fulllength fastqFiles47.BE_forward.trim.contigs.good.trim.fasta -relabel DbP1. -fastaout fastqFiles47.BE_uniques.fasta -sizeout

usearch -derep_fulllength fastqFiles46.BE_forward.trim.contigs.good.trim.fasta -relabel DbCne. -fastaout fastqFiles46.BE_uniques.fasta -sizeout

usearch -derep_fulllength fastqFiles45.BE_forward.trim.contigs.good.trim.fasta -relabel DbCene. -fastaout fastqFiles45.BE_uniques.fasta -sizeout

usearch -derep_fulllength fastqFiles44.BE_forward.trim.contigs.good.trim.fasta -relabel DbC4. -fastaout fastqFiles44.BE_uniques.fasta -sizeout

usearch -derep_fulllength fastqFiles43.BE_forward.trim.contigs.good.trim.fasta -relabel DbC3. -fastaout fastqFiles43.BE_uniques.fasta -sizeout

usearch -derep_fulllength fastqFiles42.BE_forward.trim.contigs.good.trim.fasta -relabel DbC2. -fastaout fastqFiles42.BE_uniques.fasta -sizeout

usearch -derep_fulllength fastqFiles41.BE_forward.trim.contigs.good.trim.fasta -relabel DbC1. -fastaout fastqFiles41.BE_uniques.fasta -sizeout

usearch -derep_fulllength fastqFiles40.BE_forward.trim.contigs.good.trim.fasta -relabel JiPne. -fastaout fastqFiles40.BE_uniques.fasta -sizeout

usearch -derep_fulllength fastqFiles39.BE_forward.trim.contigs.good.trim.fasta -relabel Jiene. -fastaout fastqFiles39.BE_uniques.fasta -sizeout

usearch -derep_fulllength fastqFiles38.BE_forward.trim.contigs.good.trim.fasta -relabel JePne. -fastaout fastqFiles38.BE_uniques.fasta -sizeout

usearch -derep_fulllength fastqFiles37.BE_forward.trim.contigs.good.trim.fasta -relabel Jeene. -fastaout fastqFiles37.BE_uniques.fasta -sizeout

usearch -derep_fulllength fastqFiles36.BE_forward.trim.contigs.good.trim.fasta -relabel JdPne. -fastaout fastqFiles36.BE_uniques.fasta -sizeout

usearch -derep_fulllength fastqFiles35.BE_forward.trim.contigs.good.trim.fasta -relabel JdPene. -fastaout fastqFiles35.BE_uniques.fasta -sizeout

usearch -derep_fulllength fastqFiles34.BE_forward.trim.contigs.good.trim.fasta -relabel JiP454. -fastaout fastqFiles34.BE_uniques.fasta -sizeout

usearch -derep_fulllength fastqFiles33.BE_forward.trim.contigs.good.trim.fasta -relabel JiP453. -fastaout fastqFiles33.BE_uniques.fasta -sizeout

usearch -derep_fulllength fastqFiles32.BE_forward.trim.contigs.good.trim.fasta -relabel JiP452. -fastaout fastqFiles32.BE_uniques.fasta -sizeout

usearch -derep_fulllength fastqFiles31.BE_forward.trim.contigs.good.trim.fasta -relabel JiP451. -fastaout fastqFiles31.BE_uniques.fasta -sizeout

usearch -derep_fulllength fastqFiles30.BE_forward.trim.contigs.good.trim.fasta -relabel JiN454. -fastaout fastqFiles30.BE_uniques.fasta -sizeout

usearch -derep_fulllength fastqFiles29.BE_forward.trim.contigs.good.trim.fasta -relabel JiN453. -fastaout fastqFiles29.BE_uniques.fasta -sizeout

usearch -derep_fulllength fastqFiles28.BE_forward.trim.contigs.good.trim.fasta -relabel JiN452. -fastaout fastqFiles28.BE_uniques.fasta -sizeout

usearch -derep_fulllength fastqFiles27.BE_forward.trim.contigs.good.trim.fasta -relabel JiN451. -fastaout fastqFiles27.BE_uniques.fasta -sizeout

usearch -derep_fulllength fastqFiles26.BE_forward.trim.contigs.good.trim.fasta -relabel Jep454. -fastaout fastqFiles26.BE_uniques.fasta -sizeout

usearch -derep_fulllength fastqFiles25.BE_forward.trim.contigs.good.trim.fasta -relabel Jep453. -fastaout fastqFiles25.BE_uniques.fasta -sizeout

usearch -derep_fulllength fastqFiles24.BE_forward.trim.contigs.good.trim.fasta -relabel Jep452. -fastaout fastqFiles24.BE_uniques.fasta -sizeout

usearch -derep_fulllength fastqFiles23.BE_forward.trim.contigs.good.trim.fasta -relabel Jep451. -fastaout fastqFiles23.BE_uniques.fasta -sizeout

usearch -derep_fulllength fastqFiles22.BE_forward.trim.contigs.good.trim.fasta -relabel JeN454. -fastaout fastqFiles22.BE_uniques.fasta -sizeout

usearch -derep_fulllength fastqFiles21.BE_forward.trim.contigs.good.trim.fasta -relabel JeN453. -fastaout fastqFiles21.BE_uniques.fasta -sizeout

usearch -derep_fulllength fastqFiles20.BE_forward.trim.contigs.good.trim.fasta -relabel JeN452. -fastaout fastqFiles20.BE_uniques.fasta -sizeout

usearch -derep_fulllength fastqFiles19.BE_forward.trim.contigs.good.trim.fasta -relabel JeN451. -fastaout fastqFiles19.BE_uniques.fasta -sizeout

usearch -derep_fulllength fastqFiles18.BE_forward.trim.contigs.good.trim.fasta -relabel Jdp454. -fastaout fastqFiles18.BE_uniques.fasta -sizeout

usearch -derep_fulllength fastqFiles17.BE_forward.trim.contigs.good.trim.fasta -relabel Jdp453. -fastaout fastqFiles17.BE_uniques.fasta -sizeout

usearch -derep_fulllength fastqFiles16.BE_forward.trim.contigs.good.trim.fasta -relabel Jdp452. -fastaout fastqFiles16.BE_uniques.fasta -sizeout

usearch -derep_fulllength fastqFiles15.BE_forward.trim.contigs.good.trim.fasta -relabel Jdp451. -fastaout fastqFiles15.BE_uniques.fasta -sizeout

usearch -derep_fulllength fastqFiles14.BE_forward.trim.contigs.good.trim.fasta -relabel JdN454. -fastaout fastqFiles14.BE_uniques.fasta -sizeout

usearch -derep_fulllength fastqFiles13.BE_forward.trim.contigs.good.trim.fasta -relabel JdN453. -fastaout fastqFiles13.BE_uniques.fasta -sizeout

usearch -derep_fulllength fastqFiles12.BE_forward.trim.contigs.good.trim.fasta -relabel JdN452. -fastaout fastqFiles12.BE_uniques.fasta -sizeout

usearch -derep_fulllength fastqFiles11.BE_forward.trim.contigs.good.trim.fasta -relabel JdN451. -fastaout fastqFiles11.BE_uniques.fasta -sizeout

usearch -derep_fulllength fastqFiles10.BE_forward.trim.contigs.good.trim.fasta -relabel JbPne. -fastaout fastqFiles10.BE_uniques.fasta -sizeout

usearch -derep_fulllength fastqFiles9.BE_forward.trim.contigs.good.trim.fasta -relabel JbP454. -fastaout fastqFiles9.BE_uniques.fasta -sizeout

usearch -derep_fulllength fastqFiles8.BE_forward.trim.contigs.good.trim.fasta -relabel JbP453. -fastaout fastqFiles8.BE_uniques.fasta -sizeout

usearch -derep_fulllength fastqFiles7.BE_forward.trim.contigs.good.trim.fasta -relabel JbP452. -fastaout fastqFiles7.BE_uniques.fasta -sizeout

usearch -derep_fulllength fastqFiles6.BE_forward.trim.contigs.good.trim.fasta -relabel JbP451. -fastaout fastqFiles6.BE_uniques.fasta -sizeout

usearch -derep_fulllength fastqFiles5.BE_forward.trim.contigs.good.trim.fasta -relabel JbN454. -fastaout fastqFiles5.BE_uniques.fasta -sizeout

usearch -derep_fulllength fastqFiles4.BE_forward.trim.contigs.good.trim.fasta -relabel JbN453. -fastaout fastqFiles4.BE_uniques.fasta -sizeout

usearch -derep_fulllength fastqFiles3.BE_forward.trim.contigs.good.trim.fasta -relabel JbN452. -fastaout fastqFiles3.BE_uniques.fasta -sizeout

usearch -derep_fulllength fastqFiles2.BE_forward.trim.contigs.good.trim.fasta -relabel JbN451. -fastaout fastqFiles2.BE_uniques.fasta -sizeout

usearch -derep_fulllength fastqFiles1.BE_forward.trim.contigs.good.trim.fasta -relabel Jbene. -fastaout fastqFiles1.BE_uniques.fasta -sizeout

usearch -cluster_otus fastqFiles84.BE_uniques.fasta -sizein -minsize 1 -otu_radius_pct 2.0 -sizeout -otus fastqFiles84.BE_otus.fa

usearch -cluster_otus fastqFiles85.BE_uniques.fasta -sizein -minsize 1 -otu_radius_pct 2.0 -sizeout -otus fastqFiles85.BE_otus.fa

usearch -cluster_otus fastqFiles83.BE_uniques.fasta -sizein -minsize 1 -otu_radius_pct 2.0 -sizeout -otus fastqFiles83.BE_otus.fa

usearch -cluster_otus fastqFiles82.BE_uniques.fasta -sizein -minsize 1 -otu_radius_pct 2.0 -sizeout -otus fastqFiles82.BE_otus.fa

usearch -cluster_otus fastqFiles81.BE_uniques.fasta -sizein -minsize 1 -otu_radius_pct 2.0 -sizeout -otus fastqFiles81.BE_otus.fa

usearch -cluster_otus fastqFiles80.BE_uniques.fasta -sizein -minsize 1 -otu_radius_pct 2.0 -sizeout -otus fastqFiles80.BE_otus.fa

usearch -cluster_otus fastqFiles79.BE_uniques.fasta -sizein -minsize 1 -otu_radius_pct 2.0 -sizeout -otus fastqFiles79.BE_otus.fa

usearch -cluster_otus fastqFiles78.BE_uniques.fasta -sizein -minsize 1 -otu_radius_pct 2.0 -sizeout -otus fastqFiles78.BE_otus.fa

usearch -cluster_otus fastqFiles77.BE_uniques.fasta -sizein -minsize 1 -otu_radius_pct 2.0 -sizeout -otus fastqFiles77.BE_otus.fa

usearch -cluster_otus fastqFiles76.BE_uniques.fasta -sizein -minsize 1 -otu_radius_pct 2.0 -sizeout -otus fastqFiles76.BE_otus.fa

usearch -cluster_otus fastqFiles75.BE_uniques.fasta -sizein -minsize 1 -otu_radius_pct 2.0 -sizeout -otus fastqFiles75.BE_otus.fa

usearch -cluster_otus fastqFiles74.BE_uniques.fasta -sizein -minsize 1 -otu_radius_pct 2.0 -sizeout -otus fastqFiles74.BE_otus.fa

usearch -cluster_otus fastqFiles73.BE_uniques.fasta -sizein -minsize 1 -otu_radius_pct 2.0 -sizeout -otus fastqFiles73.BE_otus.fa

usearch -cluster_otus fastqFiles72.BE_uniques.fasta -sizein -minsize 1 -otu_radius_pct 2.0 -sizeout -otus fastqFiles72.BE_otus.fa

usearch -cluster_otus fastqFiles71.BE_uniques.fasta -sizein -minsize 1 -otu_radius_pct 2.0 -sizeout -otus fastqFiles71.BE_otus.fa

usearch -cluster_otus fastqFiles70.BE_uniques.fasta -sizein -minsize 1 -otu_radius_pct 2.0 -sizeout -otus fastqFiles70.BE_otus.fa

usearch -cluster_otus fastqFiles69.BE_uniques.fasta -sizein -minsize 1 -otu_radius_pct 2.0 -sizeout -otus fastqFiles69.BE_otus.fa

usearch -cluster_otus fastqFiles68.BE_uniques.fasta -sizein -minsize 1 -otu_radius_pct 2.0 -sizeout -otus fastqFiles68.BE_otus.fa

usearch -cluster_otus fastqFiles67.BE_uniques.fasta -sizein -minsize 1 -otu_radius_pct 2.0 -sizeout -otus fastqFiles67.BE_otus.fa

usearch -cluster_otus fastqFiles66.BE_uniques.fasta -sizein -minsize 1 -otu_radius_pct 2.0 -sizeout -otus fastqFiles66.BE_otus.fa

usearch -cluster_otus fastqFiles65.BE_uniques.fasta -sizein -minsize 1 -otu_radius_pct 2.0 -sizeout -otus fastqFiles65.BE_otus.fa

usearch -cluster_otus fastqFiles64.BE_uniques.fasta -sizein -minsize 1 -otu_radius_pct 2.0 -sizeout -otus fastqFiles64.BE_otus.fa

usearch -cluster_otus fastqFiles63.BE_uniques.fasta -sizein -minsize 1 -otu_radius_pct 2.0 -sizeout -otus fastqFiles63.BE_otus.fa

usearch -cluster_otus fastqFiles62.BE_uniques.fasta -sizein -minsize 1 -otu_radius_pct 2.0 -sizeout -otus fastqFiles62.BE_otus.fa

usearch -cluster_otus fastqFiles61.BE_uniques.fasta -sizein -minsize 1 -otu_radius_pct 2.0 -sizeout -otus fastqFiles61.BE_otus.fa

usearch -cluster_otus fastqFiles60.BE_uniques.fasta -sizein -minsize 1 -otu_radius_pct 2.0 -sizeout -otus fastqFiles60.BE_otus.fa

usearch -cluster_otus fastqFiles59.BE_uniques.fasta -sizein -minsize 1 -otu_radius_pct 2.0 -sizeout -otus fastqFiles59.BE_otus.fa

usearch -cluster_otus fastqFiles58.BE_uniques.fasta -sizein -minsize 1 -otu_radius_pct 2.0 -sizeout -otus fastqFiles58.BE_otus.fa

usearch -cluster_otus fastqFiles57.BE_uniques.fasta -sizein -minsize 1 -otu_radius_pct 2.0 -sizeout -otus fastqFiles57.BE_otus.fa

usearch -cluster_otus fastqFiles56.BE_uniques.fasta -sizein -minsize 1 -otu_radius_pct 2.0 -sizeout -otus fastqFiles56.BE_otus.fa

usearch -cluster_otus fastqFiles55.BE_uniques.fasta -sizein -minsize 1 -otu_radius_pct 2.0 -sizeout -otus fastqFiles55.BE_otus.fa

usearch -cluster_otus fastqFiles54.BE_uniques.fasta -sizein -minsize 1 -otu_radius_pct 2.0 -sizeout -otus fastqFiles54.BE_otus.fa

usearch -cluster_otus fastqFiles53.BE_uniques.fasta -sizein -minsize 1 -otu_radius_pct 2.0 -sizeout -otus fastqFiles53.BE_otus.fa

usearch -cluster_otus fastqFiles52.BE_uniques.fasta -sizein -minsize 1 -otu_radius_pct 2.0 -sizeout -otus fastqFiles52.BE_otus.fa

usearch -cluster_otus fastqFiles51.BE_uniques.fasta -sizein -minsize 1 -otu_radius_pct 2.0 -sizeout -otus fastqFiles51.BE_otus.fa

usearch -cluster_otus fastqFiles50.BE_uniques.fasta -sizein -minsize 1 -otu_radius_pct 2.0 -sizeout -otus fastqFiles50.BE_otus.fa

usearch -cluster_otus fastqFiles49.BE_uniques.fasta -sizein -minsize 1 -otu_radius_pct 2.0 -sizeout -otus fastqFiles49.BE_otus.fa

usearch -cluster_otus fastqFiles48.BE_uniques.fasta -sizein -minsize 1 -otu_radius_pct 2.0 -sizeout -otus fastqFiles48.BE_otus.fa

usearch -cluster_otus fastqFiles47.BE_uniques.fasta -sizein -minsize 1 -otu_radius_pct 2.0 -sizeout -otus fastqFiles47.BE_otus.fa

usearch -cluster_otus fastqFiles46.BE_uniques.fasta -sizein -minsize 1 -otu_radius_pct 2.0 -sizeout -otus fastqFiles46.BE_otus.fa

usearch -cluster_otus fastqFiles45.BE_uniques.fasta -sizein -minsize 1 -otu_radius_pct 2.0 -sizeout -otus fastqFiles45.BE_otus.fa

usearch -cluster_otus fastqFiles44.BE_uniques.fasta -sizein -minsize 1 -otu_radius_pct 2.0 -sizeout -otus fastqFiles44.BE_otus.fa

usearch -cluster_otus fastqFiles43.BE_uniques.fasta -sizein -minsize 1 -otu_radius_pct 2.0 -sizeout -otus fastqFiles43.BE_otus.fa

usearch -cluster_otus fastqFiles42.BE_uniques.fasta -sizein -minsize 1 -otu_radius_pct 2.0 -sizeout -otus fastqFiles42.BE_otus.fa

usearch -cluster_otus fastqFiles41.BE_uniques.fasta -sizein -minsize 1 -otu_radius_pct 2.0 -sizeout -otus fastqFiles41.BE_otus.fa

usearch -cluster_otus fastqFiles40.BE_uniques.fasta -sizein -minsize 1 -otu_radius_pct 2.0 -sizeout -otus fastqFiles40.BE_otus.fa

usearch -cluster_otus fastqFiles39.BE_uniques.fasta -sizein -minsize 1 -otu_radius_pct 2.0 -sizeout -otus fastqFiles39.BE_otus.fa

usearch -cluster_otus fastqFiles38.BE_uniques.fasta -sizein -minsize 1 -otu_radius_pct 2.0 -sizeout -otus fastqFiles38.BE_otus.fa

usearch -cluster_otus fastqFiles37.BE_uniques.fasta -sizein -minsize 1 -otu_radius_pct 2.0 -sizeout -otus fastqFiles37.BE_otus.fa

usearch -cluster_otus fastqFiles36.BE_uniques.fasta -sizein -minsize 1 -otu_radius_pct 2.0 -sizeout -otus fastqFiles36.BE_otus.fa

usearch -cluster_otus fastqFiles35.BE_uniques.fasta -sizein -minsize 1 -otu_radius_pct 2.0 -sizeout -otus fastqFiles35.BE_otus.fa

usearch -cluster_otus fastqFiles34.BE_uniques.fasta -sizein -minsize 1 -otu_radius_pct 2.0 -sizeout -otus fastqFiles34.BE_otus.fa

usearch -cluster_otus fastqFiles33.BE_uniques.fasta -sizein -minsize 1 -otu_radius_pct 2.0 -sizeout -otus fastqFiles33.BE_otus.fa

usearch -cluster_otus fastqFiles32.BE_uniques.fasta -sizein -minsize 1 -otu_radius_pct 2.0 -sizeout -otus fastqFiles32.BE_otus.fa

usearch -cluster_otus fastqFiles31.BE_uniques.fasta -sizein -minsize 1 -otu_radius_pct 2.0 -sizeout -otus fastqFiles31.BE_otus.fa

usearch -cluster_otus fastqFiles30.BE_uniques.fasta -sizein -minsize 1 -otu_radius_pct 2.0 -sizeout -otus fastqFiles30.BE_otus.fa

usearch -cluster_otus fastqFiles29.BE_uniques.fasta -sizein -minsize 1 -otu_radius_pct 2.0 -sizeout -otus fastqFiles29.BE_otus.fa

usearch -cluster_otus fastqFiles28.BE_uniques.fasta -sizein -minsize 1 -otu_radius_pct 2.0 -sizeout -otus fastqFiles28.BE_otus.fa

usearch -cluster_otus fastqFiles27.BE_uniques.fasta -sizein -minsize 1 -otu_radius_pct 2.0 -sizeout -otus fastqFiles27.BE_otus.fa

usearch -cluster_otus fastqFiles26.BE_uniques.fasta -sizein -minsize 1 -otu_radius_pct 2.0 -sizeout -otus fastqFiles26.BE_otus.fa

usearch -cluster_otus fastqFiles25.BE_uniques.fasta -sizein -minsize 1 -otu_radius_pct 2.0 -sizeout -otus fastqFiles25.BE_otus.fa

usearch -cluster_otus fastqFiles24.BE_uniques.fasta -sizein -minsize 1 -otu_radius_pct 2.0 -sizeout -otus fastqFiles24.BE_otus.fa

usearch -cluster_otus fastqFiles23.BE_uniques.fasta -sizein -minsize 1 -otu_radius_pct 2.0 -sizeout -otus fastqFiles23.BE_otus.fa

usearch -cluster_otus fastqFiles22.BE_uniques.fasta -sizein -minsize 1 -otu_radius_pct 2.0 -sizeout -otus fastqFiles22.BE_otus.fa

usearch -cluster_otus fastqFiles21.BE_uniques.fasta -sizein -minsize 1 -otu_radius_pct 2.0 -sizeout -otus fastqFiles21.BE_otus.fa

usearch -cluster_otus fastqFiles20.BE_uniques.fasta -sizein -minsize 1 -otu_radius_pct 2.0 -sizeout -otus fastqFiles20.BE_otus.fa

usearch -cluster_otus fastqFiles19.BE_uniques.fasta -sizein -minsize 1 -otu_radius_pct 2.0 -sizeout -otus fastqFiles19.BE_otus.fa

usearch -cluster_otus fastqFiles18.BE_uniques.fasta -sizein -minsize 1 -otu_radius_pct 2.0 -sizeout -otus fastqFiles18.BE_otus.fa

usearch -cluster_otus fastqFiles17.BE_uniques.fasta -sizein -minsize 1 -otu_radius_pct 2.0 -sizeout -otus fastqFiles17.BE_otus.fa

usearch -cluster_otus fastqFiles16.BE_uniques.fasta -sizein -minsize 1 -otu_radius_pct 2.0 -sizeout -otus fastqFiles16.BE_otus.fa

usearch -cluster_otus fastqFiles15.BE_uniques.fasta -sizein -minsize 1 -otu_radius_pct 2.0 -sizeout -otus fastqFiles15.BE_otus.fa

usearch -cluster_otus fastqFiles14.BE_uniques.fasta -sizein -minsize 1 -otu_radius_pct 2.0 -sizeout -otus fastqFiles14.BE_otus.fa

usearch -cluster_otus fastqFiles13.BE_uniques.fasta -sizein -minsize 1 -otu_radius_pct 2.0 -sizeout -otus fastqFiles13.BE_otus.fa

usearch -cluster_otus fastqFiles12.BE_uniques.fasta -sizein -minsize 1 -otu_radius_pct 2.0 -sizeout -otus fastqFiles12.BE_otus.fa

usearch -cluster_otus fastqFiles11.BE_uniques.fasta -sizein -minsize 1 -otu_radius_pct 2.0 -sizeout -otus fastqFiles11.BE_otus.fa

usearch -cluster_otus fastqFiles10.BE_uniques.fasta -sizein -minsize 1 -otu_radius_pct 2.0 -sizeout -otus fastqFiles10.BE_otus.fa

usearch -cluster_otus fastqFiles9.BE_uniques.fasta -sizein -minsize 1 -otu_radius_pct 2.0 -sizeout -otus fastqFiles9.BE_otus.fa

usearch -cluster_otus fastqFiles8.BE_uniques.fasta -sizein -minsize 1 -otu_radius_pct 2.0 -sizeout -otus fastqFiles8.BE_otus.fa

usearch -cluster_otus fastqFiles7.BE_uniques.fasta -sizein -minsize 1 -otu_radius_pct 2.0 -sizeout -otus fastqFiles7.BE_otus.fa

usearch -cluster_otus fastqFiles6.BE_uniques.fasta -sizein -minsize 1 -otu_radius_pct 2.0 -sizeout -otus fastqFiles6.BE_otus.fa

usearch -cluster_otus fastqFiles5.BE_uniques.fasta -sizein -minsize 1 -otu_radius_pct 2.0 -sizeout -otus fastqFiles5.BE_otus.fa

usearch -cluster_otus fastqFiles4.BE_uniques.fasta -sizein -minsize 1 -otu_radius_pct 2.0 -sizeout -otus fastqFiles4.BE_otus.fa

usearch -cluster_otus fastqFiles3.BE_uniques.fasta -sizein -minsize 1 -otu_radius_pct 2.0 -sizeout -otus fastqFiles3.BE_otus.fa

usearch -cluster_otus fastqFiles2.BE_uniques.fasta -sizein -minsize 1 -otu_radius_pct 2.0 -sizeout -otus fastqFiles2.BE_otus.fa

usearch -cluster_otus fastqFiles1.BE_uniques.fasta -sizein -minsize 1 -otu_radius_pct 2.0 -sizeout -otus fastqFiles1.BE_otus.fa

mothur> merge.files(input=fastqFiles1.BE_otus.fa-fastqFiles2.BE_otus.fa-fastqFiles3.BE_otus.fa-fastqFiles4.BE_otus.fa-fastqFiles5.BE_otus.fa-fastqFiles6.BE_otus.fa-fastqFiles7.BE_otus.fa-fastqFiles8.BE_otus.fa-fastqFiles9.BE_otus.fa-fastqFiles10.BE_otus.fa-fastqFiles11.BE_otus.fa-fastqFiles12.BE_otus.fa-fastqFiles13.BE_otus.fa-fastqFiles14.BE_otus.fa-fastqFiles15.BE_otus.fa-fastqFiles16.BE_otus.fa-fastqFiles17.BE_otus.fa-fastqFiles18.BE_otus.fa-fastqFiles19.BE_otus.fa-fastqFiles20.BE_otus.fa-fastqFiles21.BE_otus.fa-fastqFiles22.BE_otus.fa-fastqFiles23.BE_otus.fa-fastqFiles24.BE_otus.fa-fastqFiles25.BE_otus.fa-fastqFiles26.BE_otus.fa-fastqFiles27.BE_otus.fa-fastqFiles28.BE_otus.fa-fastqFiles29.BE_otus.fa-fastqFiles30.BE_otus.fa-fastqFiles31.BE_otus.fa-fastqFiles32.BE_otus.fa-fastqFiles33.BE_otus.fa-fastqFiles34.BE_otus.fa-fastqFiles35.BE_otus.fa-fastqFiles36.BE_otus.fa-fastqFiles37.BE_otus.fa-fastqFiles38.BE_otus.fa-fastqFiles39.BE_otus.fa-fastqFiles40.BE_otus.fa-fastqFiles41.BE_otus.fa-fastqFiles42.BE_otus.fa-fastqFiles43.BE_otus.fa-fastqFiles44.BE_otus.fa-fastqFiles45.BE_otus.fa-fastqFiles46.BE_otus.fa-fastqFiles47.BE_otus.fa-fastqFiles48.BE_otus.fa-fastqFiles49.BE_otus.fa-fastqFiles50.BE_otus.fa-fastqFiles51.BE_otus.fa-fastqFiles52.BE_otus.fa-fastqFiles53.BE_otus.fa-fastqFiles54.BE_otus.fa-fastqFiles55.BE_otus.fa-fastqFiles56.BE_otus.fa-fastqFiles57.BE_otus.fa-fastqFiles58.BE_otus.fa-fastqFiles59.BE_otus.fa-fastqFiles60.BE_otus.fa-fastqFiles61.BE_otus.fa-fastqFiles62.BE_otus.fa-fastqFiles64.BE_otus.fa-fastqFiles65.BE_otus.fa-fastqFiles66.BE_otus.fa-fastqFiles67.BE_otus.fa-fastqFiles68.BE_otus.fa-fastqFiles70.BE_otus.fa-fastqFiles71.BE_otus.fa-fastqFiles72.BE_otus.fa-fastqFiles73.BE_otus.fa-fastqFiles74.BE_otus.fa-fastqFiles75.BE_otus.fa-fastqFiles76.BE_otus.fa-fastqFiles77.BE_otus.fa-fastqFiles78.BE_otus.fa-fastqFiles79.BE_otus.fa-fastqFiles80.BE_otus.fa-fastqFiles81.BE_otus.fa-fastqFiles82.BE_otus.fa-fastqFiles83.BE_otus.fa-fastqFiles84.BE_otus.fa-fastqFiles85.BE_otus.fa, output=otusALL_BE.fa)

usearch -sortbysize otusALL_BE.fa -fastaout otusALL_BE_sorted.fa

usearch -cluster_otus otusALL_BE_sorted.fa -minsize 1 -otu_radius_pct 3.0 -sizein -otus otusALL_BE_sorted_clustered_3.0.fa -sizeout -log cluster_otusALL_BEclustered3.0.log

usearch -usearch_global otusALL_BE.fa -db otusALL_BE_sorted_clustered_3.0.fa -strand plus -id 0.97 -mothur_shared_out otutab_3.0_BE.shared

[usearch -usearch_global fastqFiles84.BE_forward.trim.contigs.good.trim.fasta -db otusALL_BE_sorted_clustered_3.0.fa -strand plus -id 0.97 -mothur_shared_out otutab_3.0_BE_DiP4.shared

usearch -usearch_global fastqFiles80.BE_forward.trim.contigs.good.trim.fasta -db otusALL_BE_sorted_clustered_3.0.fa -strand plus -id 0.97 -mothur_shared_out otutab_3.0_BE_DiCne.shared]

Removed 44 OTUs based on their presence only or mainly in negative samples

saved as:

otusALL_BE_sorted_clustered_3.0.negaway.fa

blastn -db nt -query E:\EBAI\eDNA\eDNARun\otusALL_BE_sorted_clustered_3.0.negaway.fa -evalue 1e-20 -num_descriptions 100 -num_alignments 100 -out E:\EBAI\eDNA\eDNARun\otusALL_BE_sorted_clustered_3.0.taxonomy.txt -remote

MEGAN 6.5.10:

import otusALL_BE_sorted_clustered_3.0.taxonomy.txt

minimum bit score 100.0 (low because we want all metazoa to be separate)

top percentage 8.0

Minimum support percentage 0.0001

minimum support 1

Total: 15633

Not assigned: 39(0.25%)

No hits 7286(46.6%)

Export readName_to_taxonPath to files otusALL_BE_sorted_clustered.taxonomy-eukaryota.txt, otusALL_BE_sorted_clustered.taxonomy-bacteria.txt, otusALL_BE_sorted_clustered.taxonomy-notassigned.txt and otusALL_BE_sorted_clustered.taxonomy-nohits.txt

Results put together with OTU-table, manually.

2062 Metazoa

Since we are interested in Metazoa and monitoring them, we used this data to take out only Metazoa sequences.

edited fa file so that names have no size info, easier to do get.seqs command, saved as otusALL_BE_.fa.

mothur > get.seqs(accnos=BE_Metazoa.accnos, fasta=otusALL_BE_.fa)

output = otusALL_BE_.pick.fa, only metazoa sequences

Translated to aminoacids (frame 2,invertebrate mitochondrial) using http://www.bioinformatics.org/sms2/translate.html

otusALL_BE_.pick.translated.fa

Translated aligned using MAFFT online tool and checked for inconsistences (non-COI reads)

otusALL_BE_.pick.translated.aligned.fa

210 stop codons

we are looking for invertebrates, so I removed all seqs (68) that had stop codons in invertebrate code.

otusALL_BE_.pick.translated.aligned.stopsremoved.fa

otusALL_BE_.pick.translated.aligned.stopsremoved.removed.fa (68 seqs)

good qualitys in otusALL_BE_.pick.translated.aligned.stopsremoved.fa checked for non-COIs, those still removed.

no obvious non-CO1s left.

mothur > get.seqs(accnos=no_stops_BE.accnos, fasta=otusALL_BE_.pick.fa)

output = otusALL_BE_.pick.pick.fa, stops removed file, 1994 seqs left.

Metazoa blasted against BOLD v.4 Species Level Barcode Records ((2,669,681 Sequences/174,315 Species/64,199 Interim Species). Put together.

mothur > get.seqs(accnos=nomatch.accnos, fasta=otusALL_BE_.pick.pick.fa)

No match reads blasted against BOLD v.4 all barcode records.

Normalizations

mothur > sub.sample(shared=BE_otutab.lake.txt, size=12720)

mothur > sub.sample(shared=BE_otutab.river.txt, size=26837)

Combined F230 and BE:

We are interested in the identified Metazoa, and only invertebrates; vertebrates excluded. Put together the 2 fragments that have been identified.

Each species name will have only 1 OTU, thus merging OTUs if having the same name.

117 and 1521 OTUs in total in the two fragments in the lake and river site, respectively. After merging the OTUs (same name within 97%) and removing unidentified OTUs (OTUs not identifed using Species Level Barcode Records and which do not have species-named identity in All Barcode Records), 90 and 859 OTUs.

For Normalizations, put all fragments together and calculated minimum number of sequences, 32151 for lake, 60358 for river.

transpose.exe --limit 25000x25000 --win --transpose eDNA_species_level_river_otutab.txt > transposed.txt

mothur > sub.sample(shared=eDNA_species_level_lake_otutab.txt, size=32151)

mothur > sub.sample(shared=eDNA_species_level_river_otutab.txt, size=60358)

transpose.exe --limit 25000x25000 --win --transpose eDNA_species_level_river_otutab.usearch.subsample.txt > transposed.txt

Assemblage variation in R Studio:

> library(vegan)

Loading required package: permute

Loading required package: lattice

This is vegan 2.4-3

Warning messages:

1: package ‘vegan’ was built under R version 3.2.5

2: package ‘permute’ was built under R version 3.2.5

> setwd("F:/EBAI/eDNA/manuscript")

> getwd()

[1] "F:/EBAI/eDNA/manuscript"

> SpeciesRiver <- read.csv("SpeciesRiver.csv")

*SpeciesRiver.csv made from subsampled river species table without three first rows and then transposed. ";" replaced with "," in csv file.

dis <- vegdist(SpeciesRiver)

## Bray-Curtis distances between samples

groups <- factor(c(rep(1,4), rep(2,4), rep(3,4), rep(4,4), rep(5,4), rep(6,4), rep(7,4), rep(8,4)), labels = c("BufferCN","BufferPES","DryCN","DryPES","EtOHCN","EtOHPES","IceCN","IcePES"))

## Calculate multivariate dispersions

mod <- betadisper(dis, groups)

mod

## Draw a boxplot of the distances to centroid for each group

boxplot(mod)

head(mod)

##distances

## [1] 0.06089861 0.07044261 0.21915792 0.04897356 0.04974923 0.53826277 0.05722434

## [8] 0.05556376 0.03679669 0.07754429 0.09428406 0.10448200 0.15686730 0.14767888

##[15] 0.11553739 0.11641700 0.15114350 0.67407258 0.11357967 0.14496372 0.42078360

##[22] 0.06029910 0.08890373 0.07050273 0.08894779 0.07731446 0.06270050 0.08525648

##[29] 0.04887186 0.05494052 0.20010160 0.11780565

> SpeciesLake <- read.csv("SpeciesLake.csv")

dis <- vegdist(SpeciesLake)

groups <- factor(c(rep(1,4), rep(2,4), rep(3,4), rep(4,4), rep(5,4), rep(6,4), rep(7,4), rep(8,4)), labels = c("Buffer_direct","Buffer_pre","Dry_direct","Dry_pre","EtOH_direct","EtOH_pre","Ice_direct","Ice_pre"))

mod <- betadisper(dis, groups)

mod

boxplot(mod)

head(mod)

##distances

##[1] 0.5461572 0.5931123 0.6295367 0.4579294 0.5743213 0.6317579 0.6317213

##[8] 0.5742897 0.7691172 0.2375625 0.3453861 0.3227652 0.5874484 0.4528529

##[15] 0.6776767 0.4801557 0.1717263 0.3885036 0.4626133 0.4309580 0.6114847

##[22] 0.6014685 0.6078409 0.6178010 0.2354098 0.3568095 0.7207980 0.3535717

##[29] 0.5347718 0.5331208 0.6523743 0.6512762

> IdMetazoaLake <- read.csv("IdMetazoaLake.csv")

dis <- vegdist(IdMetazoaLake)

groups <- factor(c(rep(1,4), rep(2,4), rep(3,4), rep(4,4), rep(5,4), rep(6,4), rep(7,4), rep(8,4)), labels = c("Buffer_direct","Buffer_pre","Dry_direct","Dry_pre","EtOH_direct","EtOH_pre","Ice_direct","Ice_pre"))

mod <- betadisper(dis, groups)

mod

boxplot(mod)

head(mod)

##distances

##[1] 0.5887259 0.5148841 0.7088168 0.4173277 0.6122492 0.6123857 0.6123544

##[8] 0.6123776 0.7836096 0.1765887 0.3091986 0.1039287 0.4975282 0.5533895

##[15] 0.5403376 0.6266960 0.3232756 0.3607499 0.4020308 0.3791353 0.6024069

##[22] 0.6101859 0.6134918 0.6134335 0.3619525 0.3946857 0.6899221 0.4074120

##[29] 0.5871077 0.5813453 0.6204380 0.6271326

> MetazoaLake <- read.csv("MetazoaLake.csv")

dis <- vegdist(MetazoaLake)

groups <- factor(c(rep(1,4), rep(2,4), rep(3,4), rep(4,4), rep(5,4), rep(6,4), rep(7,4), rep(8,4)), labels = c("Buffer_direct","Buffer_pre","Dry_direct","Dry_pre","EtOH_direct","EtOH_pre","Ice_direct","Ice_pre"))

mod <- betadisper(dis, groups)

mod

boxplot(mod)

head(mod)

##distances

##[1] 0.54007248 0.19779617 0.46360730 0.14707731 0.04109451 0.19554946 0.18716577

##[8] 0.32670971 0.34271459 0.16318017 0.10435563 0.18730363 0.21274545 0.06672586

##[15] 0.14135564 0.16209235 0.10469341 0.15516626 0.16201539 0.11122171 0.15696711

##[22] 0.15952492 0.05943138 0.12282861 0.14134231 0.13862929 0.30433106 0.08849957

##[29] 0.52545550 0.14424133 0.22506913 0.28911781

> IdMetazoaRiver <- read.csv("IdMetazoaRiver.csv")

dis <- vegdist(IdMetazoaRiver)

groups <- factor(c(rep(1,4), rep(2,4), rep(3,4), rep(4,4), rep(5,4), rep(6,4), rep(7,4), rep(8,4)), labels = c("BufferCN","BufferPES","DryCN","DryPES","EtOHCN","EtOHPES","IceCN","IcePES"))

mod <- betadisper(dis, groups)

mod

boxplot(mod)

head(mod)

##distances

## [1] 0.06415061 0.04710629 0.16370321 0.04825529 0.05846629 0.56900822 0.04787083

## [8] 0.05737030 0.03968867 0.04722752 0.07428845 0.09098853 0.10627708 0.09932718

##[15] 0.10278557 0.08823342 0.15706714 0.68087431 0.09639765 0.22197770 0.17034357

##[22] 0.05993904 0.07526739 0.08572896 0.07804230 0.07132117 0.06339295 0.08602311

##[29] 0.09069691 0.07106333 0.30598990 0.05588385

> MetazoaRiver <- read.csv("MetazoaRiver.csv")

dis <- vegdist(MetazoaRiver)

groups <- factor(c(rep(1,4), rep(2,4), rep(3,4), rep(4,4), rep(5,4), rep(6,4), rep(7,4), rep(8,4)), labels = c("BufferCN","BufferPES","DryCN","DryPES","EtOHCN","EtOHPES","IceCN","IcePES"))

mod <- betadisper(dis, groups)

mod

boxplot(mod)

head(mod)

##distances

## [1] 0.06656819 0.10989095 0.17097644 0.04712245 0.06531678 0.56729560 0.07558454

## [8] 0.06169312 0.05233007 0.05114511 0.07902398 0.09609066 0.11073173 0.10388501

##[15] 0.10767580 0.09181350 0.16130180 0.68046727 0.10099864 0.22557902 0.17436499

##[22] 0.07578650 0.08175040 0.09265699 0.09310743 0.08219487 0.06811375 0.08987885

##[29] 0.09673332 0.08168837 0.31704343 0.07309830

> SpeciesRiver <- read.csv("SpeciesRiver.csv")

dis <- vegdist(SpeciesRiver, binary=TRUE)

## Sørensen distances between samples

groups <- factor(c(rep(1,4), rep(2,4), rep(3,4), rep(4,4), rep(5,4), rep(6,4), rep(7,4), rep(8,4)), labels = c("BufferCN","BufferPES","DryCN","DryPES","EtOHCN","EtOHPES","IceCN","IcePES"))

mod <- betadisper(dis, groups)

mod

boxplot(mod)

head(mod)

##distances

## [1] 0.1628323 0.1721858 0.2044287 0.1835964 0.2241673 0.2202653 0.1708422

## [8] 0.1720503 0.2127453 0.1718890 0.1732516 0.1644124 0.2135152 0.2181094

##[15] 0.2000872 0.1578797 0.1975767 0.2708719 0.1979328 0.2238574 0.2164833

##[22] 0.2132205 0.1791102 0.2405553 0.1901176 0.1747554 0.1705460 0.2164404

##[29] 0.1922618 0.2024423 0.2116663 0.2251585

> SpeciesLake <- read.csv("SpeciesLake.csv")

dis <- vegdist(SpeciesLake, binary=TRUE)

groups <- factor(c(rep(1,4), rep(2,4), rep(3,4), rep(4,4), rep(5,4), rep(6,4), rep(7,4), rep(8,4)), labels = c("Buffer_direct","Buffer_pre","Dry_direct","Dry_pre","EtOH_direct","EtOH_pre","Ice_direct","Ice_pre"))

mod <- betadisper(dis, groups)

mod

boxplot(mod)

head(mod)

##distances

## [1] 0.2865360 0.4664915 0.2778408 0.3299344 0.4681569 0.6848230 0.6837330

## [8] 0.4673213 0.5586988 0.2708301 0.1810785 0.2305725 0.4094385 0.5028117

##[15] 0.6175881 0.5249983 0.3795873 0.2302190 0.5226713 0.3808460 0.6083787

##[22] 0.3611089 0.5148319 0.7194641 0.3666949 0.3944517 0.3286999 0.2985737

##[29] 0.5157260 0.4308920 0.6317429 0.5840225

> IdMetazoaLake <- read.csv("IdMetazoaLake.csv")

dis <- vegdist(IdMetazoaLake, binary=TRUE)

groups <- factor(c(rep(1,4), rep(2,4), rep(3,4), rep(4,4), rep(5,4), rep(6,4), rep(7,4), rep(8,4)), labels = c("Buffer_direct","Buffer_pre","Dry_direct","Dry_pre","EtOH_direct","EtOH_pre","Ice_direct","Ice_pre"))

mod <- betadisper(dis, groups)

mod

boxplot(mod)

head(mod)

##distances

## [1] 0.38704932 0.34771331 0.34921919 0.22826246 0.60498824 0.60976636 0.60952931

## [8] 0.61834694 0.05887586 0.50004602 0.43254255 0.13251669 0.37308885 0.32697611

##[15] 0.33301846 0.67673171 0.40175572 0.45941618 0.51190426 0.39815133 0.34799448

##[22] 0.62129895 0.62371077 0.58275040 0.48788466 0.38097471 0.11755458 0.46370563

##[29] 0.46698014 0.48374473 0.41963375 0.69656254

> MetazoaLake <- read.csv("MetazoaLake.csv")

dis <- vegdist(MetazoaLake, binary=TRUE)

groups <- factor(c(rep(1,4), rep(2,4), rep(3,4), rep(4,4), rep(5,4), rep(6,4), rep(7,4), rep(8,4)), labels = c("Buffer_direct","Buffer_pre","Dry_direct","Dry_pre","EtOH_direct","EtOH_pre","Ice_direct","Ice_pre"))

mod <- betadisper(dis, groups)

mod

boxplot(mod)

head(mod)

##distances

## [1] 0.31168756 0.29659849 0.30959235 0.26930827 0.24493267 0.03347414 0.15676622

## [8] 0.24084724 0.22983668 0.30849435 0.27778965 0.28544674 0.21918371 0.23229392

##[15] 0.22100817 0.22518209 0.27415661 0.21115049 0.24436929 0.20526194 0.25155897

##[22] 0.29604670 0.23138487 0.14629619 0.31774994 0.22685971 0.19830141 0.24854176

##[29] 0.10939386 0.24025844 0.18399833 0.33628286

> IdMetazoaRiver <- read.csv("IdMetazoaRiver.csv")

dis <- vegdist(IdMetazoaRiver, binary=TRUE)

groups <- factor(c(rep(1,4), rep(2,4), rep(3,4), rep(4,4), rep(5,4), rep(6,4), rep(7,4), rep(8,4)), labels = c("BufferCN","BufferPES","DryCN","DryPES","EtOHCN","EtOHPES","IceCN","IcePES"))

mod <- betadisper(dis, groups)

mod

boxplot(mod)

head(mod)

##distances

## [1] 0.2178154 0.2224972 0.2054447 0.2027784 0.2389850 0.2626866 0.1920132

## [8] 0.2051664 0.2287663 0.2192110 0.2236966 0.1988096 0.2410657 0.2245746

##[15] 0.2156339 0.2318526 0.2439319 0.3799907 0.2256765 0.2142845 0.2377011

##[22] 0.2414843 0.2248443 0.2710079 0.2112807 0.2136276 0.2287449 0.2343079

##[29] 0.2668858 0.2509126 0.2133033 0.2644933

> MetazoaRiver <- read.csv("MetazoaRiver.csv")

dis <- vegdist(MetazoaRiver, binary=TRUE)

groups <- factor(c(rep(1,4), rep(2,4), rep(3,4), rep(4,4), rep(5,4), rep(6,4), rep(7,4), rep(8,4)), labels = c("BufferCN","BufferPES","DryCN","DryPES","EtOHCN","EtOHPES","IceCN","IcePES"))

mod <- betadisper(dis, groups)

mod

boxplot(mod)

head(mod)

##distances

## [1] 0.2670199 0.2657216 0.2627810 0.2573650 0.2795504 0.3119226 0.2596700

## [8] 0.2333352 0.2659569 0.2656701 0.2732189 0.2513155 0.2828725 0.2761769

##[15] 0.2609687 0.2867276 0.2801194 0.4171114 0.2661259 0.2685310 0.2826837

##[22] 0.2957231 0.2614963 0.3095497 0.2700873 0.2661024 0.2808631 0.2742257

##[29] 0.2892352 0.2994547 0.2693170 0.3200344

BE amplicon:

> library(vegan)

> setwd("F:/EBAI/eDNA/manuscript/analyysit")

> getwd()

> BE_IdMetazoaLake <- read.csv("BE_IdMetazoaLake.csv")

dis <- vegdist(BE_IdMetazoaLake)

groups <- factor(c(rep(1,4), rep(2,4), rep(3,4), rep(4,4), rep(5,4), rep(6,4), rep(7,4), rep(8,4)), labels = c("Buffer_direct","Buffer_pre","Dry_direct","Dry_pre","EtOH_direct","EtOH_pre","Ice_direct","Ice_pre"))

mod <- betadisper(dis, groups)

mod

boxplot(mod)

head(mod)

##distances

## 0.6015215 0.5635605 0.5711470 0.5496471 0.4668235

## [6] 0.6364076 0.7032302 0.4615729 0.6400450 0.3891752

##[11] 0.2594544 0.3414908 0.6903928 0.4615826 0.6729713

##[16] 0.4809891 0.4688695 0.6929073 0.4238055 0.4800938

##[21] 0.6062738 0.6149970 0.6071474 0.6149566 0.6968038

##[26] 0.3403219 0.6272349 0.2800280 0.5192031 0.5142072

##[31] 0.6570114 0.6628627

> BE_MetazoaLake <- read.csv("BE_MetazoaLake.csv")

dis <- vegdist(BE_MetazoaLake)

groups <- factor(c(rep(1,4), rep(2,4), rep(3,4), rep(4,4), rep(5,4), rep(6,4), rep(7,4), rep(8,4)), labels = c("Buffer_direct","Buffer_pre","Dry_direct","Dry_pre","EtOH_direct","EtOH_pre","Ice_direct","Ice_pre"))

mod <- betadisper(dis, groups)

mod

boxplot(mod)

head(mod)

##distances

## [1] 0.7496615 0.3092431 0.4692783 0.2655937 0.4316233

## [6] 0.4462215 0.3929343 0.3034053 0.3887695 0.3150564

##[11] 0.1380494 0.2956161 0.3498885 0.2134909 0.3187786

##[16] 0.2422057 0.2595533 0.4766131 0.2702142 0.2954692

##[21] 0.2917816 0.3022478 0.3490089 0.4359358 0.3634676

##[26] 0.2075760 0.5230272 0.1853219 0.5808828 0.4390415

##[31] 0.4795005 0.5399257

> BE_IdMetazoaRiver <- read.csv("BE_IdMetazoaRiver.csv")

dis <- vegdist(BE_IdMetazoaRiver)

groups <- factor(c(rep(1,4), rep(2,4), rep(3,4), rep(4,4), rep(5,4), rep(6,4), rep(7,4), rep(8,4)), labels = c("BufferCN","BufferPES","DryCN","DryPES","EtOHCN","EtOHPES","IceCN","IcePES"))

mod <- betadisper(dis, groups)

mod

boxplot(mod)

head(mod)

##distances

## [1] 0.08518082 0.12581011 0.24147622 0.09604594

## [5] 0.12079318 0.26704018 0.15308035 0.13595931

## [9] 0.07687287 0.10397158 0.11143737 0.08851815

##[13] 0.17510938 0.14067151 0.15887442 0.15727071

##[17] 0.32693019 0.72621780 0.24057576 0.39676657

##[21] 0.55140548 0.24070686 0.20691380 0.21330176

##[25] 0.13052510 0.11268419 0.12549591 0.10434744

##[29] 0.12649912 0.14806993 0.48349010 0.22952408

> BE_MetazoaRiver <- read.csv("BE_MetazoaRiver.csv")

dis <- vegdist(BE_MetazoaRiver)

groups <- factor(c(rep(1,4), rep(2,4), rep(3,4), rep(4,4), rep(5,4), rep(6,4), rep(7,4), rep(8,4)), labels = c("BufferCN","BufferPES","DryCN","DryPES","EtOHCN","EtOHPES","IceCN","IcePES"))

mod <- betadisper(dis, groups)

mod

boxplot(mod)

head(mod)

##distances

## [1] 0.1054663 0.1418218 0.2552459 0.1174076 0.1539347

## [6] 0.2754798 0.2082395 0.1643474 0.1129665 0.1217660

##[11] 0.1295852 0.1049165 0.2087958 0.1854796 0.2041789

##[16] 0.1835657 0.3234732 0.7128161 0.2541809 0.3914926

##[21] 0.5050220 0.2565841 0.2268128 0.2715639 0.1517746

##[26] 0.1338691 0.1407130 0.1293432 0.1671535 0.1764283

##[31] 0.4731490 0.2604721

>permutest(mod, pairwise = TRUE, permutations = 99)

Permutation test for homogeneity of multivariate dispersions

Permutation: free

Number of permutations: 99

Response: Distances

Df Sum Sq Mean Sq F N.Perm Pr(>F)

Groups 7 0.29456 0.042079 3.9202 99 0.01 **

Residuals 24 0.25761 0.010734

---

Signif. codes: 0 ‘***’ 0.001 ‘**’ 0.01 ‘*’ 0.05 ‘.’ 0.1 ‘ ’ 1

Pairwise comparisons:

(Observed p-value below diagonal, permuted p-value above diagonal)

BufferCN BufferPES DryCN DryPES EtOHCN EtOHPES IceCN IcePES

BufferCN 4.0000e-01 3.0000e-01 2.6000e-01 6.0000e-02 2.0000e-02 7.2000e-01 0.16

BufferPES 3.4096e-01 2.0000e-02 9.5000e-01 4.0000e-02 2.1000e-01 6.0000e-02 0.41

DryCN 3.1901e-01 2.5392e-02 1.0000e-02 3.0000e-02 3.0000e-02 1.0000e-02 0.05

DryPES 2.8926e-01 8.6598e-01 8.4461e-05 1.1000e-01 9.0000e-02 1.0000e-02 0.41

EtOHCN 4.7757e-02 8.1218e-02 2.4446e-02 6.8715e-02 4.5000e-01 4.0000e-02 0.32

EtOHPES 6.9759e-02 1.5169e-01 2.1743e-02 1.1267e-01 4.1283e-01 3.0000e-02 0.68

IceCN 6.5899e-01 7.0582e-02 2.4431e-02 4.1856e-04 3.2262e-02 3.3635e-02 0.10

IcePES 1.9775e-01 4.0192e-01 7.7081e-02 3.4123e-01 2.6796e-01 6.4988e-01 1.1716e-01

> BE_IdMetazoaLake <- read.csv("BE_IdMetazoaLake.csv")

dis <- vegdist(BE_IdMetazoaLake, binary=TRUE)

groups <- factor(c(rep(1,4), rep(2,4), rep(3,4), rep(4,4), rep(5,4), rep(6,4), rep(7,4), rep(8,4)), labels = c("Buffer_direct","Buffer_pre","Dry_direct","Dry_pre","EtOH_direct","EtOH_pre","Ice_direct","Ice_pre"))

mod <- betadisper(dis, groups)

mod

boxplot(mod)

head(mod)

##distances

## [1] 0.1684344 0.4319677 0.3832608 0.2445469 0.3748564

## [6] 0.3763035 0.8266430 0.2593456 0.3766531 0.3428179

##[11] 0.2291053 0.3881333 0.7213542 0.3640721 0.5127608

##[16] 0.5932615 0.3382456 0.5513122 0.1673117 0.4081196

##[21] 0.4736767 0.6776064 0.4749477 0.6786215 0.6102908

##[26] 0.2715338 0.3054115 0.1672204 0.3593687 0.3966187

##[31] 0.4535939 0.7904001

> BE_MetazoaLake <- read.csv("BE_MetazoaLake.csv")

dis <- vegdist(BE_MetazoaLake, binary=TRUE)

groups <- factor(c(rep(1,4), rep(2,4), rep(3,4), rep(4,4), rep(5,4), rep(6,4), rep(7,4), rep(8,4)), labels = c("Buffer_direct","Buffer_pre","Dry_direct","Dry_pre","EtOH_direct","EtOH_pre","Ice_direct","Ice_pre"))

mod <- betadisper(dis, groups)

mod

boxplot(mod)

head(mod)

##distances

## [1] 0.4358613 0.3605049 0.3823897 0.1888451 0.2842471

## [6] 0.4050862 0.2418450 0.0463358 0.2339452 0.2225738

##[11] 0.1767525 0.2902735 0.2898942 0.1989148 0.2572515

##[16] 0.1338081 0.2450615 0.2732473 0.2977330 0.1761555

##[21] 0.2400183 0.3421728 0.1572521 0.2471154 0.3399860

##[26] 0.2541185 0.2754651 0.2246289 0.2531163 0.3210738

##[31] 0.2353456 0.3227211

> BE_IdMetazoaRiver <- read.csv("BE_IdMetazoaRiver.csv")

dis <- vegdist(BE_IdMetazoaRiver, binary=TRUE)

groups <- factor(c(rep(1,4), rep(2,4), rep(3,4), rep(4,4), rep(5,4), rep(6,4), rep(7,4), rep(8,4)), labels = c("BufferCN","BufferPES","DryCN","DryPES","EtOHCN","EtOHPES","IceCN","IcePES"))

mod <- betadisper(dis, groups)

mod

boxplot(mod)

head(mod)

##distances

## [1] 0.2484618 0.2160148 0.2147831 0.2311625 0.2476709

## [6] 0.2808814 0.2108648 0.2368512 0.2083424 0.2032836

##[11] 0.1988131 0.2263324 0.1999288 0.2360303 0.2614607

##[16] 0.2060159 0.2562143 0.2497191 0.2372665 0.2949888

##[21] 0.2448399 0.2615128 0.2648257 0.2588930 0.2419223

##[26] 0.2194633 0.2163430 0.2370782 0.2518163 0.2377921

##[31] 0.2792710 0.2308456

> permutest(mod, pairwise = TRUE, permutations = 99)

Permutation test for homogeneity of multivariate dispersions

Permutation: free

Number of permutations: 99

Response: Distances

Df Sum Sq Mean Sq F N.Perm Pr(>F)

Groups 7 0.0087834 0.00125477 2.9832 99 0.02 *

Residuals 24 0.0100946 0.00042061

---

Signif. codes: 0 ‘***’ 0.001 ‘**’ 0.01 ‘*’ 0.05 ‘.’ 0.1 ‘ ’ 1

Pairwise comparisons:

(Observed p-value below diagonal, permuted p-value above diagonal)

BufferCN BufferPES DryCN DryPES EtOHCN EtOHPES IceCN IcePES

BufferCN 0.3600000 0.1100000 0.9100000 0.0700000 0.0500000 0.9400000 0.24

BufferPES 0.3571066 0.0500000 0.3400000 0.3500000 0.3300000 0.2800000 0.71

DryCN 0.1131568 0.0681481 0.3200000 0.0100000 0.0100000 0.0800000 0.01

DryPES 0.9180958 0.4049514 0.3229193 0.0800000 0.0600000 0.8800000 0.18

EtOHCN 0.0733405 0.4488697 0.0108447 0.1253400 0.8600000 0.0500000 0.60

EtOHPES 0.0161498 0.4088404 0.0006462 0.0779812 0.8828965 0.0200000 0.45

IceCN 0.9172916 0.3691150 0.0674652 0.8613962 0.0693965 0.0097014 0.19

IcePES 0.1442091 0.7559694 0.0161202 0.2255858 0.5795223 0.5365230 0.1389528

> BE_MetazoaRiver <- read.csv("BE_MetazoaRiver.csv")

dis <- vegdist(BE_MetazoaRiver, binary=TRUE)

groups <- factor(c(rep(1,4), rep(2,4), rep(3,4), rep(4,4), rep(5,4), rep(6,4), rep(7,4), rep(8,4)), labels = c("BufferCN","BufferPES","DryCN","DryPES","EtOHCN","EtOHPES","IceCN","IcePES"))

mod <- betadisper(dis, groups)

mod

boxplot(mod)

head(mod)

##distances

## [1] 0.2883799 0.2678572 0.2863281 0.2919742 0.2995652

## [6] 0.3417180 0.2712795 0.2709431 0.2608829 0.2617161

##[11] 0.2571680 0.2719123 0.2558930 0.2905942 0.3266923

##[16] 0.2789046 0.3221365 0.3071749 0.3042833 0.3407919

##[21] 0.3062126 0.3263856 0.3102753 0.3371116 0.2797405

##[26] 0.2659208 0.2837296 0.2827587 0.2880114 0.3079955

##[31] 0.3531244 0.2853845

> permutest(mod, pairwise = TRUE, permutations = 99)

Permutation test for homogeneity of multivariate dispersions

Permutation: free

Number of permutations: 99

Response: Distances

Df Sum Sq Mean Sq F N.Perm Pr(>F)

Groups 7 0.011442 0.00163461 3.5549 99 0.02 *

Residuals 24 0.011036 0.00045982

---

Signif. codes: 0 ‘***’ 0.001 ‘**’ 0.01 ‘*’ 0.05 ‘.’ 0.1 ‘ ’ 1

Pairwise comparisons:

(Observed p-value below diagonal, permuted p-value above diagonal)

BufferCN BufferPES DryCN DryPES EtOHCN EtOHPES IceCN IcePES

BufferCN 0.47000000 0.07000000 0.76000000 0.03000000 0.03000000 0.42000000 0.21

BufferPES 0.51125015 0.12000000 0.78000000 0.26000000 0.24000000 0.33000000 0.58

DryCN 0.01604838 0.10037141 0.21000000 0.01000000 0.01000000 0.05000000 0.03

DryPES 0.78960610 0.73651042 0.14756016 0.14000000 0.13000000 0.51000000 0.47

EtOHCN 0.01262984 0.26928778 0.00079459 0.12175161 0.87000000 0.02000000 0.62

EtOHPES 0.00671352 0.23253579 0.00034226 0.09943626 0.90312980 0.01000000 0.48

IceCN 0.44102856 0.33939549 0.02699427 0.53913218 0.00484067 0.00229326 0.09

IcePES 0.18213238 0.59759489 0.02880168 0.37546357 0.59500890 0.53398818 0.10792960

two and three-way ANOVAs in R:

> install.packages("agricolae")

install.packages("foreign")

> library(agricolae)

library(foreign)

> setwd("F:/EBAI/eDNA/manuscript/analyysit")

> getwd()

> RiverSpecies <- read.csv("RiverSpeciesANOVA.csv")

> str(RiverSpecies)

> fm <- aov(N_Species ~ Filter * Preservation, data = RiverSpecies)

> summary(fm)

Df Sum Sq Mean Sq F value Pr(>F)

Filter 1 14450 14450 25.065 4.09e-05 ***

Preservation 3 25616 8539 14.811 1.15e-05 ***

Filter:Preservation 3 4666 1555 2.698 0.0684 .

Residuals 24 13836 576

---

Signif. codes: 0 ‘***’ 0.001 ‘**’ 0.01 ‘*’ 0.05 ‘.’ 0.1 ‘ ’ 1

> HSD.test(fm, 'Filter', console=TRUE)

Groups, Treatments and means

a CN 241.8

b PES 199.2

> HSD.test(fm, 'Preservation', console=TRUE)

a Ice 242.2

a Dry 235.2

a Buffer 232.6

b EtOH 171.9

> fm <- aov(S_Species ~ Filter * Preservation, data = RiverSpecies)

> summary(fm)

Df Sum Sq Mean Sq F value Pr(>F)

Filter 1 0.00718 0.007176 1.694 0.205

Preservation 3 0.02210 0.007368 1.740 0.186

Filter:Preservation 3 0.01437 0.004789 1.131 0.356

Residuals 24 0.10164 0.004235

> fm <- aov(P_Species ~ Filter * Preservation, data = RiverSpecies)

> summary(fm)

Df Sum Sq Mean Sq F value Pr(>F)

Filter 1 0.00080 0.000799 0.189 0.668

Preservation 3 0.01854 0.006179 1.459 0.251

Filter:Preservation 3 0.02360 0.007866 1.857 0.164

Residuals 24 0.10164 0.004235

> fm <- aov(DB_Species ~ Filter * Preservation, data = RiverSpecies)

> summary(fm)

Df Sum Sq Mean Sq F value Pr(>F)

Filter 1 0.0011 0.001116 0.051 0.823

Preservation 3 0.0731 0.024371 1.119 0.361

Filter:Preservation 3 0.0425 0.014159 0.650 0.591

Residuals 24 0.5228 0.021783

> fm <- aov(DD_Species ~ Filter * Preservation, data = RiverSpecies)

> summary(fm)

Df Sum Sq Mean Sq F value Pr(>F)

Filter 1 0.000907 0.0009071 1.492 0.2338

Preservation 3 0.004352 0.0014506 2.385 0.0942 .

Filter:Preservation 3 0.001178 0.0003925 0.645 0.5934

Residuals 24 0.014596 0.0006082

---

Signif. codes: 0 ‘***’ 0.001 ‘**’ 0.01 ‘*’ 0.05 ‘.’ 0.1 ‘ ’ 1

> LakeSpecies <- read.csv("LakeSpeciesANOVA.csv")

> str(LakeSpecies)

> fm <- aov(N_Species ~ Filtration * Preservation, data = LakeSpecies)

> summary(fm)

Df Sum Sq Mean Sq F value Pr(>F)

Filtration 1 108.78 108.78 16.656 0.000429 ***

Preservation 3 44.59 14.86 2.276 0.105494

Filtration:Preservation 3 0.84 0.28 0.043 0.987809

Residuals 24 156.75 6.53

---

Signif. codes: 0 ‘***’ 0.001 ‘**’ 0.01 ‘*’ 0.05 ‘.’ 0.1 ‘ ’ 1

> HSD.test(fm, 'Filtration', console=TRUE)

a direct 7.875

b pre 4.188

> Lake <- read.csv("LakeANOVA.csv")

> str(Lake)

> fm <- aov(N_Metazoa ~ Amplicon * Filtration * Preservation, data = Lake)

> summary(fm)

Df Sum Sq Mean Sq F value Pr(>F)

Amplicon 1 5.6 5.6 0.551 0.46169

Filtration 1 1590.0 1590.0 155.202 < 2e-16 ***

Preservation 3 1145.7 381.9 37.277 1.37e-12 ***

Amplicon:Filtration 1 3.5 3.5 0.343 0.56075

Amplicon:Preservation 3 148.2 49.4 4.821 0.00516 **

Filtration:Preservation 3 59.5 19.8 1.937 0.13611

Amplicon:Filtration:Preservation 3 40.3 13.4 1.311 0.28165

Residuals 48 491.7 10.2

---

Signif. codes: 0 ‘***’ 0.001 ‘**’ 0.01 ‘*’ 0.05 ‘.’ 0.1 ‘ ’ 1

> HSD.test(fm, 'Filtration', console=TRUE)

Groups, Treatments and means

a direct 29.91

b pre 19.94

> HSD.test(fm, 'Preservation', console=TRUE)

Groups, Treatments and means

a Dry 31.62

b Ice 24.69

b EtOH 23.38

c Buffer 20

> tx <- with(Lake, interaction(Amplicon, Preservation))

> fm <- aov(N_Metazoa ~ tx, data = Lake)

> HSD.test(fm, 'tx', console=TRUE)

Groups, Treatments and means

a BE.Dry 33.25

ab F230.Dry 30

abc F230.Ice 25.88

abc BE.EtOH 25.38

abc BE.Ice 23.5

bc F230.EtOH 21.38

bc F230.Buffer 21.25

c BE.Buffer 18.75

> fm <- aov(N_Id_Metazoa ~ Amplicon * Filtration * Preservation, data = Lake)

> summary(fm)

Df Sum Sq Mean Sq F value Pr(>F)

Amplicon 1 0.56 0.56 0.182 0.671201

Filtration 1 196.00 196.00 63.568 2.42e-10 ***

Preservation 3 78.06 26.02 8.439 0.000131 ***

Amplicon:Filtration 1 20.25 20.25 6.568 0.013577 *

Amplicon:Preservation 3 4.31 1.44 0.466 0.707209

Filtration:Preservation 3 3.13 1.04 0.338 0.798033

Amplicon:Filtration:Preservation 3 9.62 3.21 1.041 0.383165

Residuals 48 148.00 3.08

---

Signif. codes: 0 ‘***’ 0.001 ‘**’ 0.01 ‘*’ 0.05 ‘.’ 0.1 ‘ ’ 1

> HSD.test(fm, 'Filtration', console=TRUE)

a direct 6.719

b pre 3.219

> HSD.test(fm, 'Preservation', console=TRUE)

Groups, Treatments and means

a Ice 6.062

a Dry 5.875

ab EtOH 4.625

b Buffer 3.312

> tx <- with(Lake, interaction(Amplicon, Filtration))

fm <- aov(N_Id_Metazoa ~ tx, data = Lake)

HSD.test(fm, 'tx', console=TRUE)

Groups, Treatments and means

a BE.direct 7.188

a F230.direct 6.25

b F230.pre 3.875

b BE.pre 2.562

> fm <- aov(S_Metazoa ~ Amplicon * Filtration * Preservation, data = Lake)

> summary(fm)

Df Sum Sq Mean Sq F value Pr(>F)

Amplicon 1 0.1188 0.11882 5.919 0.01875 *

Filtration 1 0.0900 0.08997 4.482 0.03946 *

Preservation 3 0.2840 0.09465 4.715 0.00579 **

Amplicon:Filtration 1 0.0182 0.01819 0.906 0.34588

Amplicon:Preservation 3 0.0942 0.03141 1.565 0.21017

Filtration:Preservation 3 0.1142 0.03807 1.897 0.14274

Amplicon:Filtration:Preservation 3 0.0595 0.01985 0.989 0.40606

Residuals 48 0.9635 0.02007

---

Signif. codes: 0 ‘***’ 0.001 ‘**’ 0.01 ‘*’ 0.05 ‘.’ 0.1 ‘ ’ 1

> HSD.test(fm, 'Amplicon', console=TRUE)

Groups, Treatments and means

a BE 0.7857

b F230 0.6996

> HSD.test(fm, 'Filtration', console=TRUE)

Groups, Treatments and means

a pre 0.7801

b direct 0.7052

> HSD.test(fm, 'Preservation', console=TRUE)

Groups, Treatments and means

a Dry 0.8324

ab EtOH 0.7779

b Ice 0.6977

b Buffer 0.6625

> fm <- aov(P_Metazoa ~ Amplicon * Filtration * Preservation, data = Lake)

> summary(fm)

Df Sum Sq Mean Sq F value Pr(>F)

Amplicon 1 0.2949 0.29487 20.175 4.44e-05 ***

Filtration 1 0.1918 0.19182 13.125 0.000702 ***

Preservation 3 0.1357 0.04524 3.096 0.035493 *

Amplicon:Filtration 1 0.0038 0.00383 0.262 0.611140

Amplicon:Preservation 3 0.0849 0.02829 1.935 0.136438

Filtration:Preservation 3 0.1095 0.03652 2.498 0.070769 .

Amplicon:Filtration:Preservation 3 0.0203 0.00678 0.464 0.708868

Residuals 48 0.7016 0.01462

---

Signif. codes: 0 ‘***’ 0.001 ‘**’ 0.01 ‘*’ 0.05 ‘.’ 0.1 ‘ ’ 1

> HSD.test(fm, 'Amplicon', console=TRUE)

Groups, Treatments and means

a BE 0.6793

b F230 0.5435

> HSD.test(fm, 'Filtration', console=TRUE)

Groups, Treatments and means

a pre 0.6661

b direct 0.5566

> HSD.test(fm, 'Preservation', console=TRUE)

Groups, Treatments and means

a Dry 0.6633

a EtOH 0.6477

a Ice 0.5843

a Buffer 0.5503

> fm <- update(fm, . ~ . -Amplicon:Filtration:Preservation)

> summary(fm)

Df Sum Sq Mean Sq F value Pr(>F)

Amplicon 1 0.2949 0.29487 20.832 3.19e-05 ***

Filtration 1 0.1918 0.19182 13.552 0.00056 ***

Preservation 3 0.1357 0.04524 3.196 0.03106 *

Amplicon:Filtration 1 0.0038 0.00383 0.270 0.60527

Amplicon:Preservation 3 0.0849 0.02829 1.998 0.12590

Filtration:Preservation 3 0.1095 0.03652 2.580 0.06364 .

Residuals 51 0.7219 0.01415

---

Signif. codes: 0 ‘***’ 0.001 ‘**’ 0.01 ‘*’ 0.05 ‘.’ 0.1 ‘ ’ 1

> fm1 <- update(fm, .~Amplicon+Filtration+Preservation)

> summary(fm1)

Df Sum Sq Mean Sq F value Pr(>F)

Amplicon 1 0.2949 0.29487 18.587 6.37e-05 ***

Filtration 1 0.1918 0.19182 12.092 0.000967 ***

Preservation 3 0.1357 0.04524 2.852 0.045026 *

Residuals 58 0.9201 0.01586

---

Signif. codes: 0 ‘***’ 0.001 ‘**’ 0.01 ‘*’ 0.05 ‘.’ 0.1 ‘ ’ 1

> fm2 <- update(fm1, . ~ . -Amplicon)

> summary(fm2)

Df Sum Sq Mean Sq F value Pr(>F)

Filtration 1 0.1918 0.19182 9.315 0.0034 **

Preservation 3 0.1357 0.04524 2.197 0.0979 .

Residuals 59 1.2150 0.02059

---

Signif. codes: 0 ‘***’ 0.001 ‘**’ 0.01 ‘*’ 0.05 ‘.’ 0.1 ‘ ’ 1

> fm <- aov(DB_Metazoa ~ Amplicon * Filtration * Preservation, data = Lake)

> summary(fm)

Df Sum Sq Mean Sq F value Pr(>F)

Amplicon 1 0.4325 0.4325 32.247 7.73e-07 ***

Filtration 1 0.0011 0.0011 0.084 0.77328

Preservation 3 0.1717 0.0572 4.267 0.00948 **

Amplicon:Filtration 1 0.0138 0.0138 1.030 0.31525

Amplicon:Preservation 3 0.0202 0.0067 0.502 0.68257

Filtration:Preservation 3 0.1450 0.0483 3.603 0.01990 *

Amplicon:Filtration:Preservation 3 0.0023 0.0008 0.058 0.98158

Residuals 48 0.6438 0.0134

---

Signif. codes: 0 ‘***’ 0.001 ‘**’ 0.01 ‘*’ 0.05 ‘.’ 0.1 ‘ ’ 1

> HSD.test(fm, 'Amplicon', console=TRUE)

Groups, Treatments and means

a BE 0.3634

b F230 0.199

> HSD.test(fm, 'Preservation', console=TRUE)

Groups, Treatments and means

a Buffer 0.3417

ab Ice 0.3235

b EtOH 0.232

b Dry 0.2276

> tx <- with(Lake, interaction(Filtration, Preservation))

fm <- aov(DB_Metazoa ~ tx, data = Lake)

HSD.test(fm, 'tx', console=TRUE)

Groups, Treatments and means

a pre.Ice 0.4029

a direct.Buffer 0.3928

a pre.Buffer 0.2906

a direct.Ice 0.244

a direct.Dry 0.2419

a pre.EtOH 0.2347

a direct.EtOH 0.2294

a pre.Dry 0.2134

> fm <- aov(DD_Metazoa ~ Amplicon * Filtration * Preservation, data = Lake)

> summary(fm)

Df Sum Sq Mean Sq F value Pr(>F)

Amplicon 1 0.00926 0.009257 1.817 0.1839

Filtration 1 0.02432 0.024320 4.775 0.0338 *

Preservation 3 0.00719 0.002396 0.470 0.7043

Amplicon:Filtration 1 0.00309 0.003093 0.607 0.4397

Amplicon:Preservation 3 0.01686 0.005621 1.104 0.3569

Filtration:Preservation 3 0.03073 0.010242 2.011 0.1249

Amplicon:Filtration:Preservation 3 0.00102 0.000340 0.067 0.9773

Residuals 48 0.24447 0.005093

---

Signif. codes: 0 ‘***’ 0.001 ‘**’ 0.01 ‘*’ 0.05 ‘.’ 0.1 ‘ ’ 1

> HSD.test(fm, 'Filtration', console=TRUE)

Groups, Treatments and means

a direct 0.2685

b pre 0.2295

> River <- read.csv("RiverANOVA.csv")

> str(River)

> fm <- aov(N_Metazoa ~ Amplicon * Filter * Preservation, data = River)

> summary(fm)

Df Sum Sq Mean Sq F value Pr(>F)

Amplicon 1 1139 1139 0.869 0.35587

Filter 1 54173 54173 41.334 5.52e-08 ***

Preservation 3 169445 56482 43.096 1.16e-13 ***

Amplicon:Filter 1 484 484 0.369 0.54625

Amplicon:Preservation 3 18114 6038 4.607 0.00652 **

Filter:Preservation 3 13760 4587 3.500 0.02238 *

Amplicon:Filter:Preservation 3 511 170 0.130 0.94174

Residuals 48 62909 1311

---

Signif. codes: 0 ‘***’ 0.001 ‘**’ 0.01 ‘*’ 0.05 ‘.’ 0.1 ‘ ’ 1

> HSD.test(fm, 'Filter', console=TRUE)

Groups, Treatments and means

a CN 324.5

b PES 266.3

> HSD.test(fm, 'Preservation', console=TRUE)

Groups, Treatments and means

a Dry 330.1

a Ice 324.2

a Buffer 320.9

b EtOH 206.5

> tx <- with(River, interaction(Amplicon, Preservation))

fm <- aov(N_Metazoa ~ tx, data = River)

HSD.test(fm, 'tx', console=TRUE)

Groups, Treatments and means

a BE.Buffer 334.9

a BE.Dry 333.8

a F230.Ice 327.4

a F230.Dry 326.5

a BE.Ice 321.1

ab F230.Buffer 306.9

bc F230.EtOH 237.9

c BE.EtOH 175.1

> tx <- with(River, interaction(Filter, Preservation))

fm <- aov(N_Metazoa ~ tx, data = River)

HSD.test(fm, 'tx', console=TRUE)

Groups, Treatments and means

a CN.Ice 364.9

a CN.Dry 362

a CN.Buffer 360.4

b PES.Dry 298.2

b PES.Ice 283.6

b PES.Buffer 281.4

c CN.EtOH 210.9

c PES.EtOH 202.1

> fm <- aov(N_Id_Metazoa ~ Amplicon * Filter * Preservation, data = River)

> summary(fm)

Df Sum Sq Mean Sq F value Pr(>F)

Amplicon 1 34457 34457 59.175 6.44e-10 ***

Filter 1 20485 20485 35.180 3.19e-07 ***

Preservation 3 70282 23427 40.233 3.79e-13 ***

Amplicon:Filter 1 11 11 0.020 0.889

Amplicon:Preservation 3 6370 2123 3.646 0.019 *

Filter:Preservation 3 6956 2319 3.982 0.013 *

Amplicon:Filter:Preservation 3 13 4 0.007 0.999

Residuals 48 27950 582

---

Signif. codes: 0 ‘***’ 0.001 ‘**’ 0.01 ‘*’ 0.05 ‘.’ 0.1 ‘ ’ 1

> HSD.test(fm, 'Amplicon', console=TRUE)

Groups, Treatments and means

a F230 219.4

b BE 173

> HSD.test(fm, 'Filter', console=TRUE)

Groups, Treatments and means

a CN 214.1

b PES 178.3

> HSD.test(fm, 'Preservation', console=TRUE)

Groups, Treatments and means

a Ice 217.8

a Dry 215.7

a Buffer 212.3

b EtOH 138.9

> tx <- with(River, interaction(Amplicon, Preservation))

fm <- aov(N_Id_Metazoa ~ tx, data = River)

HSD.test(fm, 'tx', console=TRUE)

Groups, Treatments and means

a F230.Dry 237.4

a F230.Ice 235.6

ab F230.Buffer 225.9

ab BE.Ice 200

ab BE.Buffer 198.8

ab BE.Dry 194

b F230.EtOH 178.6

c BE.EtOH 99.12

> tx <- with(River, interaction(Filter, Preservation))

fm <- aov(N_Id_Metazoa ~ tx, data = River)

HSD.test(fm, 'tx', console=TRUE)

Groups, Treatments and means

a CN.Ice 246.5

ab CN.Buffer 236.9

ab CN.Dry 232.6

ab PES.Dry 198.8

bc PES.Ice 189.1

bc PES.Buffer 187.8

c CN.EtOH 140.2

c PES.EtOH 137.5

> fm <- aov(S_Metazoa ~ Amplicon * Filter * Preservation, data = River)

> summary(fm)

Df Sum Sq Mean Sq F value Pr(>F)

Amplicon 1 0.0441 0.04405 2.492 0.121

Filter 1 0.0157 0.01573 0.890 0.350

Preservation 3 0.1047 0.03492 1.975 0.130

Amplicon:Filter 1 0.0031 0.00309 0.175 0.678

Amplicon:Preservation 3 0.0256 0.00853 0.483 0.696

Filter:Preservation 3 0.0825 0.02751 1.556 0.212

Amplicon:Filter:Preservation 3 0.0094 0.00313 0.177 0.911

Residuals 48 0.8484 0.01768

> fm <- aov(S_Id_Metazoa ~ Amplicon * Filter * Preservation, data = River)

> summary(fm)

Df Sum Sq Mean Sq F value Pr(>F)

Amplicon 1 0.0238 0.02382 1.277 0.2641

Filter 1 0.0165 0.01648 0.883 0.3520

Preservation 3 0.1294 0.04313 2.312 0.0879 .

Amplicon:Filter 1 0.0031 0.00308 0.165 0.6864

Amplicon:Preservation 3 0.0398 0.01328 0.712 0.5497

Filter:Preservation 3 0.0973 0.03244 1.739 0.1716

Amplicon:Filter:Preservation 3 0.0095 0.00316 0.169 0.9165

Residuals 48 0.8955 0.01866

---

Signif. codes: 0 ‘***’ 0.001 ‘**’ 0.01 ‘*’ 0.05 ‘.’ 0.1 ‘ ’ 1

> fm <- aov(P_Metazoa ~ Amplicon * Filter * Preservation, data = River)

> summary(fm)

Df Sum Sq Mean Sq F value Pr(>F)

Amplicon 1 0.3531 0.3531 31.326 1.03e-06 ***

Filter 1 0.0357 0.0357 3.163 0.0817 .

Preservation 3 0.0343 0.0114 1.014 0.3948

Amplicon:Filter 1 0.0001 0.0001 0.011 0.9170

Amplicon:Preservation 3 0.0177 0.0059 0.522 0.6692

Filter:Preservation 3 0.0388 0.0129 1.147 0.3396

Amplicon:Filter:Preservation 3 0.0049 0.0016 0.145 0.9322

Residuals 48 0.5411 0.0113

---

Signif. codes: 0 ‘***’ 0.001 ‘**’ 0.01 ‘*’ 0.05 ‘.’ 0.1 ‘ ’ 1

> HSD.test(fm, 'Amplicon', console=TRUE)

Groups, Treatments and means

a BE 0.6781

b F230 0.5295

> fm <- aov(P_Id_Metazoa ~ Amplicon * Filter * Preservation, data = River)

> summary(fm)

Df Sum Sq Mean Sq F value Pr(>F)

Amplicon 1 0.2777 0.27766 22.591 1.86e-05 ***

Filter 1 0.0382 0.03816 3.104 0.0844 .

Preservation 3 0.0344 0.01147 0.933 0.4320

Amplicon:Filter 1 0.0000 0.00003 0.003 0.9584

Amplicon:Preservation 3 0.0177 0.00591 0.480 0.6974

Filter:Preservation 3 0.0572 0.01908 1.552 0.2132

Amplicon:Filter:Preservation 3 0.0059 0.00197 0.160 0.9225

Residuals 48 0.5900 0.01229

---

Signif. codes: 0 ‘***’ 0.001 ‘**’ 0.01 ‘*’ 0.05 ‘.’ 0.1 ‘ ’ 1

> HSD.test(fm, 'Amplicon', console=TRUE)

Groups, Treatments and means

a BE 0.6717

b F230 0.54

> fm <- aov(DB_Metazoa ~ Amplicon * Filter * Preservation, data = River)

> summary(fm)

Df Sum Sq Mean Sq F value Pr(>F)

Amplicon 1 0.1311 0.13106 8.830 0.00462 **

Filter 1 0.0056 0.00556 0.375 0.54327

Preservation 3 0.2387 0.07955 5.360 0.00289 **

Amplicon:Filter 1 0.0055 0.00548 0.369 0.54641

Amplicon:Preservation 3 0.0397 0.01324 0.892 0.45192

Filter:Preservation 3 0.1471 0.04905 3.305 0.02793 *

Amplicon:Filter:Preservation 3 0.0104 0.00347 0.234 0.87230

Residuals 48 0.7124 0.01484

---

Signif. codes: 0 ‘***’ 0.001 ‘**’ 0.01 ‘*’ 0.05 ‘.’ 0.1 ‘ ’ 1

> HSD.test(fm, 'Amplicon', console=TRUE)

Groups, Treatments and means

a BE 0.2265

b F230 0.136

> HSD.test(fm, 'Preservation', console=TRUE)

Groups, Treatments and means

a EtOH 0.2834

b Buffer 0.1616

b Ice 0.1584

b Dry 0.1215

> tx <- with(River, interaction(Filter, Preservation))

fm <- aov(DB_Metazoa ~ tx, data = River)

HSD.test(fm, 'tx', console=TRUE)

Groups, Treatments and means

a CN.EtOH 0.3563

ab PES.EtOH 0.2106

ab PES.Ice 0.2057

ab PES.Buffer 0.1965

b PES.Dry 0.1495

b CN.Buffer 0.1268

b CN.Ice 0.1111

b CN.Dry 0.09348

> fm <- aov(DB_Id_Metazoa ~ Amplicon * Filter * Preservation, data = River)

> summary(fm)

Df Sum Sq Mean Sq F value Pr(>F)

Amplicon 1 0.0998 0.09976 5.983 0.01816 *

Filter 1 0.0029 0.00288 0.173 0.67937

Preservation 3 0.2805 0.09349 5.607 0.00223 **

Amplicon:Filter 1 0.0025 0.00253 0.152 0.69882

Amplicon:Preservation 3 0.0506 0.01685 1.011 0.39625

Filter:Preservation 3 0.1557 0.05191 3.113 0.03478 *

Amplicon:Filter:Preservation 3 0.0135 0.00450 0.270 0.84662

Residuals 48 0.8004 0.01668

---

Signif. codes: 0 ‘***’ 0.001 ‘**’ 0.01 ‘*’ 0.05 ‘.’ 0.1 ‘ ’ 1

> HSD.test(fm, 'Amplicon', console=TRUE)

Groups, Treatments and means

a BE 0.2063

b F230 0.1273

> HSD.test(fm, 'Preservation', console=TRUE)

Groups, Treatments and means

a EtOH 0.2782

b Ice 0.1427

b Buffer 0.1426

b Dry 0.1038

> tx <- with(River, interaction(Filter, Preservation))

fm <- aov(DB_Id_Metazoa ~ tx, data = River)

HSD.test(fm, 'tx', console=TRUE)

Groups, Treatments and means

a CN.EtOH 0.3559

ab PES.EtOH 0.2005

ab PES.Ice 0.1889

ab PES.Buffer 0.1762

b PES.Dry 0.1286

b CN.Buffer 0.109

b CN.Ice 0.09648

b CN.Dry 0.07912

> fm <- aov(DD_Metazoa ~ Amplicon * Filter * Preservation, data = River)

> summary(fm)

Df Sum Sq Mean Sq F value Pr(>F)

Amplicon 1 0.00348 0.003480 4.952 0.0308 *

Filter 1 0.00207 0.002068 2.944 0.0927 .

Preservation 3 0.01176 0.003919 5.578 0.0023 **

Amplicon:Filter 1 0.00057 0.000569 0.810 0.3727

Amplicon:Preservation 3 0.00091 0.000303 0.432 0.7312

Filter:Preservation 3 0.00286 0.000955 1.359 0.2666

Amplicon:Filter:Preservation 3 0.00017 0.000056 0.080 0.9706

Residuals 48 0.03373 0.000703

---

Signif. codes: 0 ‘***’ 0.001 ‘**’ 0.01 ‘*’ 0.05 ‘.’ 0.1 ‘ ’ 1

> HSD.test(fm, 'Amplicon', console=TRUE)

Groups, Treatments and means

a BE 0.2945

b F230 0.2797

> HSD.test(fm, 'Preservation', console=TRUE)

Groups, Treatments and means

a EtOH 0.3085

ab Ice 0.2885

b Buffer 0.2785

b Dry 0.2729

> fm <- aov(DD_Id_Metazoa ~ Amplicon * Filter * Preservation, data = River)

> summary(fm)

Df Sum Sq Mean Sq F value Pr(>F)

Amplicon 1 0.00038 0.000381 0.516 0.47606

Filter 1 0.00161 0.001611 2.179 0.14641

Preservation 3 0.01209 0.004031 5.454 0.00262 **

Amplicon:Filter 1 0.00015 0.000149 0.201 0.65573

Amplicon:Preservation 3 0.00106 0.000354 0.479 0.69808

Filter:Preservation 3 0.00288 0.000961 1.301 0.28496

Amplicon:Filter:Preservation 3 0.00034 0.000114 0.154 0.92645

Residuals 48 0.03547 0.000739

---

Signif. codes: 0 ‘***’ 0.001 ‘**’ 0.01 ‘*’ 0.05 ‘.’ 0.1 ‘ ’ 1

> HSD.test(fm, 'Preservation', console=TRUE)

Groups, Treatments and means

a EtOH 0.2567

ab Ice 0.2374

b Buffer 0.2271

b Dry 0.2202

2-way PERMANOVA results

Bray-Curtis similarity Sørensen dissimilarity

River F230-Metazoa

2-way PERMANOVA Filter: F=3.59 p=2.0e-04 Filter: F=1.40 p=0.013

Preservation F=1.36 p=5.0e-04

Pairwise comparisons Buffer Dry EtOH Ice

Bonferroni-corrected Buffer 1 0.04 1

p-values Dry 0.007 1

EtOH <0.001

Ice

River BE-Metazoa

2-way PERMANOVA Filter F=2.48 p=0.007 Filter F=1.47 p=0.035

Preservation F=3.86 p=1.0e-04 Preservation F=2.18 p=1.0e-04

Pairwise comparisons Buffer Dry EtOH Ice Buffer Dry EtOH Ice

Bonferroni-corrected Buffer 0.66 0.001 0.87 Buffer 1 0.003 1

p-values Dry 0.001 0.20 Dry 0.002 1

EtOH 0.002 EtOH <0.001

Ice Ice

River DNA-species

2-way PERMANOVA Filter F=3.66 p=1.0e-04 Filter F=1.90 p=0.005

Preservation F=1.77 p=7.0e-04 Preservation F=1.68 p=3.0e-04

Interaction F=1.47 p=0.009

Pairwise comparisons Buffer Dry EtOH Ice Buffer Dry EtOH Ice

Bonferroni-corrected Buffer 1 0.30 0.57 Buffer 1 0.01 1

p-values Dry 0.24 1 Dry 0.008 1

EtOH 0.002 EtOH 0.004

Ice Ice

Lake F230-Metazoa

2-way PERMANOVA Filtration F=21.64 p=1.0e-04 Filtration F=9.04 p=1.0e-04

Preservation F=2.07 p=0.031 Preservation F=2.00 p=0.003

no pair-wise differences in preservation no pair-wise differences in preservation

Lake BE-Metazoa

2-way PERMANOVA Filtration F=8.57 p=1.0e-04 Filtration F=7.76 p=1.0e-04

Preservation F=1.98 p=0.004 Preservation F=2.54 p=1.0e-04

Pairwise comparisons Buffer Dry EtOH Ice Buffer Dry EtOH Ice

Bonferroni-corrected Buffer 0.76 1 1 Buffer 0.002 0.01 1

p-values Dry 1 0.04 Dry 1 0.13

EtOH 0.13 EtOH 0.35

Ice Ice
